# Antiviral Role of Serine Incorporator 5 (SERINC5) Proteins in Classical Swine Fever Virus Infection

**DOI:** 10.3389/fmicb.2020.580233

**Published:** 2020-09-04

**Authors:** Wenhui Li, Zilin Zhang, Liangliang Zhang, Hong Li, Shuangqi Fan, Erpeng Zhu, Jindai Fan, Zhaoyao Li, Wenxian Chen, Lin Yi, Hongxing Ding, Jinding Chen, Mingqiu Zhao

**Affiliations:** ^1^College of Veterinary Medicine, South China Agricultural University, Guangzhou, China; ^2^Guangdong Laboratory for Lingnan Modern Agriculture, Guangzhou, China; ^3^Shandong Qianxi Agriculture & Animal Husbandry Development Co., Ltd., Zaozhuang, China

**Keywords:** classical swine fever virus, antiviral proteins, type I interferon, SERINC5, MDA5

## Abstract

Serine incorporator 5 (SERINC5), a multipass transmembrane protein, protects cells from viral infections. The mechanism by which SERINC5 protects against classical swine fever virus (CSFV) infection is unknown. In this study, overexpression of SERINC5 in PK-15 and 3D4/2 cells significantly inhibited the growth of CSFV, whereas SERINC5 silencing enhanced CSFV growth. Additionally, CSFV infection reduced SERINC5 production in cells and tissues. Liquid chromatography-tandem mass spectrometry (LC-MS/MS) was used to identify and analyze protein and peptide molecules that potentially interact with SERINC5. A total of 33 cellular protein candidates were identified. Next, SERINC5 was shown to interact with melanoma differentiation-associated protein 5 (MDA5) by yeast two-hybrid, protein co-localization and co-immunoprecipitation assays. Furthermore, SERINC5 enhanced MDA5-mediated type I interferon (IFN) signaling in a dose-dependent manner. Our results suggest that the anti-CSFV effect of SERINC5 is dependent on the activation of the type I IFN, which may function along with MDA5. The inhibitory effect of SERINC5 on CSFV was disappeared when the endogenous expression of MDA5 was silenced using siRNA, suggesting that SERINC5 exerts an anti-CSFV effect in an MDA5-dependent manner. Our study demonstrated a novel link between SERINC5 and MDA5 in the inhibition of CSFV replication via the type I IFN signaling pathway.

## Introduction

Classical swine fever (CSF), caused by classical swine fever virus (CSFV), is a highly contagious viral disease of pigs listed by the World Organization for Animal Health (OIE) (Ali et al., 1999; Becher et al., 2003). The genome of CSFV consists of a 12.3 kb single-stranded positive-sense RNA, which encodes 12 proteins, including four structural proteins (C, Erns, E1 and E2) and eight non-structural proteins (Npro, p7, NS2, NS3, NS4A, NS4B, NS5A, and NS5B) (Elbers et al., 1996; Lefkowitz et al., 2017). Infected animals generally show different clinical signs, including high fever, respiratory and gastrointestinal signs, hemorrhage, leukopenia, abortions, neurological dysfunction and high mortality, which brings enormous loss to the pig industry worldwide (Kleiboeker, 2002; Lohse et al., 2012). Although extensive efforts have been made to control the spread of CSF disease through stamping-out (non-vaccination) and mass vaccination strategies, there are no specific therapeutic drugs currently available. Previous studies have found that low-virulence CSFV strains can cause chronic and subclinical infections, significantly complicating the control of CSF (Ganges et al., 2008; Sara et al., 2015). To effectively control CSFV infection through drugs or vaccines, a better understanding of the relationship between CSFV and host is required. Although the mechanism of CSFV replication has been well characterized, the current understanding of CSFV pathogenesis is still limited.

Classical swine fever virus has a unique tropism for hematopoietic and immune cells, resulting in severe leukopenia and cellular immunosuppression (Ji et al., 2015). The innate cellular immune response represents the first line of defense against pathogen invasion. By sensing pattern recognition receptors (PRRs), pathogens can be detected by conserved molecular structures, known as pathogen-associated molecular patterns (PAMPs), which are unique to microbial organisms and critical to their life cycle (Wu and Chen, 2014). For example, retinoic acid-induced gene I (RIG-I) encoded by DExD/H-box helicase 58 (DDX58) gene in the human genome (Mao et al., 1996; Imaizumi et al., 2002) and melanoma differentiation-associated protein 5 (MDA5) encoded by interferon-induced helicase (IFIH1) gene (Kang et al., 2002; Kovacsovics et al., 2002) are referred to as the RIG-I-like receptors (RLRs). Activation of this PRRs results in the induction of type I interferon (IFNα/β), pro-inflammatory cytokines and chemokines, and expression of antimicrobial genes during the early stages of viral infection (Zhao and Karijolich, 2019). These genes mediate pathogen infection and vasodilatation near the site of insult to aid in the recruitment of immune cells, thereby establishing an immune-regulating and antiviral state (Brisse and Ly, 2019). Both RIG-I and MDA5 are important proteins, which initiate early immune responses to virus infection. MDA5 was discovered as an interferon-induced putative RNA helicase exhibiting ATPase activity and melanoma growth inhibitory properties in human melanoma cells (Kang et al., 2002). In addition, MDA5 plays an important role in identifying viral infections and transmitting signals through the caspase recruitment domain (CARD) (Kato et al., 2005; Gitlin et al., 2006). There are many similarities between RIG-I and MDA5 in heredity, structure, function and the signals initiated through the universal adapter mitochondrial antiviral signaling protein (MAVS) protein. RIG-1 and MDA5 have multiple redundant immunity mechanisms to protect the host (Kasumba and Grandvaux, 2018). These proteins have unique strategies to identify different host species, PAMPs and different pathogens (Brisse and Ly, 2019). For example, RIG-1 triggers the assembly of inflammatory bodies to activate caspase-1 dependent cleavage of pro-IL-1β, leading to efflux of mature IL-1β (Loo et al., 2008; Loo and Gale, 2011; Errett et al., 2013). In the case of Sendai virus (SeV) infection, IRF-3 activation is dependent on RIG-1, whereas MDA5 functions to inhibit ubiquitination and proteasome-mediated degradation of IRF-3 (Grandvaux et al., 2014).

The concept of “restriction factors” first appeared in the 1970s (Frank, 1970). Although there is no clear definition of the restriction factor, they are known as a class of proteins in cells that limit viral replication as a host defense measure (Doyle et al., 2015). Recently, there has been increased interest in research examining antiviral restriction factors, including APOBEC3, TRIM, and IFITM families, in addition to ISG15, SERINC3/5 (Stremlau et al., 2004; Zheng et al., 2004; Pincetic et al., 2010; Lu et al., 2011). These restriction factors can inhibit the virus through various mechanisms at various stages of the viral replication process (Evans and Liu, 2020). Most of these proteins are antagonized by viral factors and induced in response to the expression of type I and III interferons (Harris et al., 2012; Kluge et al., 2015). Serine incorporator (SERINC) is a newly discovered rare host-restricted cytokine, which is not induced by IFN (Usami et al., 2015; Ziglio et al., 2015). The SERINC proteins belong to a family of multi-transmembrane carrier proteins, which are highly conserved across species (Inuzuka et al., 2005; Bossolasco et al., 2006). SERINC functions by integrating serine into the lipid bilayer to synthesize phosphatidylserine (PS) and sphingolipids. It is a key component of membrane phospholipids indispensable for cell functions (Inuzuka et al., 2005). However, the physiological function of the SERINC protein family remains largely unknown (Oliver, 2015; Trautz et al., 2017). As an important family of transmembrane proteins, SERINC contains five members (SERINCs 1-5). Only SERINC3 and SERINC5 of SERINC family can inhibit viral infection through the prevention of viral fusion and act as restriction factors early in the viral life cycle (Usami et al., 2015; Ziglio et al., 2015). The human SERINC5 protein consists of 423 amino acids, which was discovered through protein structure prediction. It was predicted to be a type III cell membrane protein with 10 to 12 transmembrane domains (Inuzuka et al., 2005). It has also been reported that SERINC5 splice isomers encoding 10 transmembrane domain proteins are highly expressed and exhibit potent antiviral activity (Zhang et al., 2017). Another study reported that SERINC5 affected the cytoplasmic transfer of reporter proteins, thereby reducing virus-cell fusion (Usami et al., 2015; Ziglio et al., 2015). However, the molecular mechanism by which SERINC5 prevents viral infiltration remains unclear (Firrito et al., 2018).

In the present study, the role of SERINC5 on the MDA5-mediated signaling pathway in CSFV infection was examined. It was observed that SERINC5 markedly repressed CSFV infection and replication, whereas knockdown of SERINC5 expression in PK-15 and 3D4/2 cells restored viral replication. Furthermore, direct binding of SERINC5 to MDA5 suppressed CSFV replication via the MDA5-mediated type I IFN-signaling pathway.

## Materials and Methods

### Cells and Viruses

The swine kidney cell line PK-15 (ATCC, CCL-33) and the human embryonic kidney cell line HEK-293T (ATCC, CRL-11268) were grown in DMEM (Gibco, C11995500BT) supplemented with 10% FBS (Gibco, 10091-148) in a humidified 5% CO_2_ environment at 37°C. The swine macrophage cell line 3D4/2 (ATCC, CRL-2845) was grown in RPMI 1640 medium (Gibco, C11875500BT) supplemented with 10% FBS (Gibco, 10091148) in a humidified 5% CO_2_ environment at 37°C. The Shimen strain of CSFV strain was propagated in PK-15 cells. Virus titers were determined as previously described (Gou et al., 2017). SeV was propagated in specific-pathogen-free chicken embryos. Experiments involving CSFV were carried out according to the institutionally approved Laboratory Biosafety Manual, and were carried out in the laboratory animal center of South China Agricultural University.

### Antibodies, Reagents, Plasmids and siRNAs

The primary antibodies used in the study were as follows: rabbit polyclonal anti-SERINC5 (Abcam, ab204400), mouse monoclonal anti-CSFV E2 (JBT, 9011), mouse monoclonal anti-GAPDH (Beyotime, AG019) and mouse polyclonal anti-CSFV Npro. The secondary antibodies were used in the study were as follows: Alexa Fluor 488 goat anti-mouse IgG (H + L) (Beyotime, A0428), and Alexa Fluor 647 goat anti-rabbit IgG (H + L) (Beyotime, A0468) were used for immunofluorescence analysis. Goat anti-mouse-FITC (Beyotime, A0568) was used for indirect immunofluorescence assay (IFA). Goat anti-mouse-horseradish peroxidase (HRP) conjugated IgG (H + L) (Beyotime, A0216) and HRP-conjugated goat anti-rabbit IgG (H + L) (Beyotime, A0208) were used for immunoblotting analysis. The antiviral PRRs TLR3, RIG-I/MDA5 and their activator Poly (I:C) (LMW) Rhodamine (tlrl-picw), and 5′ppp-dsRNA (tlrl-3prna), a synthetic ligand of RIG-I were purchased from Invivogen.

The plasmids, including ISRE-luc, NF-κB-luc, IFNβ-luc, pRL-TK, and pMD18-T-NS5B, were prepared in our laboratory. Small interfering RNAs (siRNAs) for SERINC5, MDA5, and RIG-I were synthesized by Sangon Biotech, Shanghai, China. The siRNA sequences are presented in Supplementary Table S1. The siRNAs were transfected into PK-15 and 3D4/2 cells upon reaching a cellular density of 70% confluence in a 12-well plate using the Lipofectamine 3000 reagent (Thermo Fisher, L3000015) according to the manufacturer’s instructions. The success of the silencing of protein was evaluated by western blotting and real-time quantitative polymerase chain reaction (qRT-PCR).

### Virus Infection

PK-15 and 3D4/2 cells were cultured to approximately 70% confluence in cell culture plates, and then infected with 1 multiplicity of infection (MOI) of CSFV at 37°C with in a humidified 5% CO_2_ environment for 1.5 h. The viral inoculum was discarded, and the cells were washed twice with phosphate-buffered saline (PBS) (pH 7.4). Next, the cells were incubated in DMEM containing 2% FBS at 37°C with 5% CO_2_ for different time points until harvesting. Aliquots of culture supernatants were stored at −80°C for further analysis.

### Cell Viability Assay

Cell viability was evaluated using the CCK8 kit (Beyotime, C0038) according to the manufacturer’s instructions. PK-15 and 3D4/2 cells were grown in 96-well plates at a seeding density of 1 × 10^4^ cells per well and cultured at 37°C with 5% CO_2_ for 24 h. The cells were transfected with p3 × Flag-CMV, p3 × Flag-SERINC5, siNC, or siSERINC5 using the Lipofectamine 3000 reagent. The cells were incubated for 48 h. Next, the medium was replaced with 100 μl of fresh medium containing 10 μl CCK8 and cultured at 37°C for 1 h. The optical density was measured at a wavelength of 540 nm using a Bio-Rad model 680 microplate reader (Bio-Rad).

### qRT-PCR

Total RNA of CSFV-infected cells and porcine tissue samples was isolated using TRIzol reagent (Invitrogen, 15596026) according to the manufacturer’s instructions. The cDNA was synthesized using the PrimeScript RT reagent kit (Takara, RR047A). Then, the cDNA was used as the template for qRT-PCR. Amplification and analyses were carried out using qPCR SYBR Green Master Mix (YEASEN, 11199ES03) in a Bio-Rad CFX96 Real-Time PCR System (Bio-Rad, United States). GAPDH was selected as an internal reference gene. Transcript levels were calculated using the 2^–ΔΔ*Ct*^ method (Livak and Schmittgen, 2001). The viral copy numbers in each sample were calculated from a recombinant plasmid containing the CSFV NS5B gene at known quantities to produce the standard curve. All primers used for qRT-PCR are presented in Supplementary Table S1. Each sample was assayed in triplicate.

### Identification of SERINC5-Associated Binding Proteins by Liquid Chromatography-Tandem Mass Spectrometry (LC-MS/MS)

The plasmid pEGFP-SERINC5 and the empty vector were respectively stably transfected into PK-15 cells and infected with 0.1 MOI of CSFV. The samples were then collected at different time points (12, 24, 36, and 48 h). The cell samples were immunoprecipitated using an anti-GFP mAb (Beyotime, AF0159) and protein A + G Agarose beads (Beyotime, P2012) at 4°C. The immunoprecipitated mixtures were washed four times with PBS (pH 7.4) and subsequently analyzed by Liquid chromatography-tandem mass spectrometry (LC-MS/MS) (Shanghai Applied Protein Technology Co., Ltd., China).

### Co-immunoprecipitation (Co-IP) and Western Blotting

For Flag-SERINC5 immunoprecipitation, HEK-293T cells were transfected with Flag-SERINC5 and HA-MDA5 for 24 h. The cell membranes were disrupted using IP lysis buffer (Beyotime, P0013) containing 1 mM PMSF (Beyotime, ST506) for 20 min at 4°C prior to harvest. Cell lysates were centrifuged at 13,000 × *g* for 20 min at 4°C. Clarified lysates were incubated with protein A + G Agarose beads at 4°C for 4 h and then immunoprecipitated with anti-Flag mAb (Beyotime, AF519) at 4°C for 5 h. The immunoprecipitated mixtures were washed four times with PBS (pH 7.4) and analyzed by western blotting with anti-HA mAb (Beyotime, AF2305).

For western blot analysis, tissue samples were harvested and lysed on ice with 200 μl of RIPA cell lysis buffer (Beyotime, P0013B) containing 4 μl protease and phosphatase inhibitor cocktail (50×) (Beyotime, P1051) for 20 min. The cell culture samples were harvested and lysed on ice with 200 μl of RIPA cell lysis buffer containing 1 mM PMSF for 20 min. The supernatant protein concentration was measured using a BCA protein assay kit (Thermo Fisher, 23225). Next, the lysates were centrifuged at 13,000 × *g* for 20 min at 4°C. The equal amounts of protein samples was boiled in 5 × SDS-PAGE loading buffer for 10 min. Protein bands were resolved on a 12.5% SDS-PAGE and transferred onto polyvinylidene fluoride or polyvinylidene difluoride (PVDF) membranes (Bio-Rad). The membranes were then blocked with 5% skim milk containing 0.05% Tween 20 (Sigma-Aldrich, 8221841000) at room temperature for 2 h, and then incubated at 4°C overnight with primary antibodies. After washing five times with tris buffered saline and tween (TBST), the membranes were incubated with appropriate HRP-conjugated secondary antibodies (diluted 1:2000 in PBST) for 2 h at 37°C. After incubating with the enhanced chemiluminescence (ECL) kit (Beyotime, P0018S), the signal was detected using the CanoScan LiDE 100 scanner (Canon, Japan). Images were analyzed using ImageJ software.

### Yeast Two-Hybrid

The Y2H assay was carried out with the Matchmaker Gold Yeast Two-Hybrid System (Clontech, 630489). The coding regions of SERINC5 and MDA5 were cloned into the bait pGBKT7 and prey pGADT7 plasmid vectors, respectively. All primers used to construct the vectors are presented in Supplementary Table S1. The self-activation ability of bait was tested according to the manufacturer’s instructions. The bait and prey plasmid pairs were introduced into the GoldY2H yeast strain to determine potential interactions.

### Confocal Microscopy

Cells were cultured to a density of 20–30% confluence in 35 mm cell culture dishes before co-transfection with Flag-SERINC5 and HA-MDA5. The cells were then incubated for 24 h. After washing three times with TBST, the cells were fixed using 4% paraformaldehyde for 30 min at 4°C, permeabilized with 0.1% Triton X-100 (Beyotime, ST795) for 10 min and blocked with 5% skim milk for 30 min. After washing five times, the cells were incubated at 4°C overnight with anti-Flag mAb and anti-HA mAb, respectively, and stained with appropriate Alexa Fluor 488- or 647-conjugated secondary antibodies for 1 h at 37°C. The cell nuclei were labeled with DAPI (Beyotime, C1002) at 25°C for 10 min. Then, the samples were observed on a confocal fluorescence microscope (Leica TCS SP2).

### Dual Luciferase Reporter Assay

HEK-293T (4 × 10^5^) cells were plated in 12-well plates and transfected with plasmids encoding ISRE, IFNβ, or NF-κB cloned into firefly or renilla luciferase reporter (100 and 10 ng, respectively) along with a plasmid encoding Flag-SERINC5. Transfection was accomplished using Lipofectamine 2000. The cells were infected with SeV, stimulated with poly (I:C), or activated by plasmid-based overexpression of HA-RIG-I, HA-MDA5, HA-MAVS, HA-TBK1, HA-IRF3 or HA-IRF7 for 24 h. The cells were collected, and reporter activities were analyzed using the Dual-Luciferase Assay reagents (Promega, E1910) in the Luminoskan Ascent Microplate Luminometer (Thermo, 2805621). The luciferase activities were calculated as the ratio of LUC to REN.

### Animal Experiments

The animal infection experiment was performed as described by Zhu et al. (2019). In brief, ten 2-month-old healthy pigs were randomly divided into two groups of five each. One group was infected with 10^5^ median tissue culture infectious dose (TCID_50_) of CSFV-Shimen strain. In another group, PBS was used as a control. Clinical signs and rectal temperatures of pigs were monitored and recorded daily until 7 days post-infection (dpi). Animals were euthanized, and heart, liver, spleen, lung, kidney, brain, inguinal lymph nodes, mesenteric lymph nodes, thymus, and tonsil samples were collected for analysis. The expression level of SERINC5 was measured by western blotting and qRT-PCR. All animal experiments were conducted following the Guide for the Care and Use of Laboratory Animals of South China Agricultural University, Guangzhou, China.

### Statistical Analysis

The data are presented as the mean ± standard deviation (SD) and were analyzed by one-way analysis of variance (ANOVA) using the Statistical Package for the Social Sciences (SPSS) 16.0 software. Graphs were created using the SigmaPlot 12.0 software. A value of *P* < 0.05 was considered to be statistically significant. Three independent biological replicates were performed during the experiment.

## Results

### Inhibition of CSFV Replication by SERINC5

**FIGURE 1:**
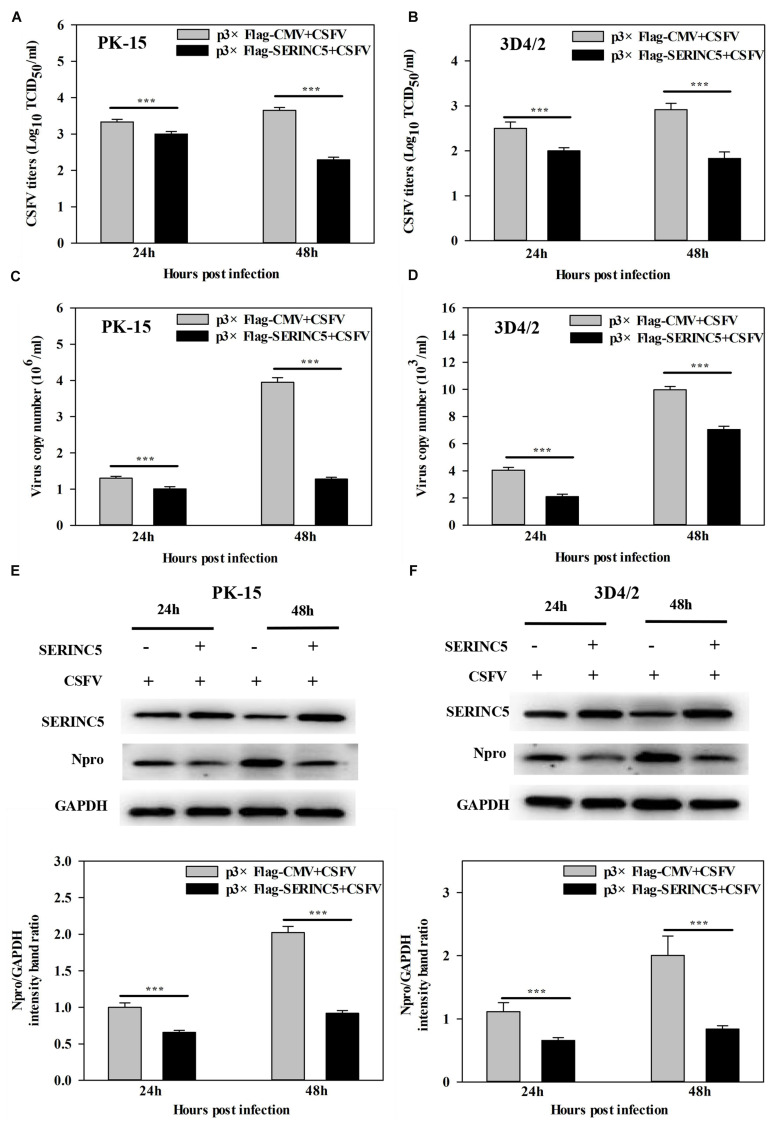
Serine incorporator 5 (SERINC5) inhibited CSFV growth in PK-15 **(A)** and 3D4/2 **(B)** cells. Cells were infected with CSFV (MOI = 0.1) for 24 and 48 h after transfection with p3 × Flag-SERINC5. Statistical analysis of the influence of overexpression of SERINC5 on viral genome replication in PK-15 **(C)** and 3D4/2 **(D)** cells. Statistical analysis of the influence of SERINC5 on CSFV Npro protein expression in PK-15 **(E)** and 3D4/2 **(F)** cells. The expression of CSFV Npro protein was analyzed by western blotting using anti-CSFV Npro antibody. GAPDH was used as an internal citation control. The relative levels of the targeted proteins were analyzed with ImageJ software, and the ratios were calculated relative to the GAPDH control. Error bars represent the mean ± SD; *n* = 3; ^∗^*P* < 0.05; ^∗∗^*P* < 0.01; ^∗∗∗^*P* < 0.001; ^NS^*P* > 0.05.

To determine the possible effect of SERINC5 on CSFV replication, PK-15 and 3D4/2 cells overexpressing SERINC5 and p3 × Flag-CMV, were infected with CSFV (MOI of 0.1) for 24 and 48 h. The viral titers, viral genome copy numbers and Npro protein expression were analyzed by IFA, qRT-PCR and western blotting, respectively. Viral titers, genome copy numbers and Npro protein expression were severely decreased after transfection with p3 × Flag-SERINC5 in PK-15 (Figures 1A,C,E) and 3D4/2 cells (Figures 1B,D,F). These results demonstrate an inhibitory effect of SERINC5 on CSFV replication.

**FIGURE 2:**
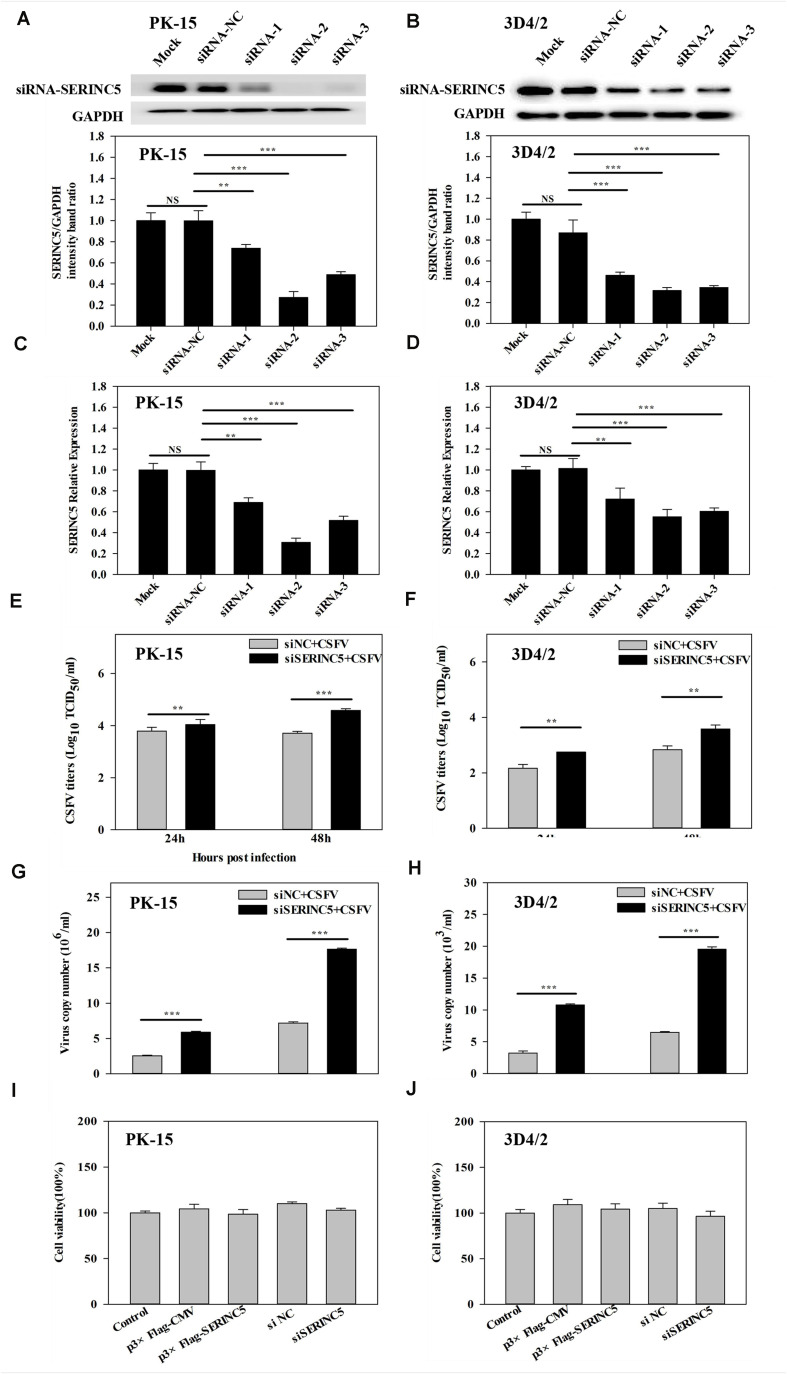
The RNA interference of SERINC5 enhanced CSFV replication in PK-15 and 3D4/2 cells. siRNA knockdown of SERINC5 in PK-15 **(A,C)** and 3D4/2 **(B,D)** cells transfected with siNC or SERINC5 siRNA-1/-2/-3. The expression of SERINC5 was assessed by western blotting **(A,B)** and qRT-PCR **(C,D)** at 24 hpi. PK-15 **(E,G)** and 3D4/2 **(F,H)** cells were infected with CSFV (MOI = 0.1) for 24 and 48 h after transfection with SERINC5 siRNA. Statistical analysis of the influence of SERINC5 siRNA on viral titers **(E,F)** and viral genome replication **(G,H)**. The effect of overexpression and siRNA on cell viability of PK-15 **(I)** and 3D4/2 **(J)** cells. Cells were transfected with p3 × Flag-CMV, p3 × Flag-SERINC5, siNC and SERINC5 siRNA were evaluated by CCK-8 assay. The relative levels of the targeted proteins were analyzed with ImageJ software, and the ratios were calculated relative to the GAPDH control. Error bars represent the mean ± SD; *n* = 3; ^∗^*P* < 0.05; ^∗∗^*P* < 0.01; ^∗∗∗^*P* < 0.001; ^NS^*P* > 0.05.

**FIGURE 3:**
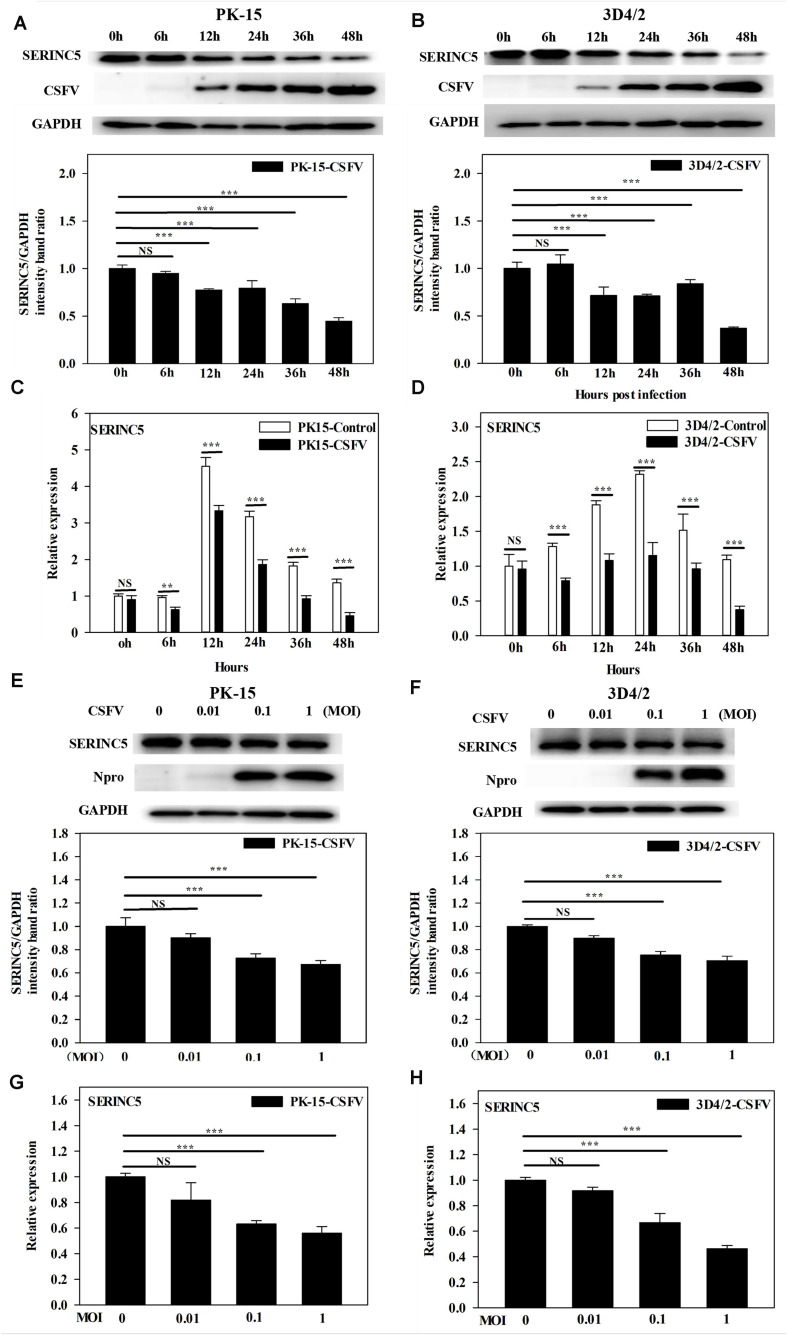
Classical swine fever virus (CSFV) infection downregulates SERINC5 expression in cells. Statistical analysis of the SERINC5 protein and transcription in PK-15 **(A,C)** and 3D4/2 **(B,D)** cells infected with CSFV (MOI = 0.1) for 0, 6, 12, 24, 36, and 48 h by western blotting **(A,B)** and qRT-PCR **(C,D)**. Statistical analysis of SERINC5 protein and transcription in CSFV infected PK-15 **(E,G)** and 3D4/2 **(F,H)** cells at an MOIs of 0, 0.01, and 0.1 for 24 h by western blotting **(E,F)** and qRT-PCR **(G,H)**. The relative levels of the targeted proteins were analyzed with ImageJ software, and the ratios were calculated relative to the GAPDH control. Error bars represent the mean ± SD; *n* = 3; ^∗^*P* < 0.05; ^∗∗^*P* < 0.01; ^∗∗∗^*P* < 0.001; ^NS^*P* > 0.05.

To further investigate the anti-CSFV effects of SERINC5, three siRNAs were used to knockdown the expression of SERINC5 in PK-15 and 3D4/2 cells. The silencing effect on SERINC5 protein and gene expression was confirmed by western blotting and qRT-PCR in Figures 2A–D, respectively. The cells were infected with CSFV (MOI of 0.1) for 24 and 48 h after SERINC5 silencing. The viral titers and genome copy numbers of CSFV were analyzed by IFA and qRT-PCR, respectively. Knockdown of SERINC5 expression, which increased CSFV titers and genome replication, was significantly increased in PK-15 (Figures 2E,G) and 3D4/2 cells (Figures 2F,H). These data support the inhibitory effect of SERINC5 on CSFV replication.

To exclude the possibility that p3 × Flag-SERINC5 and siSERINC5 inhibited or promoted CSFV replication by influencing host cell health, the effects of overexpression and RNA interference of SERINC5 on the viability of PK-15 and 3D4/2 cells were assessed. However, p3 × Flag-CMV, p3 × Flag-SERINC5, siNC and siSERINC5 did not significantly alter cell viability (Figures 2I,J).

### CSFV Infection Inhibits SERINC5 Expression *in vitro* and *in vivo*

**FIGURE 4:**
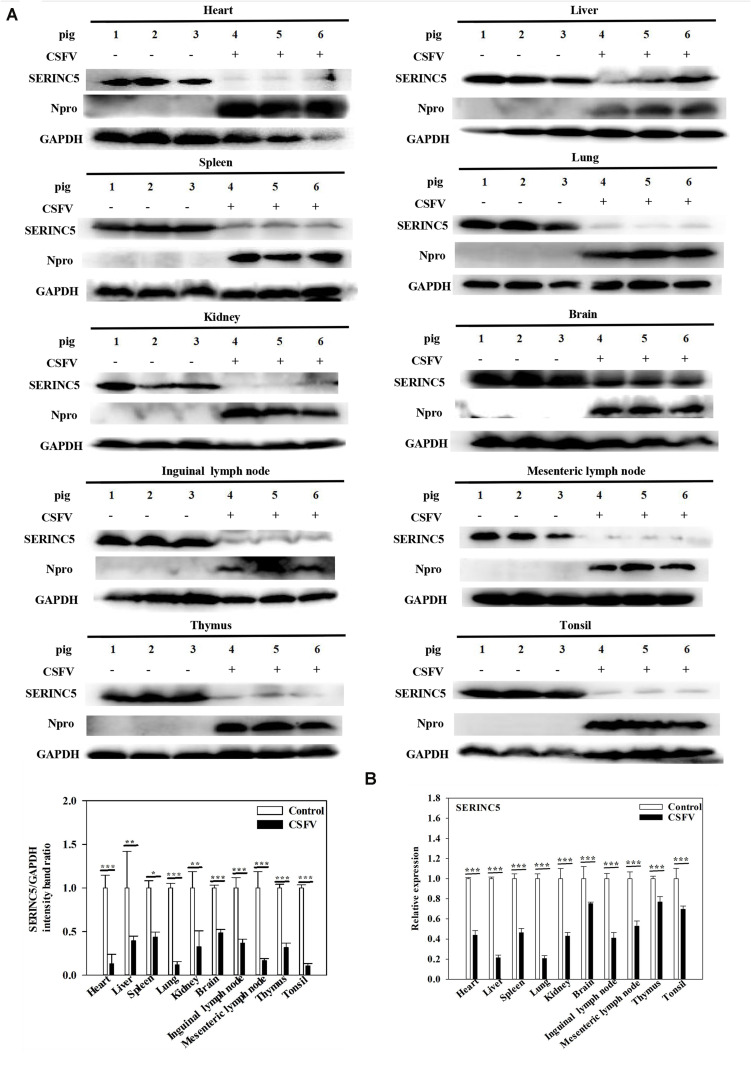
Classical swine fever virus (CSFV) infection downregulates SERINC5 expression in tissues of CSFV-infected pigs. Tissue samples (heart, liver, spleen, lung, kidney, brain, inguinal lymph nodes, mesenteric lymph nodes, thymus, and tonsil) were collected after the pigs were euthanized at 7 dpi. Protein **(A)** and transcription **(B)** of SERINC5 in different tissues were analyzed by western blotting and qRT-PCR, respectively. The relative levels of the targeted proteins were analyzed with ImageJ software, and the ratios were calculated relative to the GAPDH control. Error bars represent the mean ± SD; *n* = 3; ^∗^*P* < 0.05; ^∗∗^*P* < 0.01; ^∗∗∗^*P* < 0.001; ^NS^*P* > 0.05.

While it was demonstrated that SERINC5 inhibits CSFV replication, the next logical question would be what effect does CSFV infection have of the expression of SERINC5? To answer this, PK-15 and 3D4/2 cells were infected with CSFV (MOI of 0.1). Gene and protein expression of SERINC5 were analyzed by western blotting and qRT-PCR. The protein and gene expression of SERINC5 were markedly decreased in PK-15 (Figures 3A,C) and 3D4/2 cells (Figures 3B,D) following CSFV infection at an MOIs of 0.1 at 12, 24, 36, and 48 hpi. The protein and gene expression of SERINC5 were significantly down-regulated in PK-15 (Figures 3E,G) and 3D4/2 cells (Figures 3F,H) after infection with CSFV at MOIs of 0.1 and 1 at 24 hpi. Furthermore, SERINC5 protein and gene expression were down-regulated in tissues, including heart, liver, spleen, lung, kidney, brain, inguinal lymph nodes, mesenteric lymph nodes, thymus, and tonsil from CSFV-infected pigs (Figures 4A,B). These data suggest that CSFV can suppress the expression of SERINC5 both *in vitro* and *in vivo*.

### Screening of Proteins for Interactions With SERINC5

To investigate the mechanisms of the interactions between SERINC5 and CSFV, potential SERINC5-interacting host proteins were probed by IP and LC-MS/MS analysis. As shown in Supplementary Table S2, a total of 33 host proteins that associated with SERINC5 were identified in three independent IP and LC-MS/MS experiments. Interestingly, the key protein MDA5 of the type I IFN signaling pathway was associated with SERINC5.

### Binding of SERINC5 to the RNA Sensor MDA5

**FIGURE 5:**
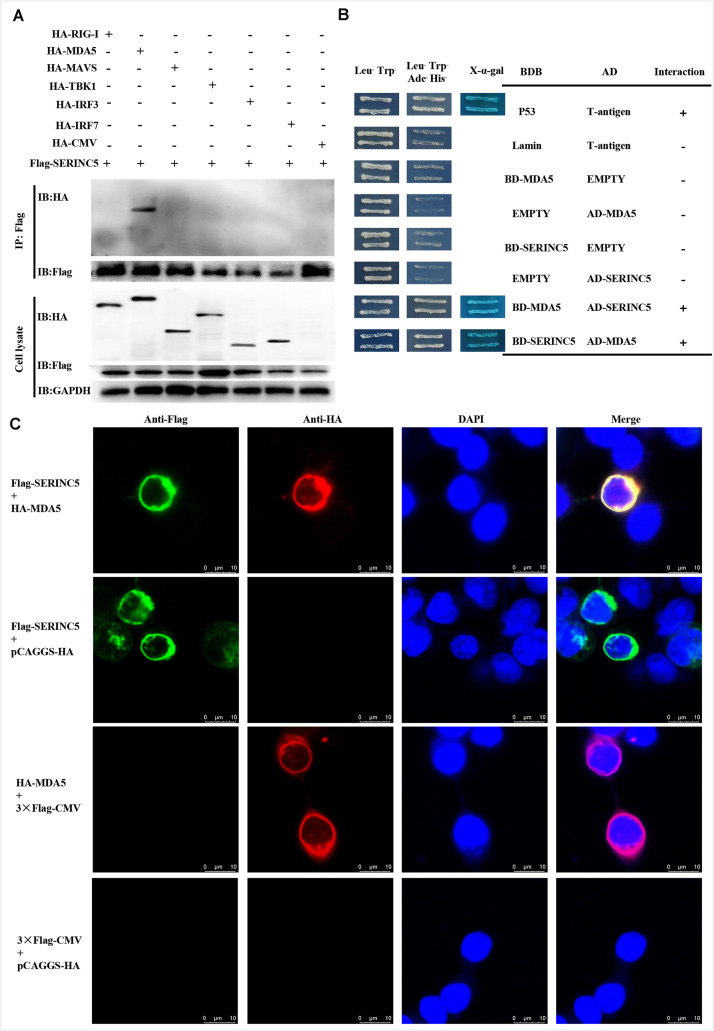
Serine incorporator 5 (SERINC5) interacted with the signaling adaptor MDA5. **(A)** Co-IP assay revealed the interactions of SERINC5 with MDA5 in HEK-293T cells. Cells were transfected with p3 × Flag-SERINC5 and HA-labeled proteins of the type I IFN signaling pathway (HA-RIG-I, HA-MDA5, HA-MAVS, HA-TBK1, HA-IRF3, and HA-IRF7). The cell lysates were harvested and immunoprecipitated with anti-Flag mAb, and analyzed by western blotting with anti-HA and anti-Flag mAb. **(B)** Y2H analysis of the interactions between SERINC5 and MDA5. The fusion plasmids of AD-SERINC5/BD-MDA5 or AD-MDA5/BD-SERINC5 were transfected into yeast strain Y2H Gold. The vectors of BD-Lamin/AD-T and BD-p53/AD-T were introduced into yeast strain Y2H Gold as negative and positive controls, respectively. **(C)** Confocal microscopy analysis of interactions of SERINC5 with MDA5 in PK-15 cells. PK-15 cells were transfected with p3 × Flag-SERINC5 and HA-MDA5 for 24 h. The cells were incubated with anti-Flag mAb and anti-HA mAb and detected by appropriate secondary antibodies followed by observation on a confocal fluorescence microscope. In the merged images, the yellow represents protein colocalization. Scale bar; 10 μm.

Melanoma differentiation-associated protein 5 is a key component of the type I IFN pathway. Potential interactions between SERINC5 and MDA5, and RIG-I, MAVS, TBK1, IRF3, and IRF7 were analyzed. Also, Co-IP assays were conducted. Cell lysates from HEK-293T cells were co-transfected to express p3 × Flag-SERINC5 and HA-labeled proteins (HA-RIG-I, HA-MDA5, HA-MAVS, HA-TBK1, HA-IRF3, and HA-IRF7). Immunoprecipitation of these proteins, p3 × Flag-SERINC5 and pCAGGS-HA, was accomplished using an anti-Flag mAb. Precipitated proteins were analyzed by western blotting with an anti-HA mAb. As presented in Figure 5A, HA-MDA5 was precipitated by Flag-SERINC5. However, other signaling molecules (RIG-I, MAVS, TBK1, IRF3, and IRF7) and the negative control pCAGGS-HA were not precipitated. The results showed that MDA5 interacts specifically with SERINC5.

To confirm the data obtained above, yeast two-hybrid (Y2H) assays were conducted. The full-length cDNAs of SERINC5 and MDA5 were cloned into the GAL4-BD and GAL4-AD to produce pGBKT7-SERINC5 and MDA5, and pGADT7-SERINC5 and MDA5, respectively. Prior to Y2H analysis, a self-activation verification of these proteins was performed. No level of self-inactivation by the proteins was observed. Next, pGBKT7-MDA5 and pGADT7-SERINC5, and pGBKT7-SERINC5 and pGADT7-MDA5 were co-transfected into Y2H cells and cultivated on synthetic media. As shown in Figure 5B, yeast cells co-transfected with pGBKT7-MDA5 and pGADT7-SERINC5, and pGBKT7-SERINC5 and pGADT7-MDA5 grew well on Leu^–^/Trp^–^/Ade^–^/His^–^ media. Furthermore, the cells formed blue colonies when cultured on X-α-gal medium. These data lend additional support to the hypothesis that MDA5 interacts with SERINC5.

To further investigate the interaction between SERINC5 and MDA5, co-localization was determined by confocal microscopy. Colocalization of SERINC5 and MDA5 was observed in PK-15 cells (Figure 5C). Together, these data suggest that SERINC5 interacts with MDA5.

### Enhancement of the Type I IFN Response by SERINC5

Melanoma differentiation-associated protein 5 is a critical mediator of the I IFN response. Interestingly, the data provided above showed that SERINC5 not only exhibits anti-CSFV activity, but interacts with MDA5 as well. Thus, these results inspired us to explore the possible role of SERINC5 in type I IFN signaling induced by viral infection.

**FIGURE 6:**
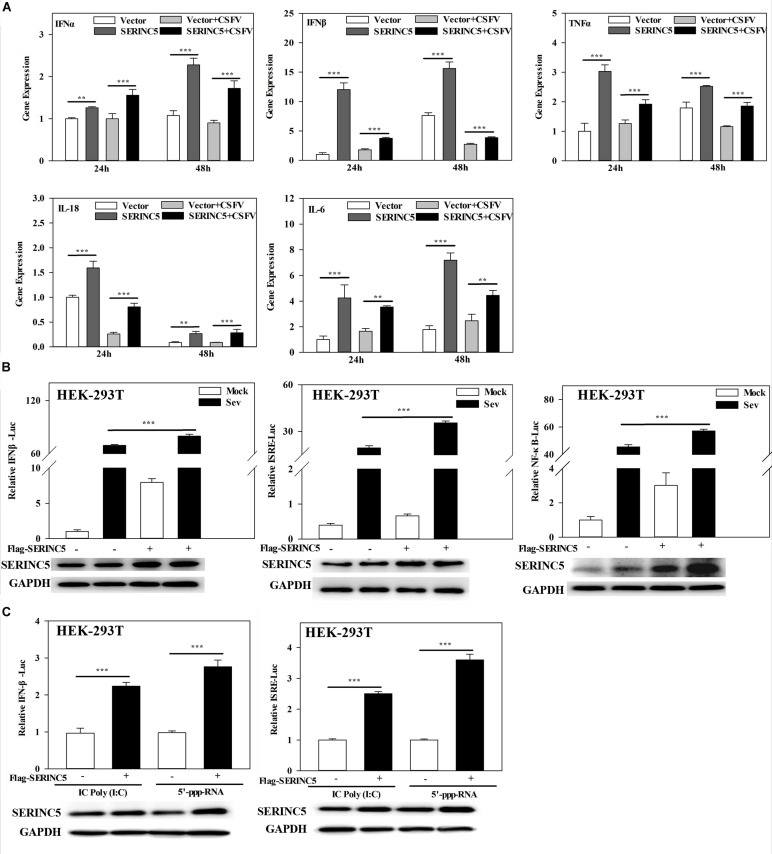
Serine incorporator 5 (SERINC5) positively regulates the type I IFN signaling pathway. **(A)** PK-15 cells were transfected with p3 × Flag-SERINC5 followed by infection with CSFV (MOI = 0.1) for 24 and 48 h. The expression of *IFN*α, *IFN*β, *TNF*α, *IL-18*, and *IL-6* mRNA was determined by qRT-PCR. **(B)** HEK-293T cells were transfected with p3 × Flag-SERINC5 together with the IFNβ, ISRE or NF-κB luciferase reporter and pTK-Rluc for 24 h. The cells were then infected with SeV for another 24 h. Luciferase activities were detected via a dual-luciferase reporter assay. pTK-Rluc was used as an internal control. **(C)** HEK-293T cells were transfected with p3 × Flag-SERINC5 along with the IFNβ or ISRE luciferase reporter and pTK-Rluc. Luciferase activities were detected after incubation with poly (I:C) (10 mg/mL) or 5′-ppp-RNA (2 mg/mL) for 24 h. pTK-Rluc was used as an internal control. The protein expression of p3 × Flag-SERINC5 in HEK-293T cells was assayed by western blotting. GAPDH was used as a loading control. Error bars represent the mean ± SD; *n* = 3; ^∗^*P* < 0.05; ^∗∗^*P* < 0.01; ^∗∗∗^*P* < 0.001; ^NS^*P* > 0.05.

To clarify the molecular mechanism of SERINC5s role in the I IFN pathway, mRNA expression of *IFN*α, *IFN*β, *TNF*α, *IL-18*, and *IL-6* in CSFV- infected PK-15 cells transfected with or without p3 × Flag-SERINC5 were measured. As shown in Figure 6A, the mRNA expression of the genes analyzed was markedly increased in SERINC5 overexpressing cells. These observations indicate that SERINC5 expression may influence the type I IFN pathway. To verify the preliminary data, luciferase reporter assays were performed. The promoter activities of IFNβ, ISRE, and NF-κB increased in SeV-infected cells were transfected with SERINC5, suggesting that SERINC5 promoted type I IFN activation in response to infection with an RNA virus (Figure 6B). Next, the luciferase reporter assay of promoter activity of IFNβ and ISRE was conducted to investigate the role of SERINC5 in the context of poly (I:C) and 5′-ppp-RNA (models of RIG-I activation). The promoter activities were significantly induced by SERINC5 overexpression, indicating that RLR-mediated type I IFN signaling was up-regulated by SERINC5 (Figure 6C). Overall, these results suggest that the type I IFN pathway is positively regulated by SERINC5.

### Regulation of MDA5-Mediated Activation of Type I IFN Pathway by SERINC5

**FIGURE 7:**
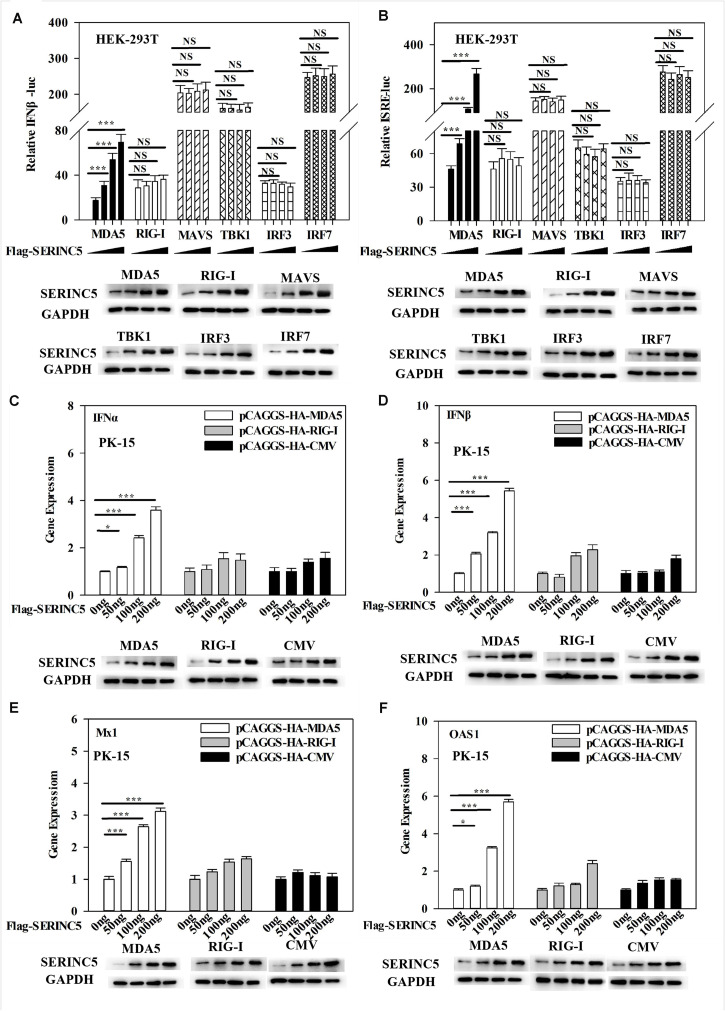
Melanoma differentiation-associated protein 5 (MDA5) activation of the type I IFN signaling pathway is enhanced by SERINC5. HEK-293T cells were co-transfected with p3 × Fag-SERINC5 (0, 50, 100, and 200 ng), HA- RIG-I, MDA5, MAVS, IRF3, and IRF7. Luciferase activities of IFNβ **(A)** and ISRE **(B)** were detected by a dual-luciferase reporter assay after transfection with the IFNβ or ISRE luciferase reporter and pTK-Rluc for 24 h. pTK-Rluc was used as an internal control. PK-15 cells were co-transfected with p3 × Flag-SERINC5 (0, 50, 100, and 200 ng) together with HA-MDA5 (200 ng), HA-RIG-I (200 ng), or HA-CMV (200 ng) for 24 h. The gene expression of *IFN*α **(C)**, *IFN*β **(D)**, *Mx1* **(E)**, and *OAS1* **(F)** were analyzed by qRT-PCR. Error bars represent the mean ± SD; *n* = 3; ^∗^*P* < 0.05; ^∗∗^*P* < 0.01; ^∗∗∗^*P* < 0.001; ^NS^*P* > 0.05.

The above data indicated that SERINC5 interacted with MDA5 and activated the type I IFN pathway. Thus, it was hypothesized that the MDA5-mediated activation of the IFN-α/β pathway could be regulated by SERINC5. To test this hypothesis, luciferase reporter assays of IFNβ and ISRE promoter activities were performed. It was observed that SERINC5 specifically enhanced MDA5-mediated type I IFN activation, but not that initiated by RIG-I, MAVS, TBK1, IRF3 or IRF7 (Figures 7A,B). Furthermore, mRNA levels of *IFN*α, *IFN*β, *Mx1*, and *OAS1* were measured. In PK-15 cells were co-transfected with plasmids encoding SERINC5 and MDA5 rather than RIG-I significantly increased mRNA expression of *IFN*α, *IFN*β, *Mx1*, and *OAS1* in a dose-dependent manner (Figures 7C–F). Taken together, these results suggest that the MDA5-mediated type I IFN pathway is likely regulated by SERINC5.

### Reduction of Antiviral Activity of SERINC5 in MDA5 Deficient Cells

**FIGURE 8:**
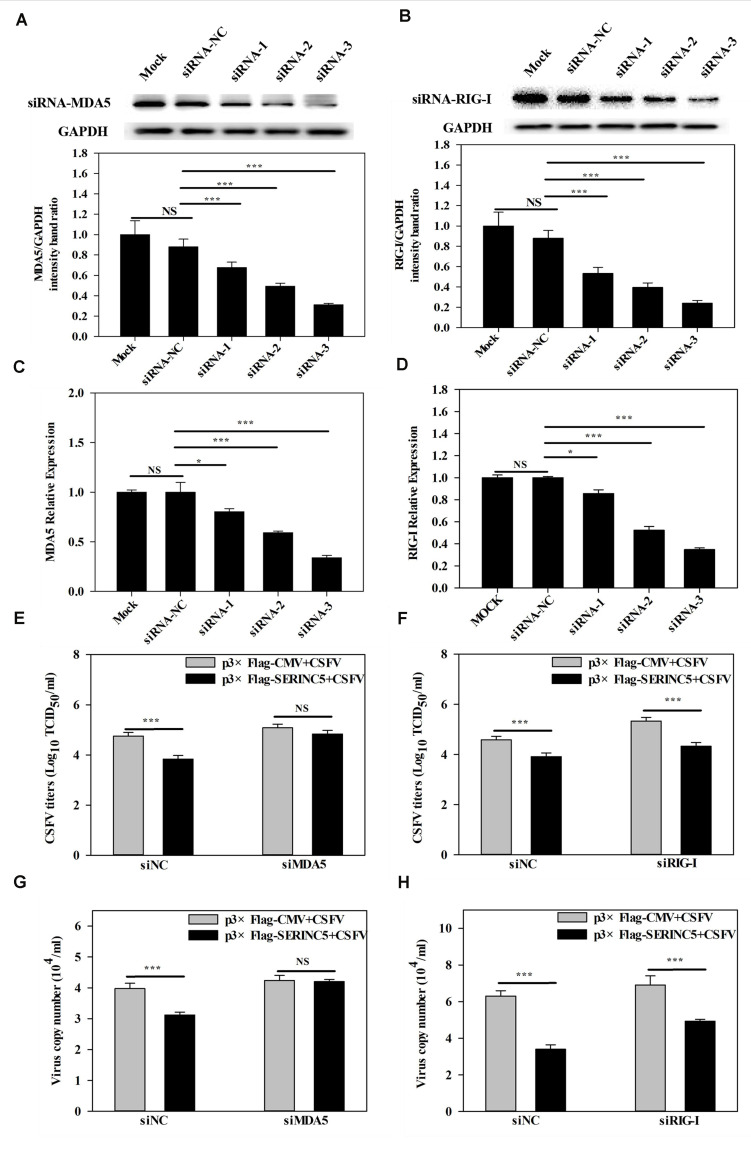
Inhibition of MDA5 expression reduced the antiviral activity of SERINC5. The siRNA silencing efficiency of MDA5 **(A,C)** and RIG-I **(B,D)** in PK-15 cells were assessed by western blotting **(A,B)** and qRT-PCR **(C,D)**. The influence of SERINC5 anti-CSFV activity after transfection with siRNAs targeted MDA5 and RIG-I siRNA. PK-15 cells were infected with CSFV (MOI = 0.1) for 24 h after co-transfection with MDA5 siRNA, siNC with p3 × Flag-SERINC5 or p3 × Flag-CMV **(E,G)**, RIG-I siRNA, siNC with p3 × Flag-SERINC5 or p3 × Flag-CMV **(F,H)**. The viral titers **(E,F)** were reported as TCID_50_, and viral genome copy numbers **(G,H)** were analyzed by qRT-PCR. The relative levels of the targeted proteins were analyzed with ImageJ software, and the ratios were calculated relative to the GAPDH control. Error bars represent the mean ± SD; *n* = 3; ^∗^*P* < 0.05; ^∗∗^*P* < 0.01; ^∗∗∗^*P* < 0.001; ^NS^*P* > 0.05.

It was demonstrated in a previous report by us that MDA5-dependent signaling pathways can be triggered by CSFV infection (Dong et al., 2013). Furthermore, the referenced data indicated that SERINC5 expression was connected with the MDA5-mediated Type I IFN signaling pathway. Therefore, it was hypothesized that the MDA5-mediated Type I IFN signaling pathway might involved in the anti-CSFV activity of SERINC5. To verify this, RNA interference experiments were used to silence endogenous MDA5 and RIG-I expression in PK-15 cells. As shown in Figures 8A–D, the silencing efficiency of MDA5 and RIG-I was verified by western blotting and qRT-PCR. Next, the viral genomes and infectious titers of CSFV were measured. It was observed that CSFV replication was significantly decreased in PK-15 cells after co-transfection with plasmids encoding SERINC5 and siNC or RIG-I siRNA (Figures 8F,H). However, when MDA5 expression was knocked down, CSFV replication remained at control levels in PK-15 cells transfected with SERINC5 (Figures 8E,G). The above results indicate that MDA5 is an important factor through which SERINC5 exerts its anti-CSFV activity.

## Discussion

It is well-known that the body has several antiviral factors that interfere with different stages of the viral life cycle (Evans and Liu, 2020). However, viruses have evolved various mechanisms to counteract the host antiviral factors (Malim and Emerman, 2008; Sauter and Kirchhoff, 2018). SERINC5 is a novel host restriction factor that defends against viral infections, including that of murine leukemia virus (MLV), human immune deficiency virus (HIV) and equine infectious anemia virus (EIAV) (Ahi et al., 2016; Chande et al., 2016; Trautz et al., 2016). A series of studies have been conducted to elucidate the potential role of SERINC5 as an antiviral effector (Oliver, 2015). It has been reported that SERINC5 and SERINC3 are important restriction factors early in the life cycle of the virus, which is not only limited to retroviruses, but also applies to other viruses and pathogens (Ahi et al., 2016). In the current study, the effect of SERINC5 on CSFV replication was examined. The data indicated that viral genome replication and viral titers were significantly reduced in PK-15 and 3D4/2 cells after overexpression of SERINC5 (Figure 1). Furthermore, CSFV replication was apparently enhanced in cells with SERINC5 expression knocked down (Figure 2). The results suggest that SERINC5 is an anti-CSFV protein. SERINC proteins belong to the transmembrane protein family and exist in all eukaryotic cells. Since the SERINC proteins are highly conserved among eukaryotic species, it is possible that they serve critical functions in the cell membrane (Inuzuka et al., 2005; Bossolasco et al., 2006). As a transmembrane protein, SERINC5 inhibits retroviruses and other enveloped viruses (Firrito et al., 2018). In addition, other studies have indicated that viral infections also influence restriction factors as well. For instance, the sensitivity of the virus particle to SERINC5 was altered by mutations in the viral envelope glycoprotein (Usami et al., 2015; Ziglio et al., 2015; Ahi et al., 2016; Chande et al., 2016; Beitari et al., 2017). It has also been reported that the viral proteins antagonize SERINC5 by altering its subcellular localization and preventing its insertion into viral particles (Chande et al., 2016). In the present study, it was observed that SERINC5 expression was reduced by CSFV infection both *in vitro* and *in vivo* (Figures 3, 4), which supports the hypothesis that SERINC5 may play a key role in the host defense against CSFV infection. Conversely, SERINC5 could be packaged into the viral envelope, which resulted in a more than 100-fold reduction in infectivity (Usami et al., 2015; Ziglio et al., 2015). However, the specific molecular mechanism through which SERINC5 exerts its antiviral effects against CSFV and the specific stage of replication that this inhibition remains elusive.

Next, the molecular mechanisms by which SERINC5 inhibits CSFV replication was probed. A total of 33 swine cellular proteins, which potentially interact with SERINC5 were screened using IP-coupled LC-MS/MS (Supplementary Table S2). Among the 33 identified host proteins, only the interaction between SERINC5 and MDA5 was verified using Co-IP, yeast two-hybrid and subcellular co-localization (Figure 5). Both MDA5 and RIG-I are known as RLRs (Pang and Iwasaki, 2011). On the one hand, RIG-1 and MDA5 can induce the expression of IFNs and inflammatory factors by triggering the activation of RLRs and downstream signaling molecules (Goraya et al., 2018). On the other hand, RIG-1 and MDA5 can act as dsRNA receptors to recognize CSFV infection, resulting in stable expression of type I interferon (Chen et al., 2015). Therefore, it was hypothesized that SERINC5 might be a component of the type I interferon signaling pathway. Our data indicate that SERINC5 positively regulates RNA virus-induced type I interferon signaling. Furthermore, SERINC5 enhanced MDA5 mediation, but not RIG-I-mediated type I interferon induction (Figures 6, 7). Many proteins exerted anti-viral effects do so through interactions with MDA5. For example, ribonucleoprotein PTB-binding 1 (RAVER1) acts as a coactivator of MDA5-mediated signaling through direct interactions (He et al., 2013). The ability of MDA5-mediated innate antiviral immunity was enhanced by TRIM65, which interacts with MDA5 (Lang et al., 2016). Similarly, the porcine 2′-5′-oligoadenylate synthase-like protein (pOASL) interacts with MDA5 to enhance the MDA5-mediated type I IFN signaling to suppresses CSFV replication (Li et al., 2017). However, it was unclear whether SERINC5 acted in a similar fashion, in which RAVER1, TRIM65, or pOASL exerted anti-CSFV effects through the same signaling pathway. Therefore, RNAi was used to suppress MDA5 after CSFV infection. It was observed that the inhibition of MDA5 eliminated the antiviral effect of SERINC5 on CSFV replication. These data indicate that SERINC5 exerts anti-CSFV through the MDA5 signaling pathway (Figure 8).

In summary, the data present compelling evidence that SERINC5 is a novel anti-CSFV protein that acts by promoting MDA5-mediated type I interferon signaling.

## Data Availability Statement

The datasets presented in this study can be found in online repositories. The names of the repository/repositories and accession number(s) can be found at: https://www.ncbi.nlm.nih.gov/genbank/, XM_013994946.2 https://www. ncbi.nlm.nih.gov/genbank/, MF358966.1 https://www.ncbi.nlm.nih.gov/genbank/, MF358967.1 https://www.ncbi.nlm.nih.gov/genbank/, MK302496.1 https://www.ncbi.nlm.nih.gov/genbank/, EU091339.1 https://www.ncbi.nlm.nih.gov/genbank/, NM_213 770.1 https://www.ncbi.nlm.nih.gov/genbank/, NM_0010974 28.1 https://www.ncbi.nlm.nih.gov/genbank/, NM_001206359.1 https://www.ncbi.nlm.nih.gov/genbank/, NC_002657.1 https://www.ncbi.nlm.nih.gov/genbank/, JQ839262.1 https://www.ncbi.nlm.nih.gov/genbank/, GQ415073.1 https://www.ncbi.nlm.nih. gov/genbank/, AF518322.1 https://www.ncbi.nlm.nih.gov/genbank/, AF191088.1 https://www.ncbi.nlm.nih.gov/genbank/, DQ095779.1 https://www.ncbi.nlm.nih.gov/genbank/, NM_001031790.1.

## Ethics Statement

The animal study was reviewed and approved by The Ethics Committee and the Laboratory Animal Care and Use Committee of South China Agricultural University.

## Author Contributions

WL, JC, and MZ conceived and designed the experiment and carried out the analysis. WL, ZZ, and LZ performed the experiments. WL, HL, SF, EZ, JF, ZL, WC, LY, and HD assisted with animal experiment. WL wrote the manuscript. All authors read and approved the final manuscript.

## Conflict of Interest

HL was employed by Shandong Qianxi Agriculture & Animal Husbandry Development Co., Ltd. The remaining authors declare that the research was conducted in the absence of any commercial or financial relationships that could be construed as a potential conflict of interest.

## Supplementary Material

TABLE S1 Primers and siRNA used in this study.

TABLE S2 Serine incorporator 5 interacting proteins selected by LC-MS/MS in PK-15 cells.
