# Supplementary material for: Antiviral Role of Serine Incorporator 5 (SERINC5) Proteins in Classical Swine Fever Virus Infection
Source: Front Microbiol. 2020 Sep 4;11:580233. doi: 10.3389/fmicb.2020.580233 (PMC7498654; doi:10.3389/fmicb.2020.580233)
Supplement: TABLE S1 — Primers and siRNA used in this study. [file Table_1.DOC]

**TABLE S1 |** Primers and siRNA used in this study.

| **Primer** | **Forward primer (5’-3’)** | **Experiment** |
| --- | --- | --- |
| Flag-SERINC5-F | CTTGCGGCCGCGAATTCAATGTCAGCGCAGTGCTGTGC | Gene clone |
| Flag-SERINC5-R | TCTAGAGTCGACTGGTACCGATCACACGGAGAACTGGGGAG |  |
| EGFP-SERINC5-F | GCTCAAGCTTCGAATTCTATGTCAGCGCAGTGCTGTGC | Gene clone |
| EGFP-SERINC5-R | TCCCGGGCCCGCGGTACCTCACACGGAGAACTGGGGAG |  |
| HA-RIG-I-F | GTTCCAGATTACGCTGAATTCATGACAGCAGAGCAGCGG | Gene clone |
| HA-RIG-I-R | TCGAGGCATGCCCGGGTACCCTAGCTGGTGATGGGCTCAA |  |
| HA-MDA5-F | GTTCCAGATTACGCTGAATTCATGTCGTCGGATGGGTATTC | Gene clone |
| HA-MDA5-R | TCGAGGCATGCCCGGGTACCCTAGTCCTCATCACTAGACA |  |
| HA-MAVS-F | GTTCCAGATTACGCTGAATTCATGACGTTTGCCGAGGACAAG | Gene clone |
| HA-MAVS-R | TCGAGGCATGCCCGGGTACCTCACTGGGGCAGGCGCCG |  |
| HA-TBK1-F | GTTCCAGATTACGCTGAATTCATGCAGAGCACTTCTAATCATC | Gene clone |
| HA-TBK1-R | TCGAGGCATGCCCGGGTACCCTAAAGACAGTCAACATTGC |  |
| HA-IRF3-F | GTTCCAGATTACGCTGAATTCATGGGAACTCAGAAGCCTC | Gene clone |
| HA-IRF3-R | TCGAGGCATGCCCGGGTACCCTAGAAATCCATGTCCTCCACC |  |
| HA-IRF7-F | GTTCCAGATTACGCTGAATTCATGGCCGCGGCTCCTGACAG | Gene clone |
| HA-IRF7-R | TCGAGGCATGCCCGGGTACCCTAGGCCGGCTGCTCCACCT |  |
| GAPDH-F | TGGAGTCCACTGGTGTCTTCAC | RT-qPCR |
| GAPDH-R | TTCACGCCCATCACAAACA |  |
| CSFV-NS5B-F | CCTGAGGACCAAACACATGTTG | RT-qPCR |
| CSFV-NS5B-R | TGGTGGAAGTTGGTTGTGTCTG |  |
| SERINC5-F | GAAGAGCAGCAGAATGTGAAAG | RT-qPCR |
| SERINC5-R | CATAGAGGGAAGCCAAGAAGAA |  |
| IFN-α-F | CATCCTGGCTGTGAGGAAATA | RT-qPCR |
| IFN-α-R | CAGGTTTCTGGAGGAAGAGAAG |  |
| IFN-β-F | AGCAGATCTTCGGCATTCTC | RT-qPCR |
| IFN-β-R | GTCATCCATCTGCCCATCAA |  |
| IL-6-F | GGAGACCTGCTTGATGAGAATC | RT-qPCR |
| IL-6-R | CAGCCTCGACATTTCCCTTAT |  |
| IL-18-F | TAATGCACCTCAGACCGTATTT | RT-qPCR |
| IL-18-R | CACTGCACAGAGATGGTTACT |  |
| Mx1-F | GAACGAAGAAGACGAATGGAAGG | RT-qPCR |
| Mx1-R | GATGCCAGGAAGGTCTATGAGG |  |
| OAS1-F | ACCTGAAGTACGTGAAAGCCA | RT-qPCR |
| OAS1-R | ACGAGGCCTCTGTCCAAATG |  |
| pGBKT7-SERINC5-F | ATGGCCATGGAGGCCGAATTCATGatgtcagcgcagtgctgtgc | Y2H |
| pGBKT7-SERINC5-R | TGCGGCCGCTGCAGGTCGACGtcacacggagaactggggag |  |
| pGBKT7-MDA5-F | ATGGCCATGGAGGCCGAATTCatgtcgtcggatgggtattc | Y2H |
| pGBKT7-MDA5-R | TGCGGCCGCTGCAGGTCGACGctagtcctcatcactagaca |  |
| pGADT7-SERINC5-F | ATGGCCATGGAGGCCAGTGAATTCatgtcagcgcagtgctgtgc | Y2H |
| pGADT7-SERINC5-R | TGCAGCTCGAGCTCGATGGATCCCtcacacggagaactggggag |  |
| pGADT7-MDA5-F | ATGGCCATGGAGGCCAGTGAATTCatgtcgtcggatgggtattc | Y2H |
| pGADT7-MDA5-R | TGCAGCTCGAGCTCGATGGATCCCctagtcctcatcactagaca | Y2H |
| siRNA SERINC5-679 | Sense (5’-3’): CCUCUUGAUUGUGGAGUUUTT | siRNA |
|  | Antisense (5’-3’): AAACUCCACAAUCAAGAGGTT |  |
| siRNA SERINC5-1056 | Sense (5’-3’): CCAUCUGUGUGCCUGACUUTT | siRNA |
|  | Antisense (5’-3’): AAGUCAGGCACACAGAUGGTT |  |
| siRNA SERINC5-1479 | Sense (5’-3’): GCUGGAUAUGCGUGCUCUUTT | siRNA |
|  | Antisense (5’-3’): AAGAGCACGCAUAUCCAGCTT |  |
| siRNA MDA5-1441 | Sense (5’-3’): CCUCAGAUAUUGGGACUAATT | siRNA |
|  | Antisense (5’-3’): UUAGUCCCAAUAUCUGAGGTT |  |
| siRNA MDA5-1848 | Sense (5’-3’): CCGAAGGAUUGAUGCCUAUTT | siRNA |
|  | Antisense (5’-3’): AUAGGCAUCAAUCCUUCGGTT |  |
| siRNA MDA5-2195 | Sense (5’-3’): CCCAGUGGAUUACUGACAATT | siRNA |
|  | Antisense (5’-3’): UUGUCAGUAAUCCACUGGGTT |  |
| siRNA RIG-1-946 | Sense (5’-3’): GGUACAAAGUUGCAGGCATT | siRNA |
|  | Antisense (5’-3’): UGCCUGCAACUUUGUACCTT |  |
| siRNA RIG-1-1126 | Sense (5’-3’): GCAAACAGCAUCCUUAUAATT | siRNA |
|  | Antisense (5’-3’): UUAUAAGGAUGCUGUUUGCTT |  |
| siRNA RIG-1-1477 | Sense (5’-3’): CCAUAACUCUUGGAGGCUUTT | siRNA |
|  | Antisense (5’-3’): AAGCCUCCAAGAGUUAUGGTT |  |
| siNC | Sense (5’-3’): UUCUCCGAACGUGUCACGUTT | siRNA |
|  | Antisense (5’-3’): ACGUGACACGUUCGGAGAATT |  |

**TABLE S2 |** SERINC5 interacting proteins selected by LC-MS/MS in PK-15 cells.

| Number | GenBank accession | Gene symbol | Protein name |
| --- | --- | --- | --- |
| 1 | NM_001038694.1 | ACSL4 | Acyl-CoA synthetase long-chain family member 4 |
| 2 | XM_003127976.3 | CLIC1 | Chloride intracellular channel protein 1 |
| 3 | NM_001105301.1 | COPS2 | Constitutive photomophogenesis protein |
| 4 | XM_021089027.1 | eIF3e | Eukaryotic translation initiation factor 3 subunit E |
| 5 | XM_021062272.1 | eIF3f | Eukaryotic translation initiation factor 3 subunit F |
| 6 | HQ026021.1 | IFN-γ | Interferon gamma |
| 7 | XM_003124230.2 | IFITM1 | Interferon-induced transmembrane protein 1 |
| 8 | GU295944.1 | IRF2 | Interferon regulatory factor 2-binding protein |
| 9 | XM_021067458.1 | KIAA0368 | Proteasome-associated protein ECM29-like protein |
| 10 | NM_002265.6 | KPNB1 | Importin subunit beta-1 |
| 11 | NM_001128435.1 | LBP | Laminin binding protein |
| 12 | XM_001925826.6 | MCU | [Mitochondrial calcium uniporter](https://www.ncbi.nlm.nih.gov/nuccore/XM_001925826.6) |
| 13 | MF358967.1 | MDA5 | Melanoma differentiation-associated gene-5 |
| 14 | NM_001285976.1 | NAP1L4 | Nucleosome assembly protein 1-like 4 |
| 15 | XM_003358764.5 | NCBP2 | Nuclear cap-binding protein subunit 2 |
| 16 | NM_001204759.1 | NME1 | Nucleoside diphosphate kinase 1 |
| 17 | NM_001244416.1 | PFN-1 | Profilin-1 |
| 18 | NM_001099932.2 | PGK1 | Phosphoglycerate kinase |
| 19 | XM_003122384.5 | POLR2L | [Polymerase II subunit L](https://www.ncbi.nlm.nih.gov/nuccore/XM_003122384.5) |
| 20 | NM_001244474.1 | PRDX2 | Peroxiredoxin-2 |
| 21 | NM_006793.5 | PRDX3 | Homo sapiens peroxiredoxin 3 |
| 22 | NM_001123173.1 | Rab-1B | Ras-related protein Rab-1B |
| 23 | NM_001243060.1 | Rab-10 | Ras-related protein Rab-10 |
| 24 | XM_021094700.1 | RPL18 | Ribosomal protein L18 |
| 25 | XM_021066688.1 | RPL38 | Ribosomal protein L38 |
| 26 | AY550063.1 | SIAHBP1 | Fuse-binding protein-interacting repressor |
| 27 | NM_001293285.2 | SPATS2 | Spermatogenesis-associated serine-rich protein 2 |
| 28 | XM_021071128.1 | SURF4 | Surfeit 4 |
| 29 | NM_001130211.1 | TFAM | Transcription factor A, mitochondrial |
| 30 | XM_021071309.1 | TOM70 | 70 kDa mitochondrial outer membrane protein |
| 31 | XM_021097344.1 | TRIM28 | Tripartite motif containing 28 |
| 32 | AY148222.1 | UbcH5B | [E2 ubiquitin conjugating enzyme](https://www.baidu.com/link?url=wHqhoNvqgkhHMuwCeviXbFvkj9bKPbPHQCKg9zl9rBV1qBUfZywVr-SX0CX1-uaG&wd=&eqid=f270db1b0011d6f1000000065ec87fca) |
| 33 | XM_021064658.1 | [UBQLN1](https://www.ncbi.nlm.nih.gov/nuccore/XM_021064658.1) | [Ubiquilin 1](https://www.ncbi.nlm.nih.gov/nuccore/XM_021064658.1) |
