# Supplementary figures and images for: Antiviral Role of Serine Incorporator 5 (SERINC5) Proteins in Classical Swine Fever Virus Infection (part 2 of 2)
Source: Front Microbiol. 2020 Sep 4;11:580233. doi: 10.3389/fmicb.2020.580233 (PMC7498654; doi:10.3389/fmicb.2020.580233)

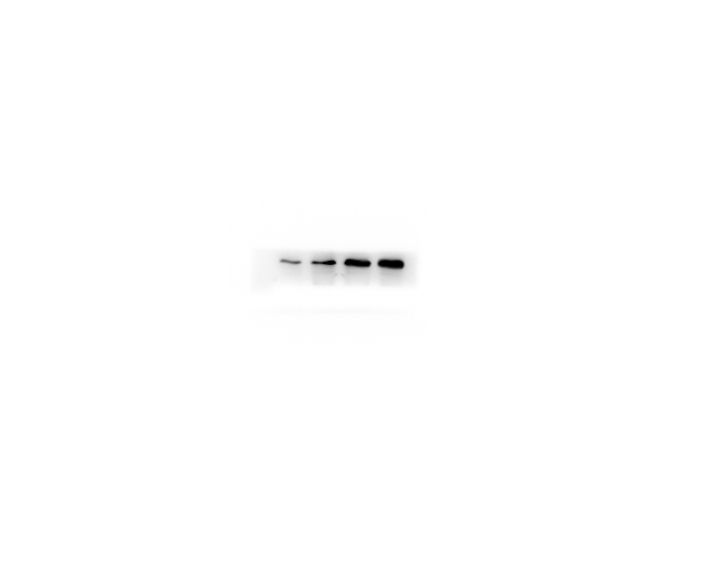

Supplement: Supplementary file 2 [file Data_Sheet_1.zip › supplementary material/Figure7D-SERINC5-CMV.tif]

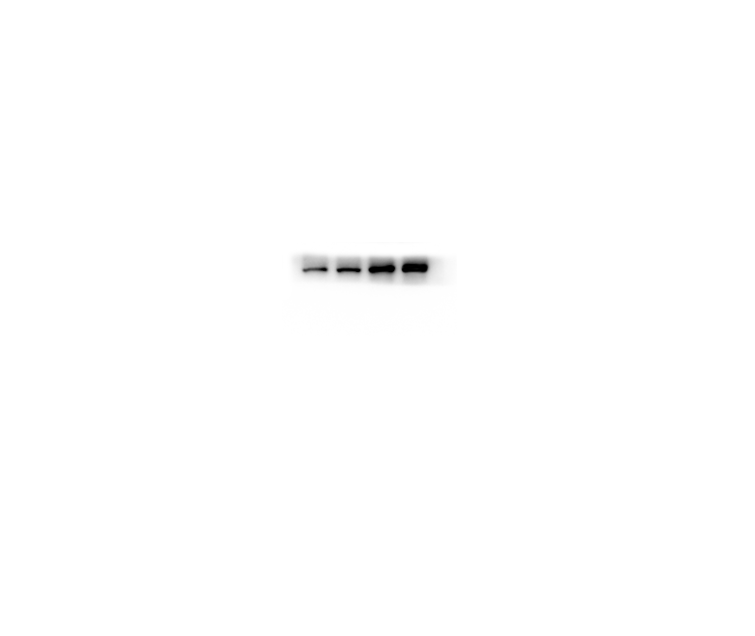

Supplement: Supplementary file 2 [file Data_Sheet_1.zip › supplementary material/Figure7D-SERINC5-MDA5.tif]

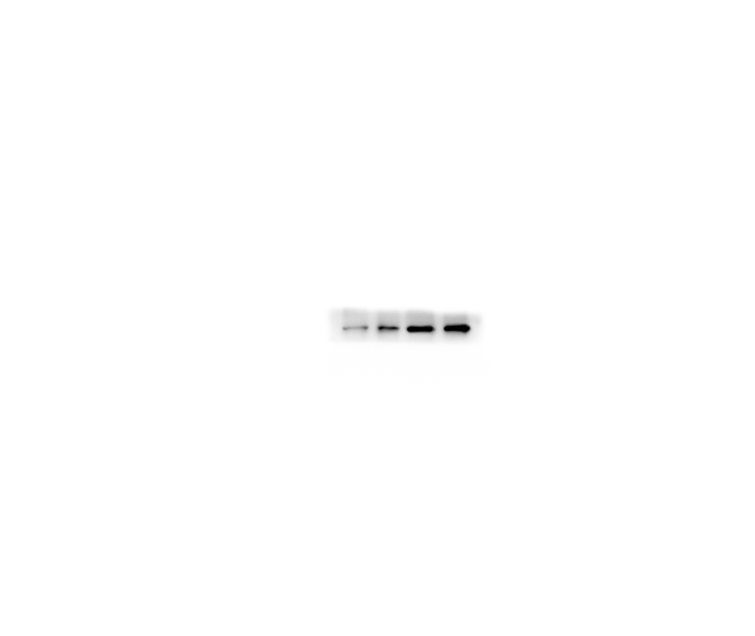

Supplement: Supplementary file 2 [file Data_Sheet_1.zip › supplementary material/Figure7D-SERINC5-RIG-I.tif]

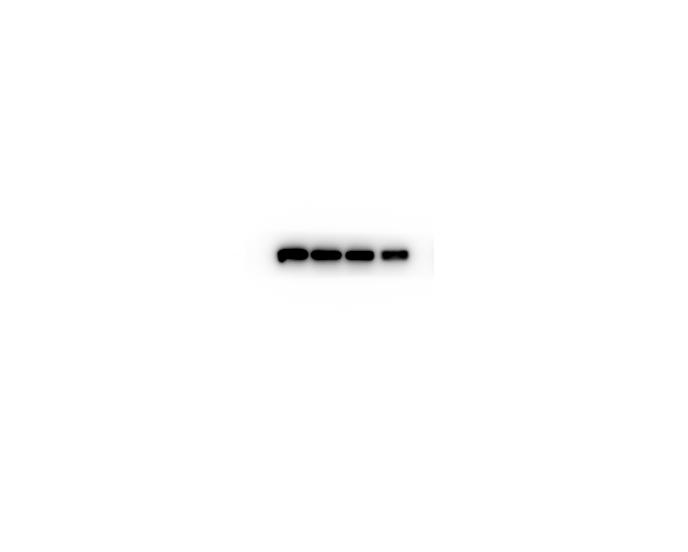

Supplement: Supplementary file 2 [file Data_Sheet_1.zip › supplementary material/Figure7E-GAPDH-CMV.tif]

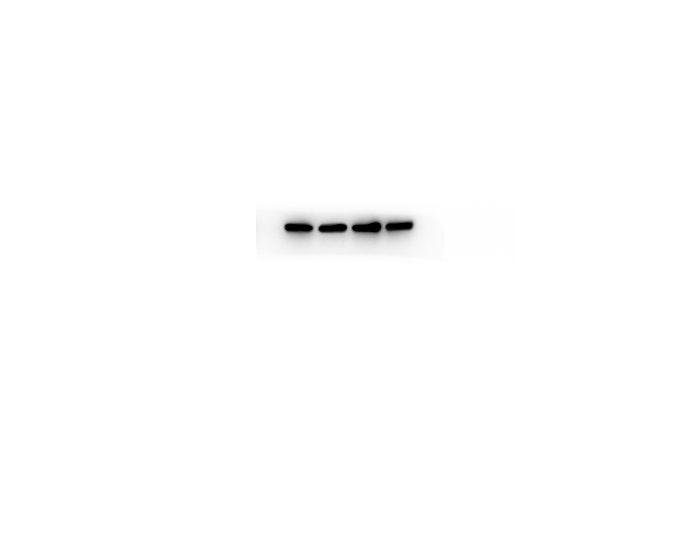

Supplement: Supplementary file 2 [file Data_Sheet_1.zip › supplementary material/Figure7E-GAPDH-MDA5.tif]

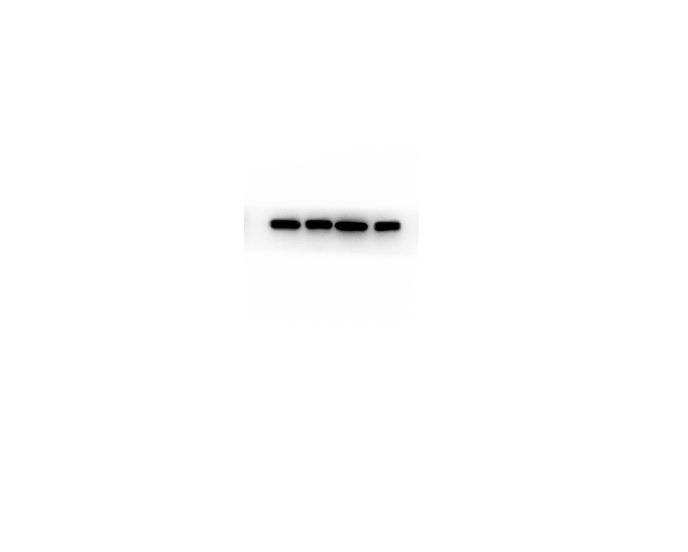

Supplement: Supplementary file 2 [file Data_Sheet_1.zip › supplementary material/Figure7E-GAPDH-RIG.tif]

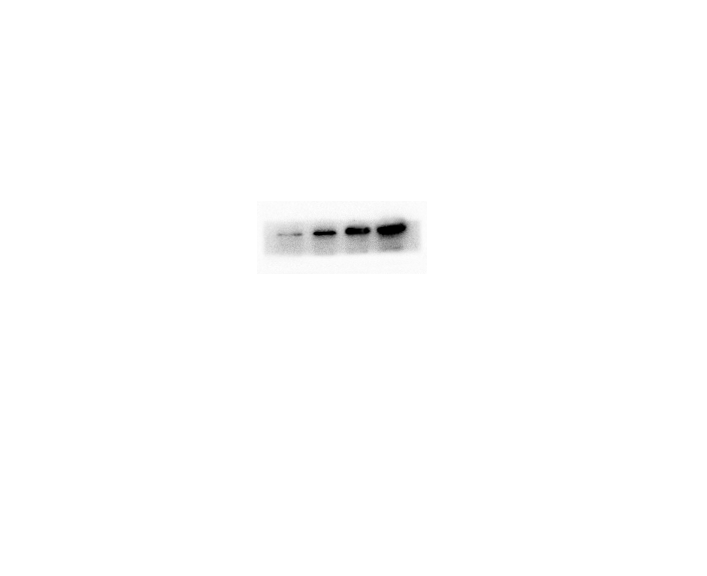

Supplement: Supplementary file 2 [file Data_Sheet_1.zip › supplementary material/Figure7E-SERINC5-CMV.tif]

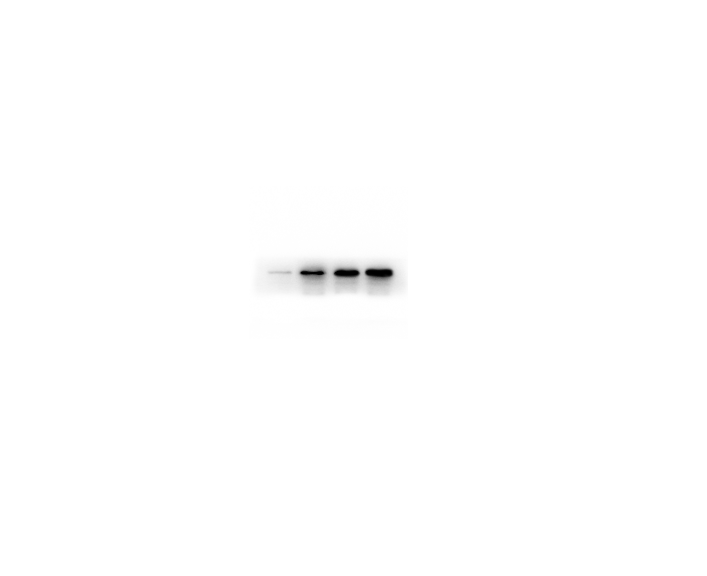

Supplement: Supplementary file 2 [file Data_Sheet_1.zip › supplementary material/Figure7E-SERINC5-MDA5.tif]

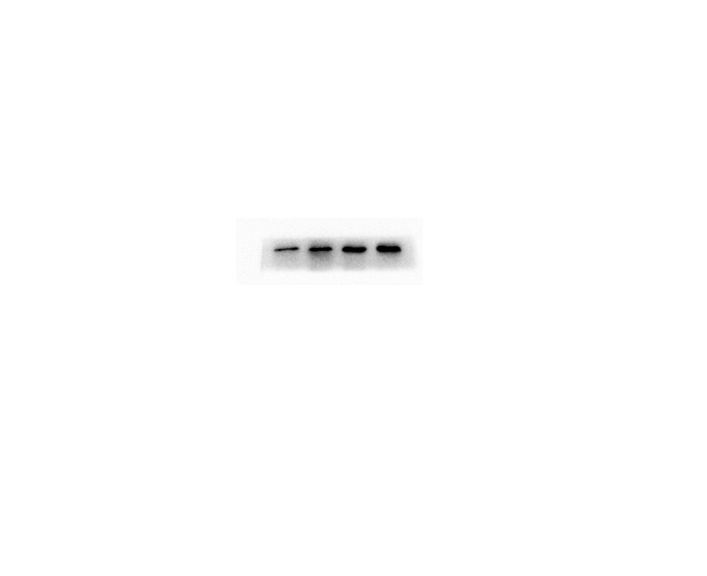

Supplement: Supplementary file 2 [file Data_Sheet_1.zip › supplementary material/Figure7E-SERINC5-RIG-I.tif]

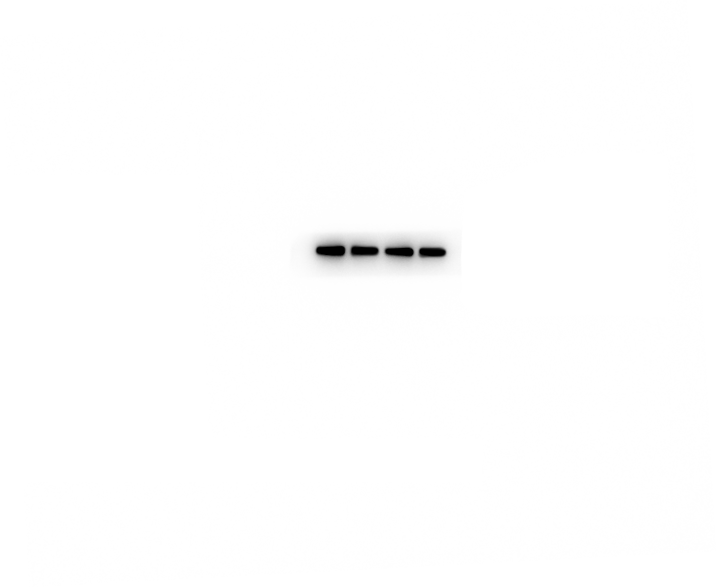

Supplement: Supplementary file 2 [file Data_Sheet_1.zip › supplementary material/Figure7F-GAPDH-CMV.tif]

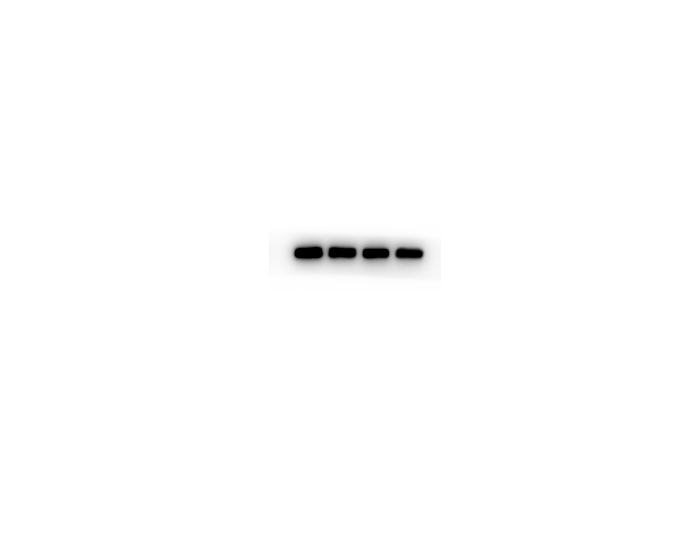

Supplement: Supplementary file 2 [file Data_Sheet_1.zip › supplementary material/Figure7F-GAPDH-MDA5.tif]

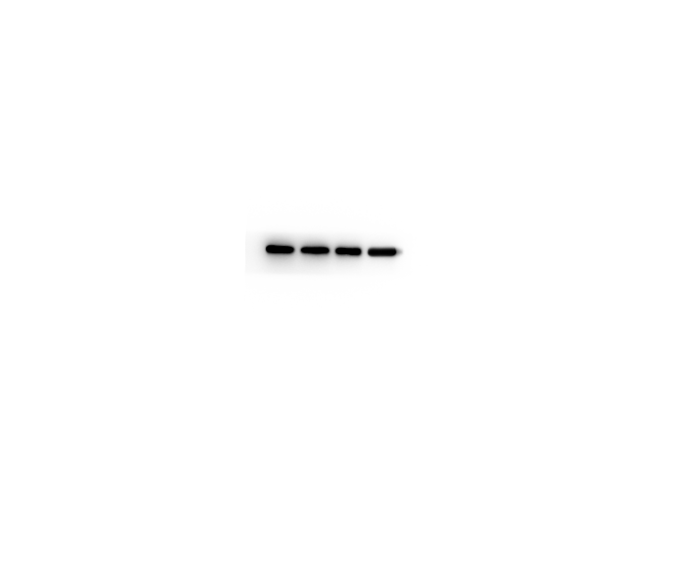

Supplement: Supplementary file 2 [file Data_Sheet_1.zip › supplementary material/Figure7F-GAPDH-RIG-I.tif]

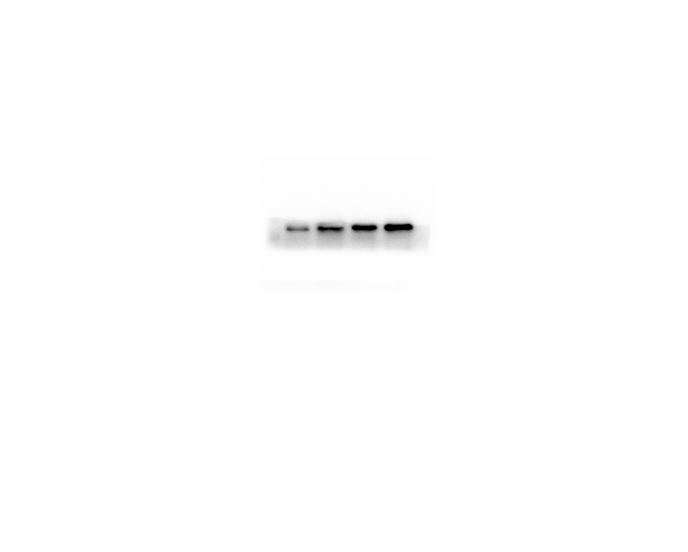

Supplement: Supplementary file 2 [file Data_Sheet_1.zip › supplementary material/Figure7F-SERINC5-CMV.tif]

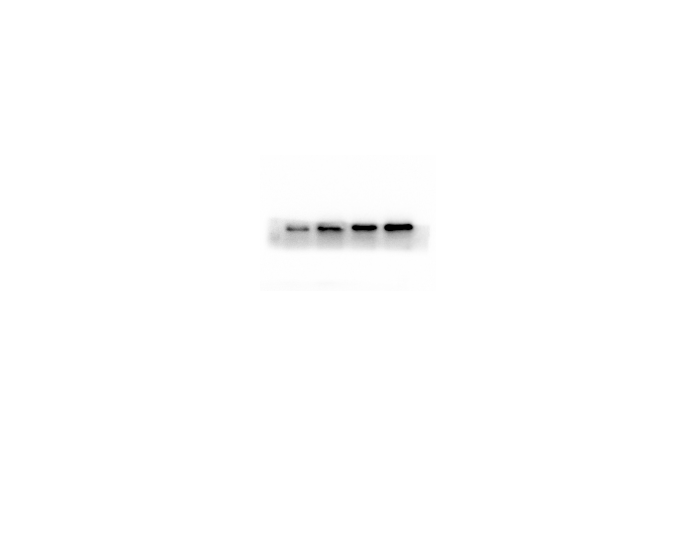

Supplement: Supplementary file 2 [file Data_Sheet_1.zip › supplementary material/Figure7F-SERINC5-HA.tif]

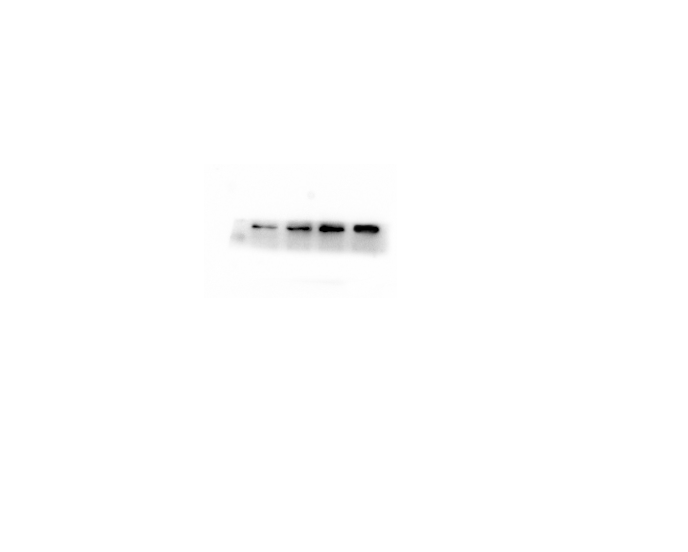

Supplement: Supplementary file 2 [file Data_Sheet_1.zip › supplementary material/Figure7F-SERINC5-MDA5.tif]

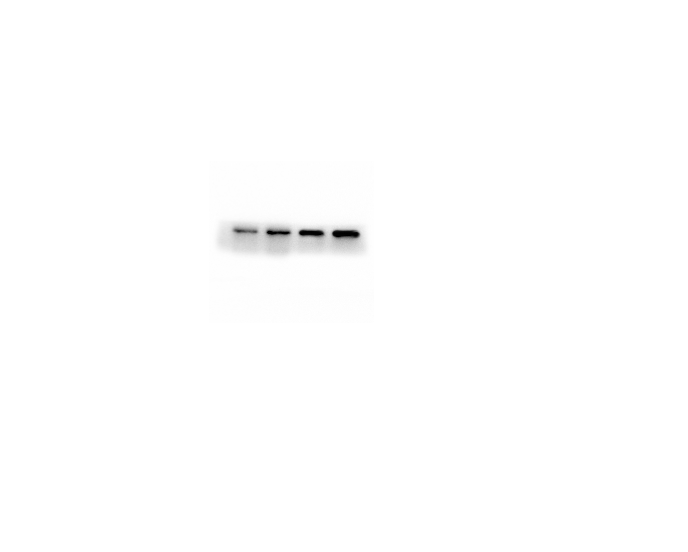

Supplement: Supplementary file 2 [file Data_Sheet_1.zip › supplementary material/Figure7F-SERINC5-RIG-I.tif]

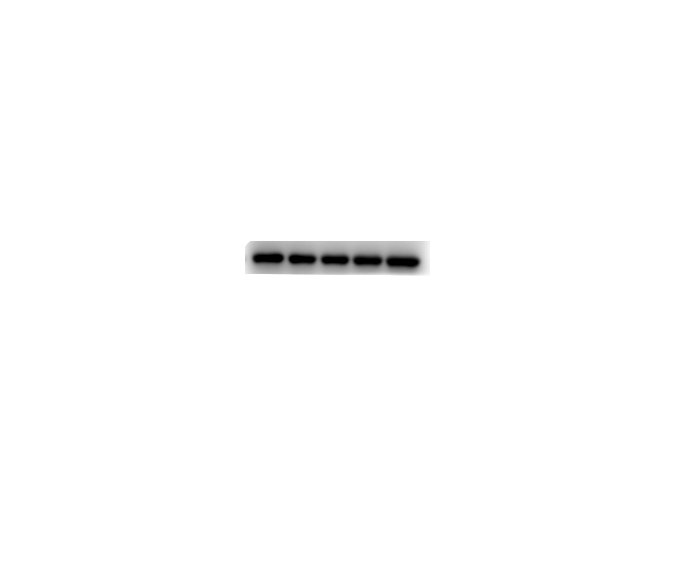

Supplement: Supplementary file 2 [file Data_Sheet_1.zip › supplementary material/Figure8A-GAPDH.tif]

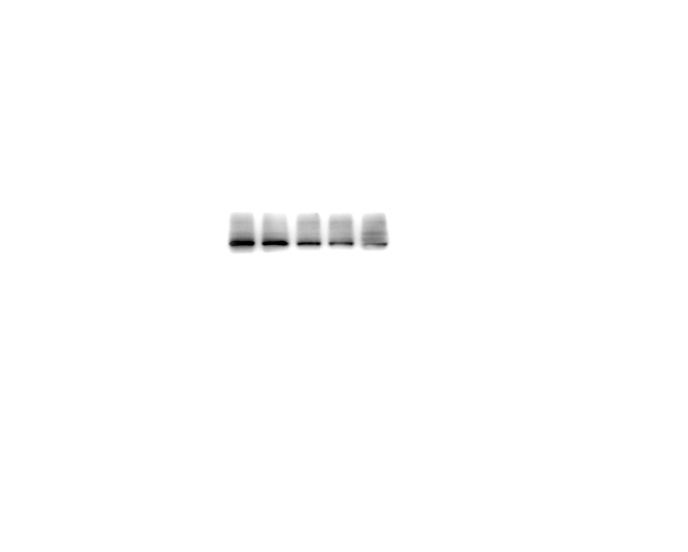

Supplement: Supplementary file 2 [file Data_Sheet_1.zip › supplementary material/Figure8A-siRNA-MDA5.tif]

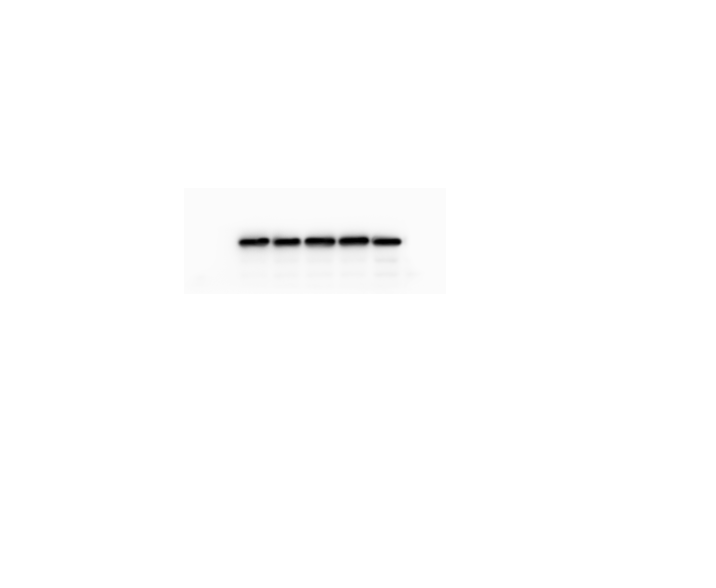

Supplement: Supplementary file 2 [file Data_Sheet_1.zip › supplementary material/Figure8B-GAPDH.tif]

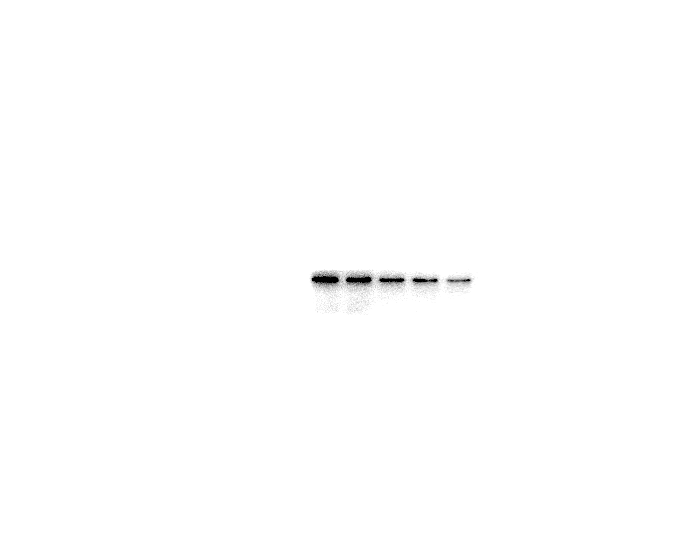

Supplement: Supplementary file 2 [file Data_Sheet_1.zip › supplementary material/Figure8B-siRNA-RIG-I.tif]
