# Supplementary figures and images for: Antiviral Role of Serine Incorporator 5 (SERINC5) Proteins in Classical Swine Fever Virus Infection (part 1 of 2)
Source: Front Microbiol. 2020 Sep 4;11:580233. doi: 10.3389/fmicb.2020.580233 (PMC7498654; doi:10.3389/fmicb.2020.580233)

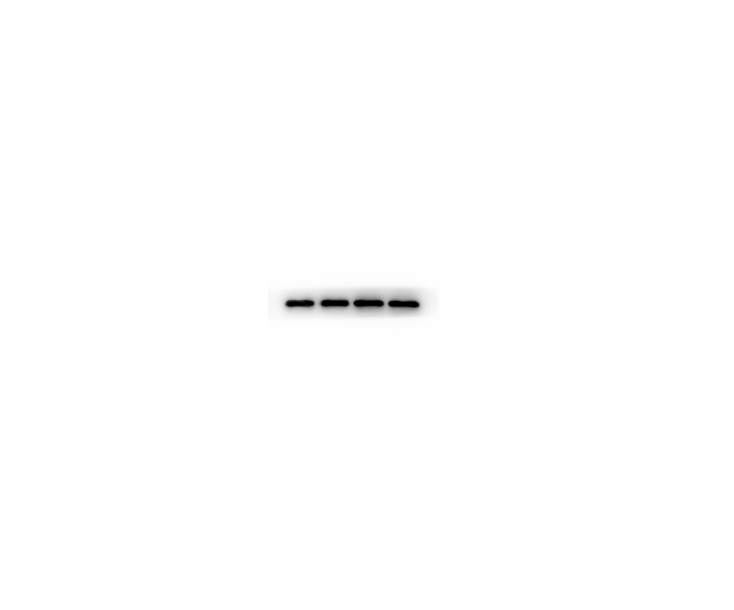

Supplement: Supplementary file 2 [file Data_Sheet_1.zip › supplementary material/Figure1E-GAPDH.tif]

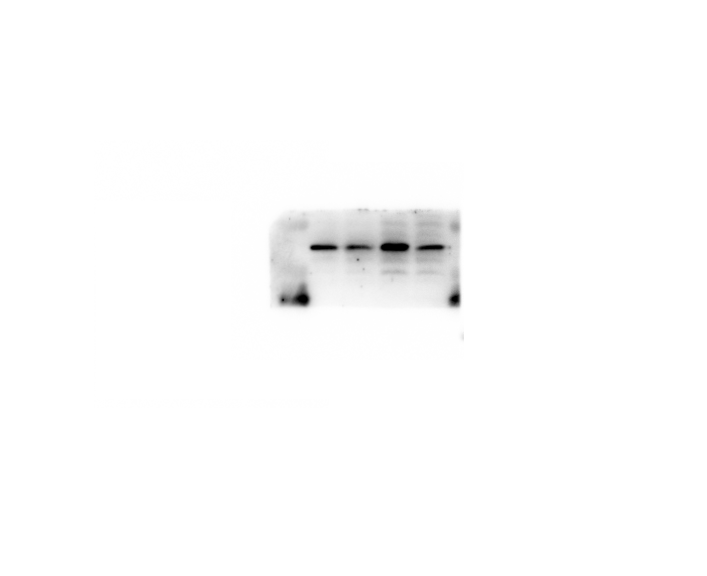

Supplement: Supplementary file 2 [file Data_Sheet_1.zip › supplementary material/Figure1E-Npro.tif]

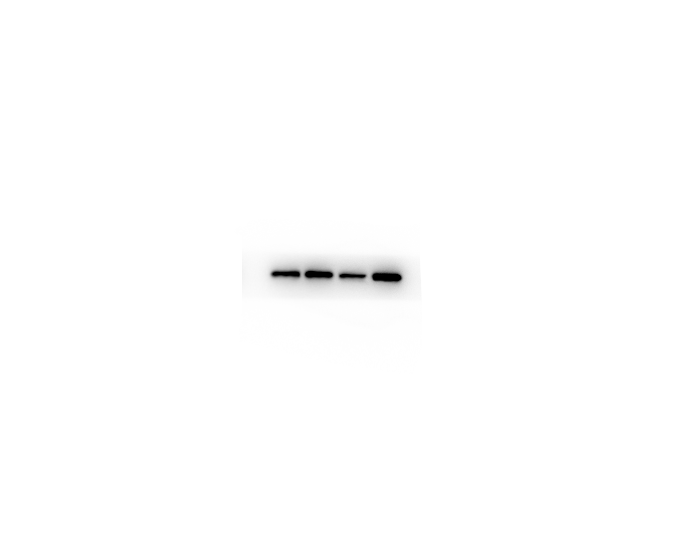

Supplement: Supplementary file 2 [file Data_Sheet_1.zip › supplementary material/Figure1E-SERINC5.tif]

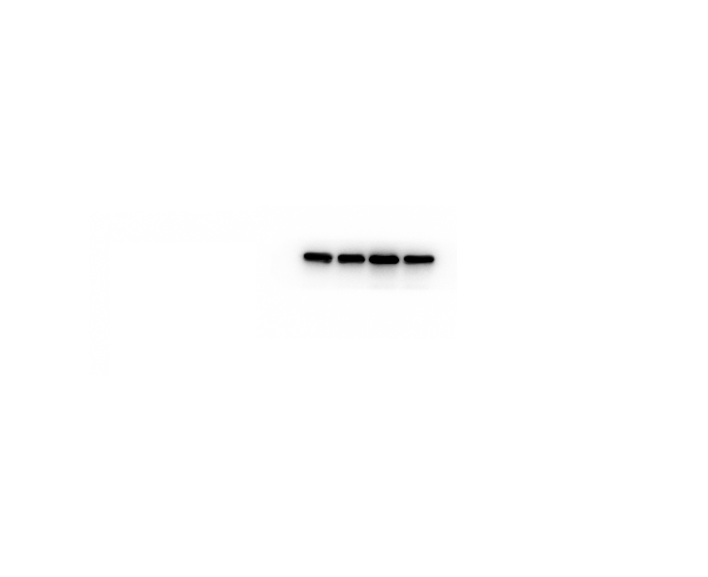

Supplement: Supplementary file 2 [file Data_Sheet_1.zip › supplementary material/Figure1F-GAPDH.tif]

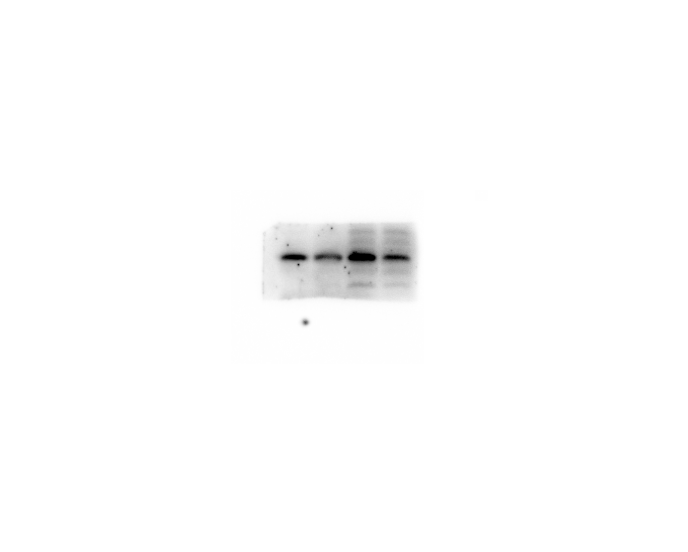

Supplement: Supplementary file 2 [file Data_Sheet_1.zip › supplementary material/Figure1F-Npro.tif]

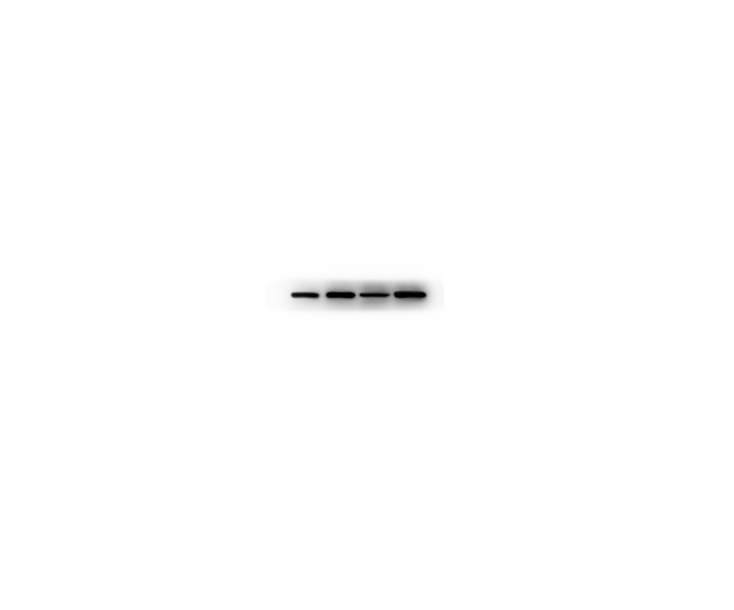

Supplement: Supplementary file 2 [file Data_Sheet_1.zip › supplementary material/Figure1F-SERINC5.tif]

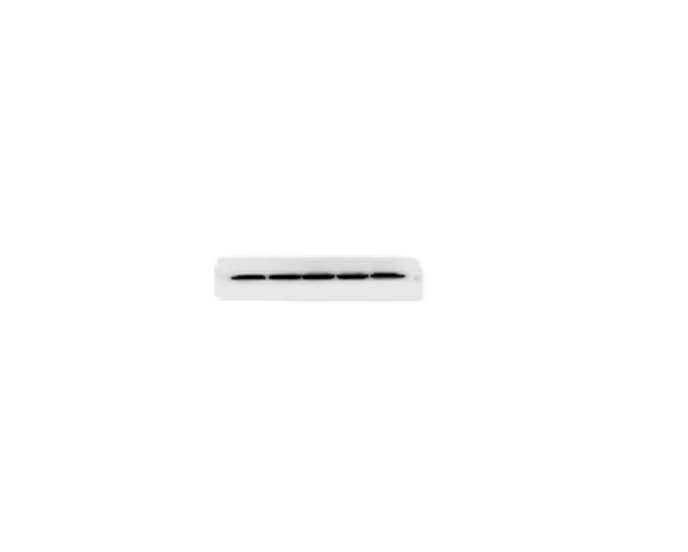

Supplement: Supplementary file 2 [file Data_Sheet_1.zip › supplementary material/Figure2A-GAPDH.tif]

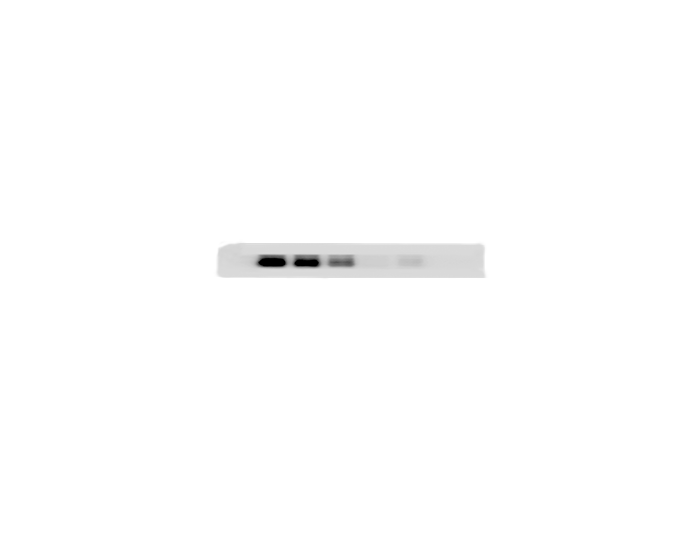

Supplement: Supplementary file 2 [file Data_Sheet_1.zip › supplementary material/Figure2A-SERINC5.tif]

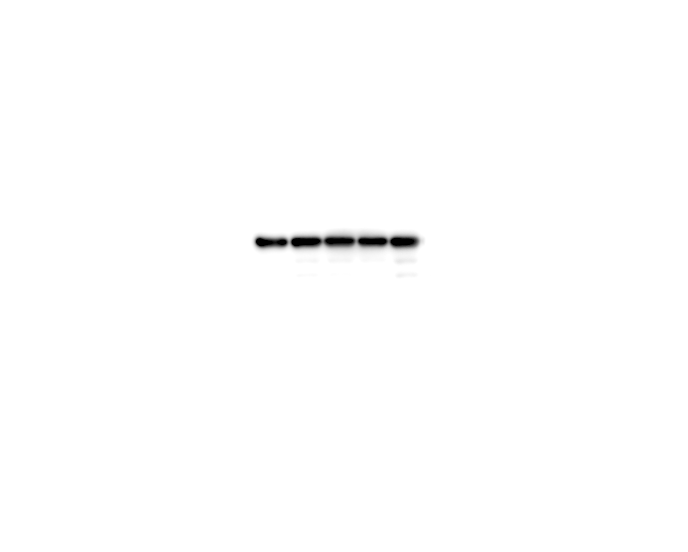

Supplement: Supplementary file 2 [file Data_Sheet_1.zip › supplementary material/Figure2B-GAPDH.tif]

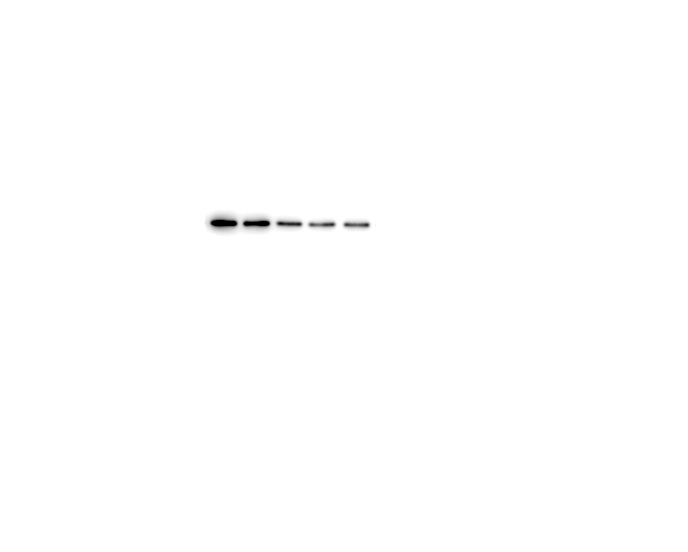

Supplement: Supplementary file 2 [file Data_Sheet_1.zip › supplementary material/Figure2B-SERINC5.tif]

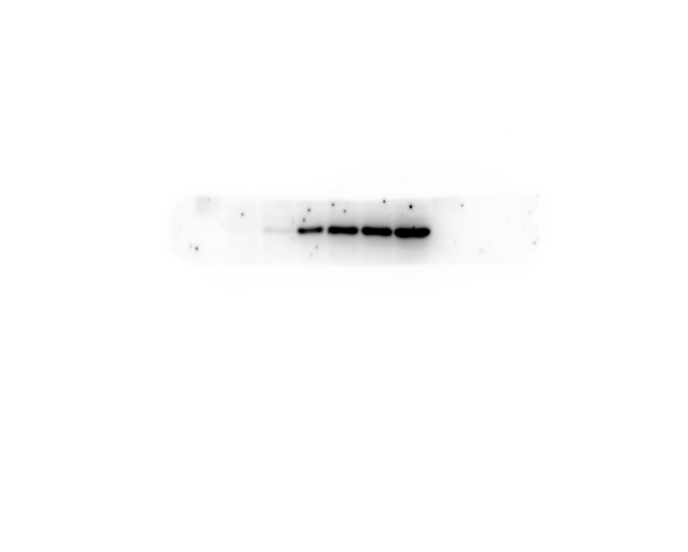

Supplement: Supplementary file 2 [file Data_Sheet_1.zip › supplementary material/Figure3A-CSFV.tif]

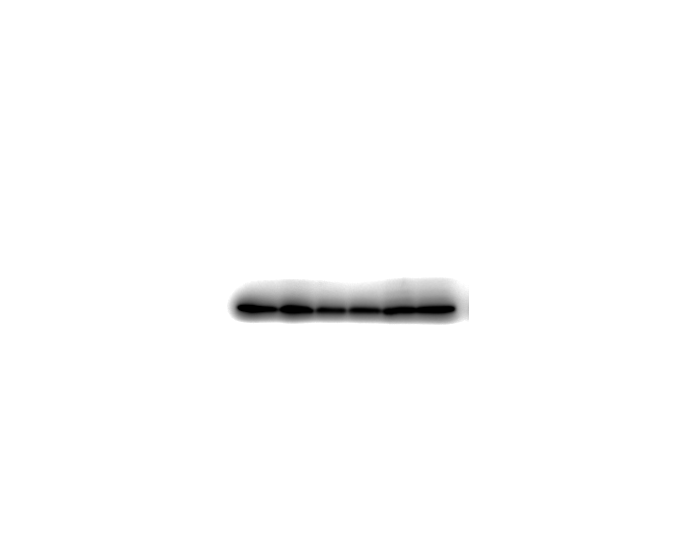

Supplement: Supplementary file 2 [file Data_Sheet_1.zip › supplementary material/Figure3A-GAPDH.tif]

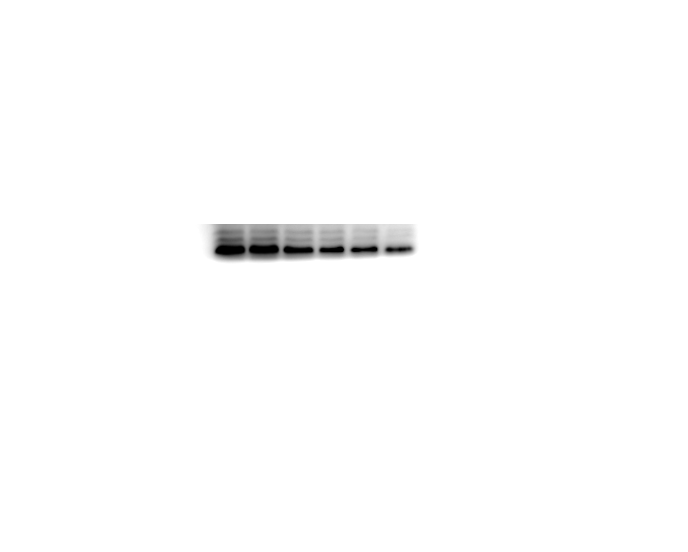

Supplement: Supplementary file 2 [file Data_Sheet_1.zip › supplementary material/Figure3A-SERINC5.tif]

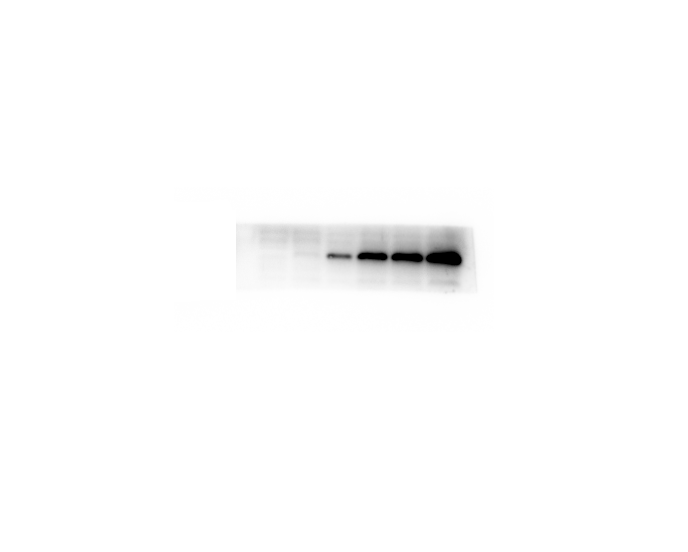

Supplement: Supplementary file 2 [file Data_Sheet_1.zip › supplementary material/Figure3B-CSFV.tif]

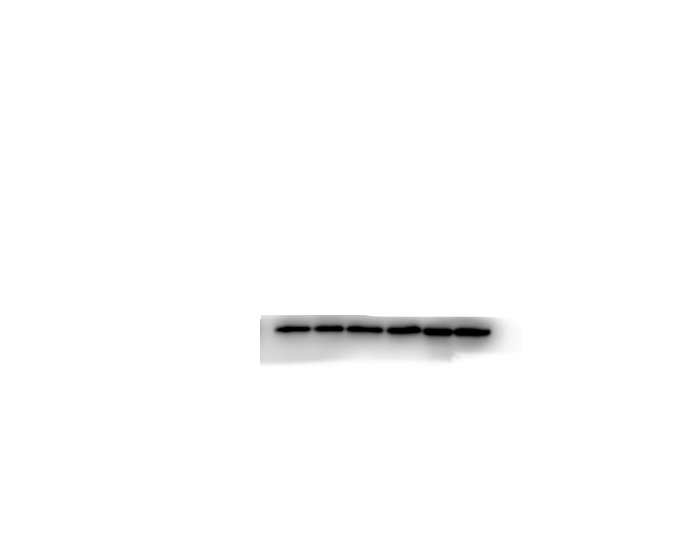

Supplement: Supplementary file 2 [file Data_Sheet_1.zip › supplementary material/Figure3B-GAPDH.tif]

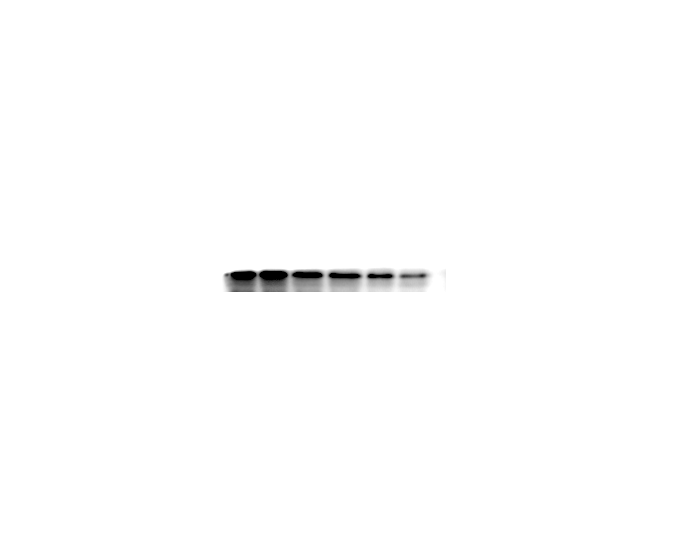

Supplement: Supplementary file 2 [file Data_Sheet_1.zip › supplementary material/Figure3B-SERINC5.tif]

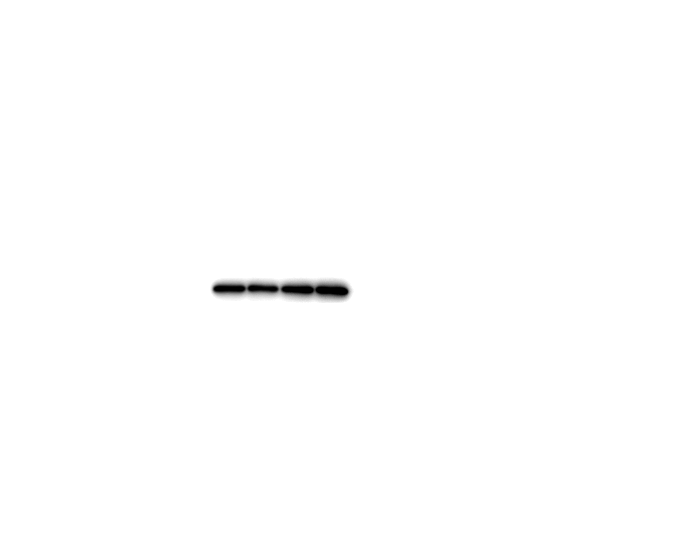

Supplement: Supplementary file 2 [file Data_Sheet_1.zip › supplementary material/Figure3E-GAPDH.tif]

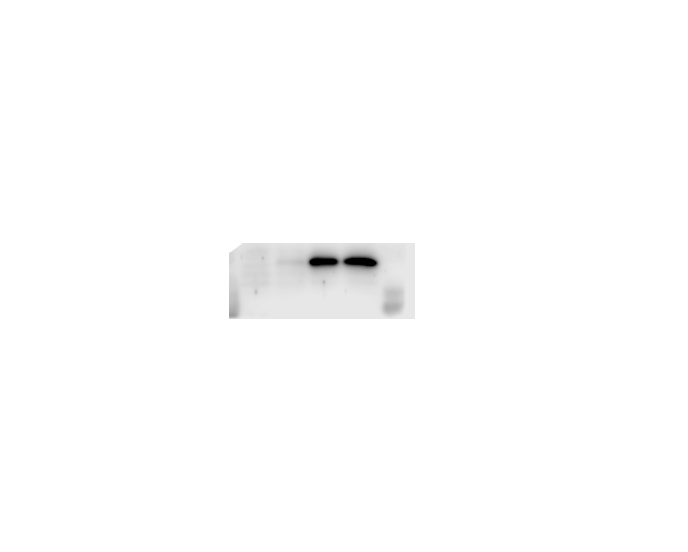

Supplement: Supplementary file 2 [file Data_Sheet_1.zip › supplementary material/Figure3E-Npro.tif]

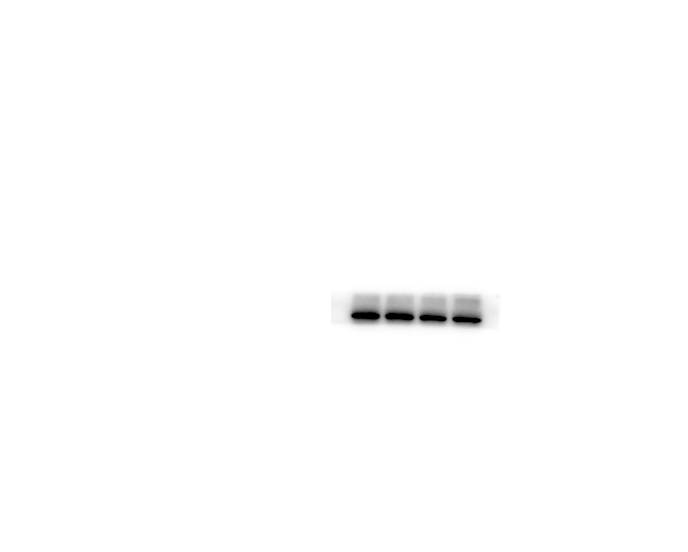

Supplement: Supplementary file 2 [file Data_Sheet_1.zip › supplementary material/Figure3E-SERINC5.tif]

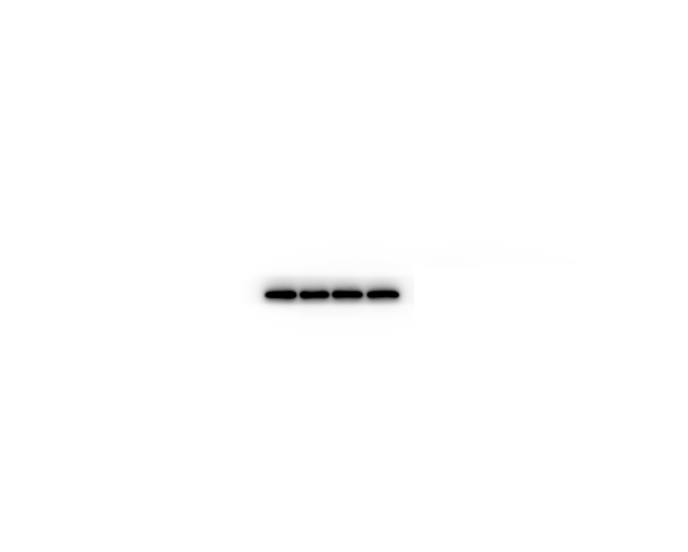

Supplement: Supplementary file 2 [file Data_Sheet_1.zip › supplementary material/Figure3F-GAPDH.tif]

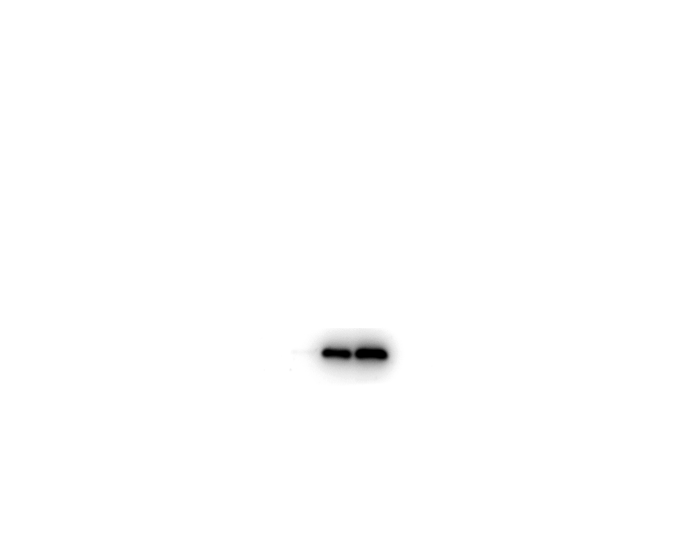

Supplement: Supplementary file 2 [file Data_Sheet_1.zip › supplementary material/Figure3F-Npro.tif]

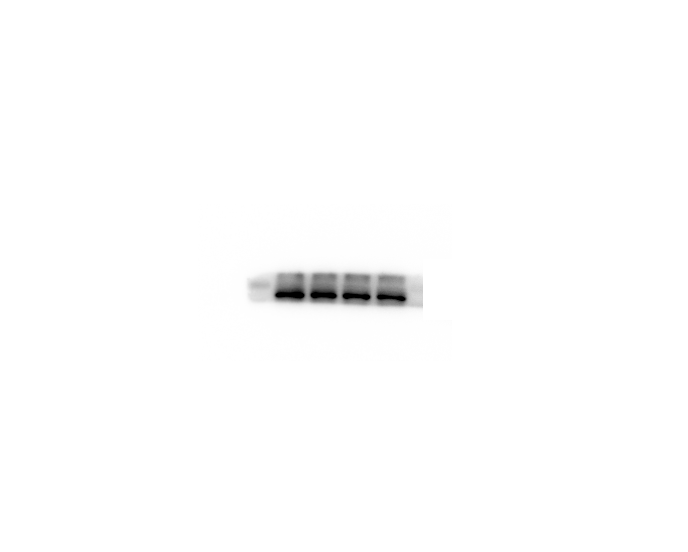

Supplement: Supplementary file 2 [file Data_Sheet_1.zip › supplementary material/Figure3F-SERINC5.tif]

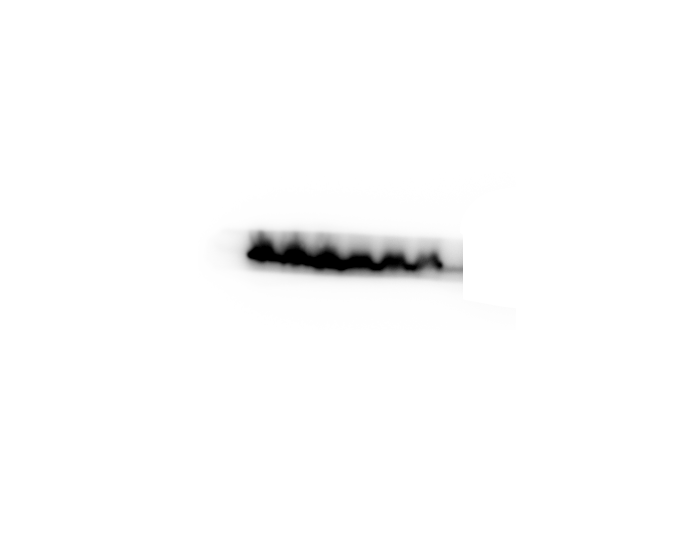

Supplement: Supplementary file 2 [file Data_Sheet_1.zip › supplementary material/Figure4A-Brain-GAPDH.tif]

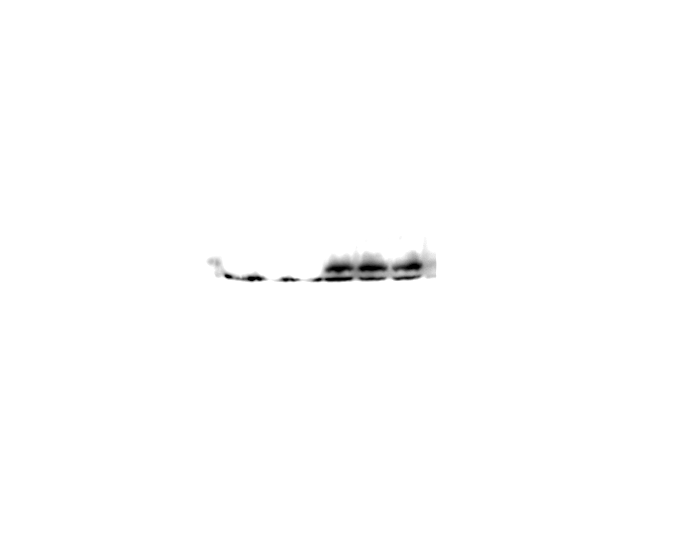

Supplement: Supplementary file 2 [file Data_Sheet_1.zip › supplementary material/Figure4A-Brain-Npro.tif]

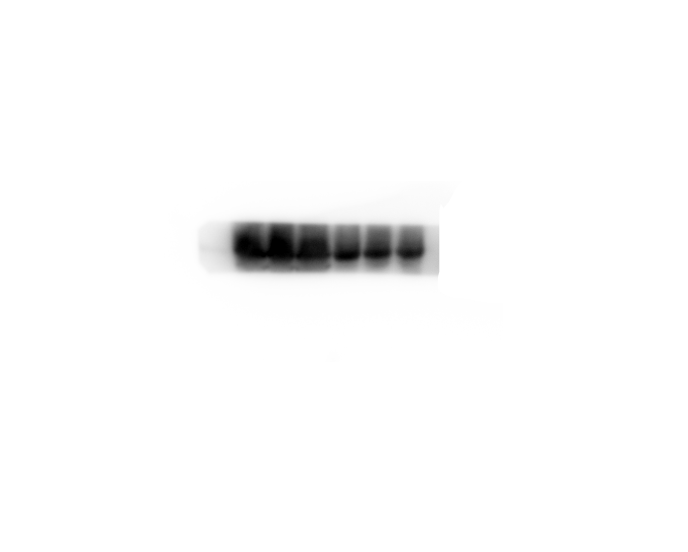

Supplement: Supplementary file 2 [file Data_Sheet_1.zip › supplementary material/Figure4A-Brain-SERINC5.tif]

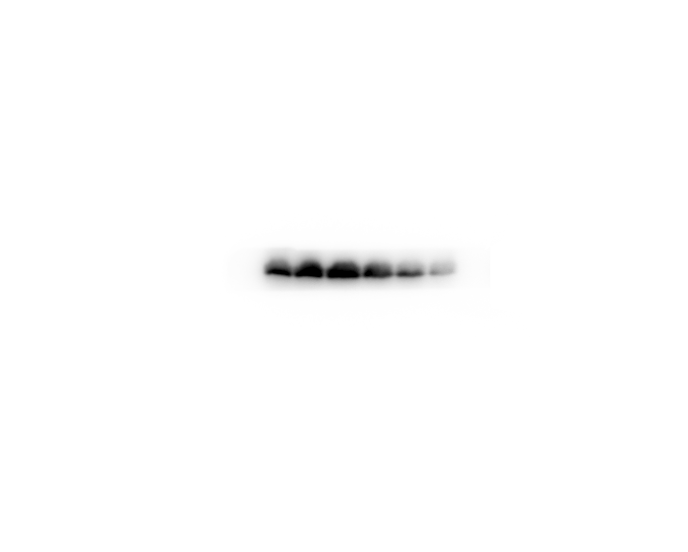

Supplement: Supplementary file 2 [file Data_Sheet_1.zip › supplementary material/Figure4A-Heart-GAPDH.tif]

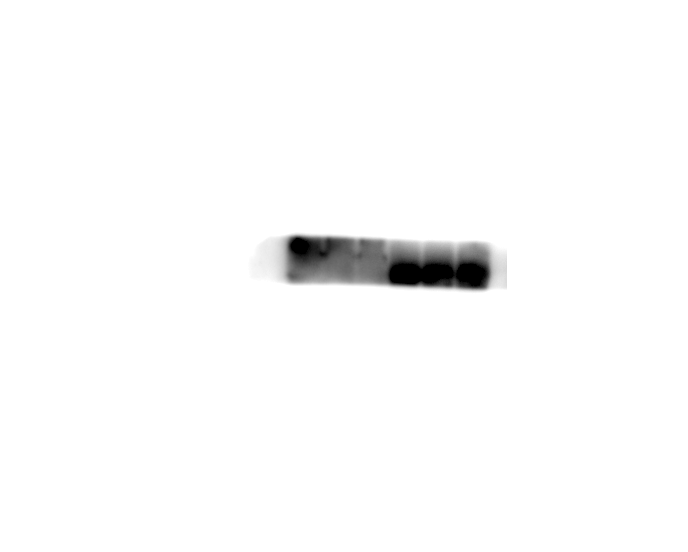

Supplement: Supplementary file 2 [file Data_Sheet_1.zip › supplementary material/Figure4A-Heart-Npro.tif]

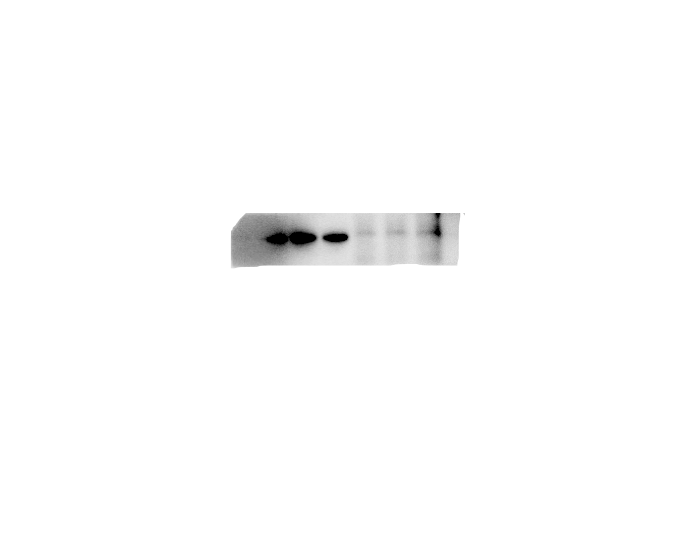

Supplement: Supplementary file 2 [file Data_Sheet_1.zip › supplementary material/Figure4A-Heart-SERINC5.tif]

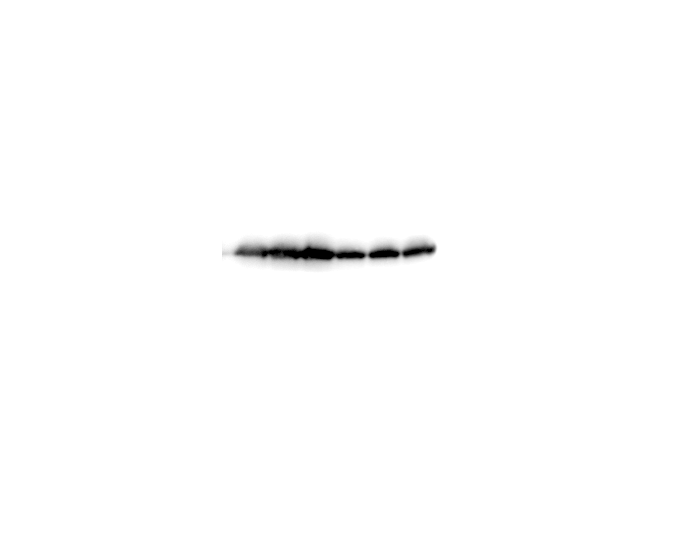

Supplement: Supplementary file 2 [file Data_Sheet_1.zip › supplementary material/Figure4A-Inguinal-lymph-node-GAPDH.tif]

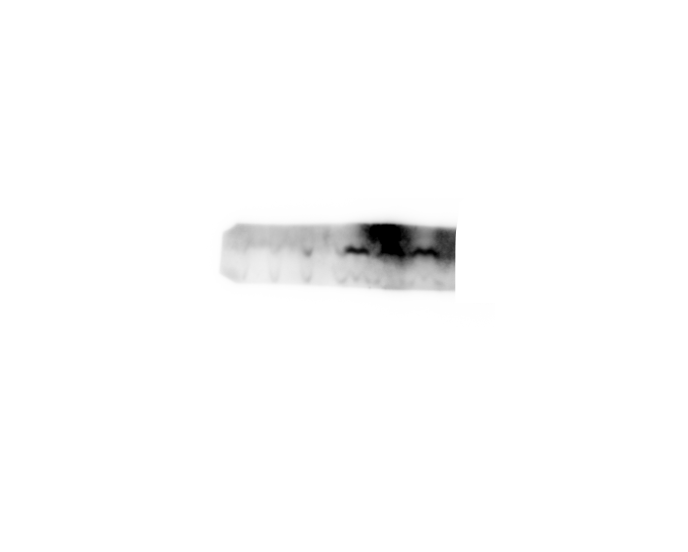

Supplement: Supplementary file 2 [file Data_Sheet_1.zip › supplementary material/Figure4A-Inguinal-lymph-node-Npro.tif]

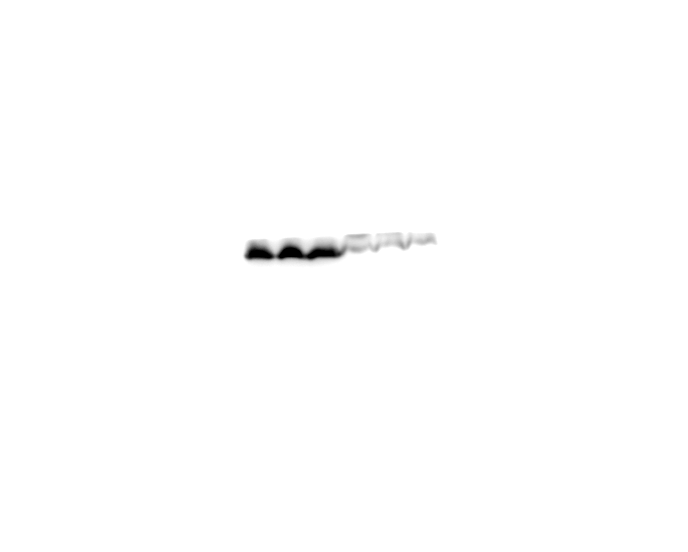

Supplement: Supplementary file 2 [file Data_Sheet_1.zip › supplementary material/Figure4A-Inguinal-lymph-node-SERINC5.tif]

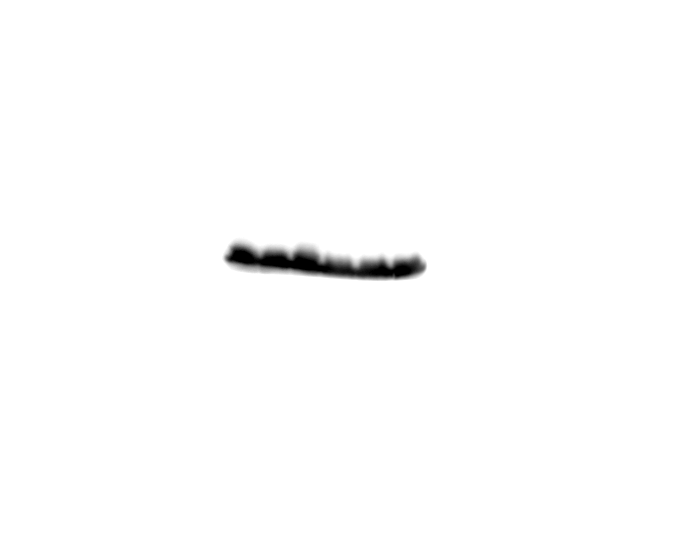

Supplement: Supplementary file 2 [file Data_Sheet_1.zip › supplementary material/Figure4A-Kidney-GAPDH.tif]

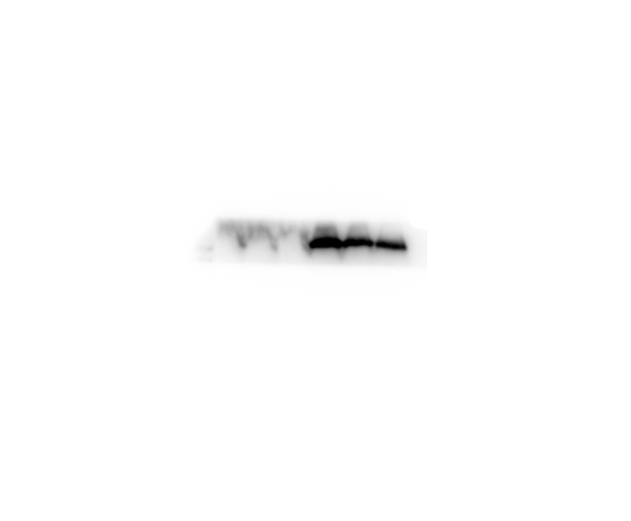

Supplement: Supplementary file 2 [file Data_Sheet_1.zip › supplementary material/Figure4A-Kidney-Npro.tif]

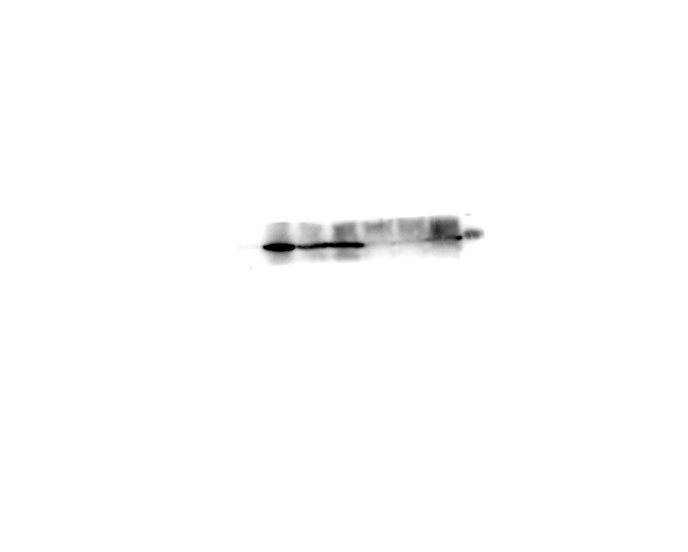

Supplement: Supplementary file 2 [file Data_Sheet_1.zip › supplementary material/Figure4A-Kidney-SERINC5.tif]

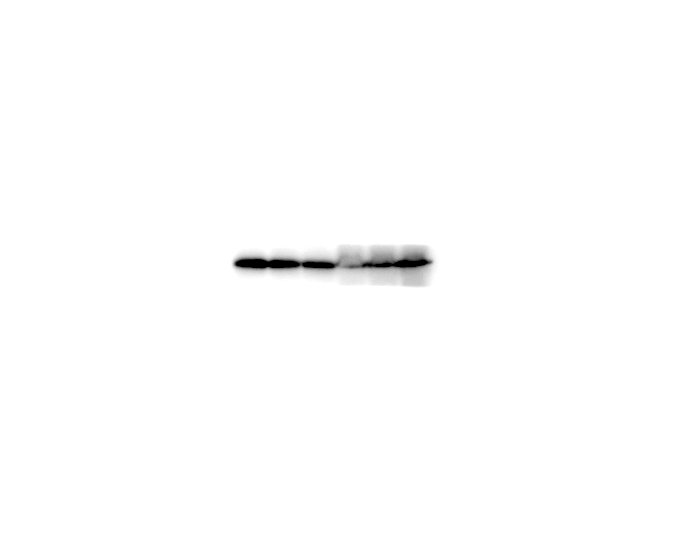

Supplement: Supplementary file 2 [file Data_Sheet_1.zip › supplementary material/Figure4A-Live-SERINC5.tif]

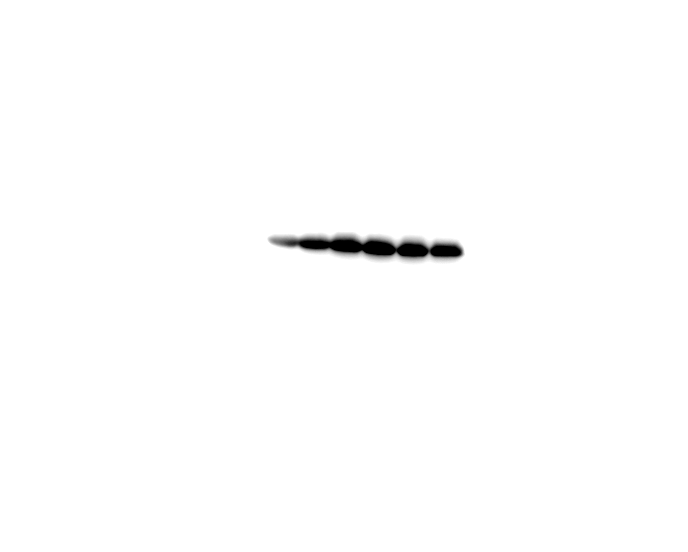

Supplement: Supplementary file 2 [file Data_Sheet_1.zip › supplementary material/Figure4A-Liver-GAPDH.tif]

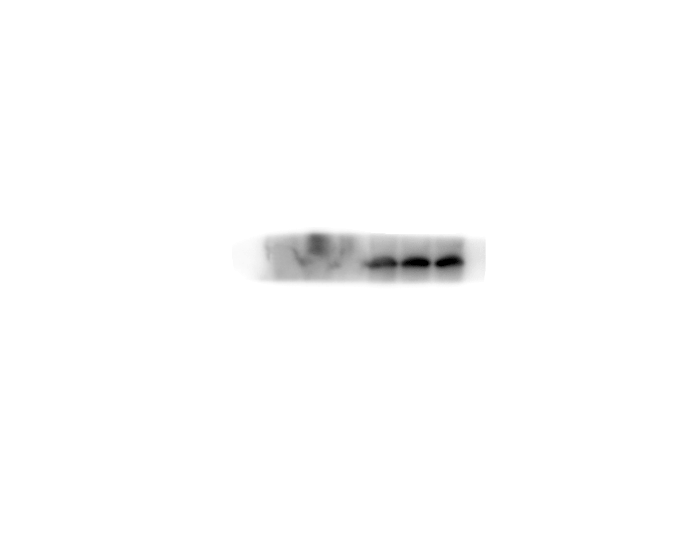

Supplement: Supplementary file 2 [file Data_Sheet_1.zip › supplementary material/Figure4A-Liver-Npro.tif]

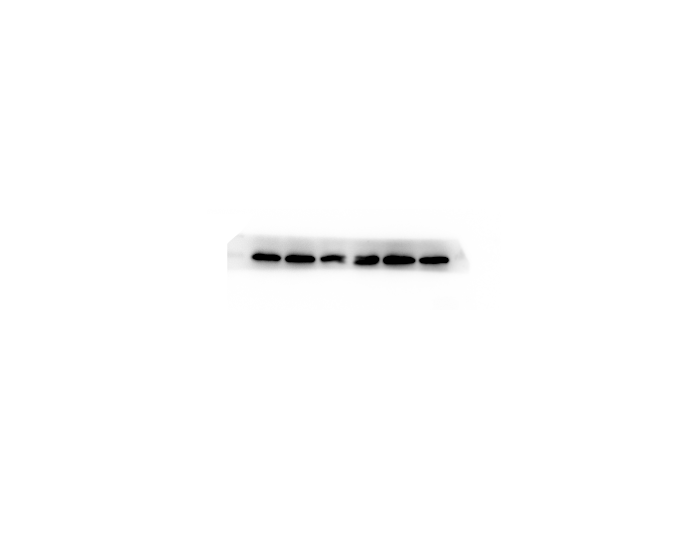

Supplement: Supplementary file 2 [file Data_Sheet_1.zip › supplementary material/Figure4A-Lung-GAPDH.tif]

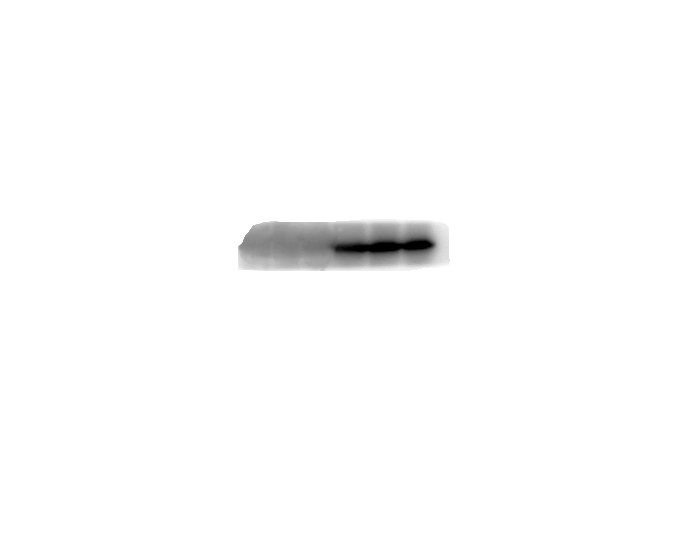

Supplement: Supplementary file 2 [file Data_Sheet_1.zip › supplementary material/Figure4A-Lung-Npro.tif]

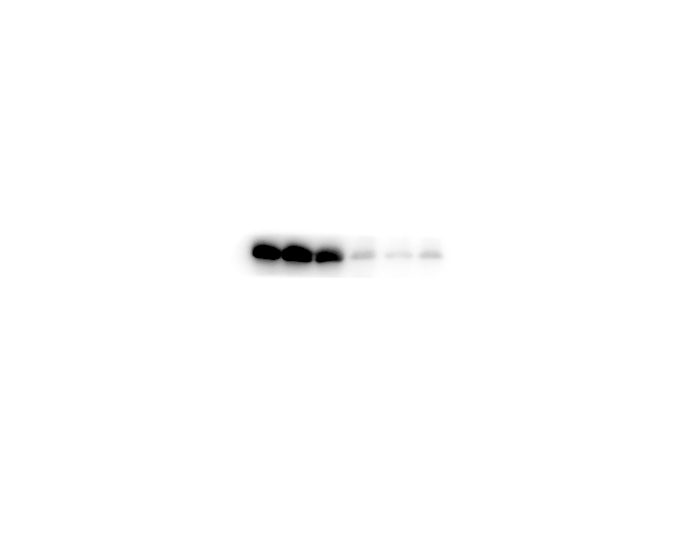

Supplement: Supplementary file 2 [file Data_Sheet_1.zip › supplementary material/Figure4A-Lung-SERINC5.tif]

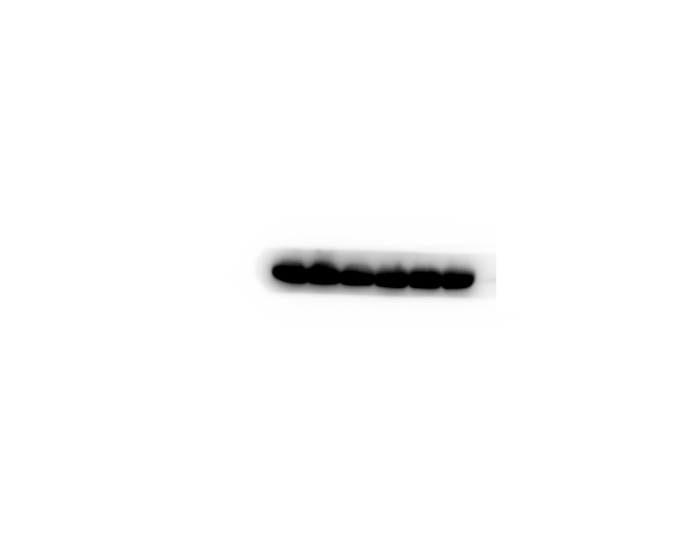

Supplement: Supplementary file 2 [file Data_Sheet_1.zip › supplementary material/Figure4A-Mesenteric-lymph-node-GAPDH.tif]

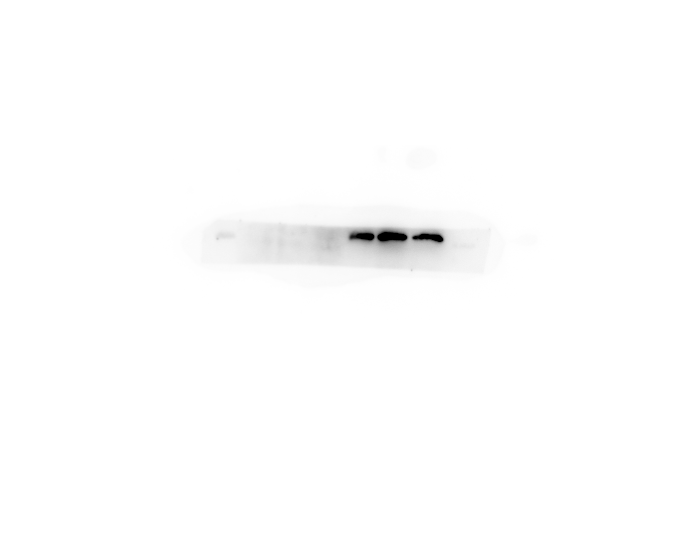

Supplement: Supplementary file 2 [file Data_Sheet_1.zip › supplementary material/Figure4A-Mesenteric-lymph-node-Npro.tif]

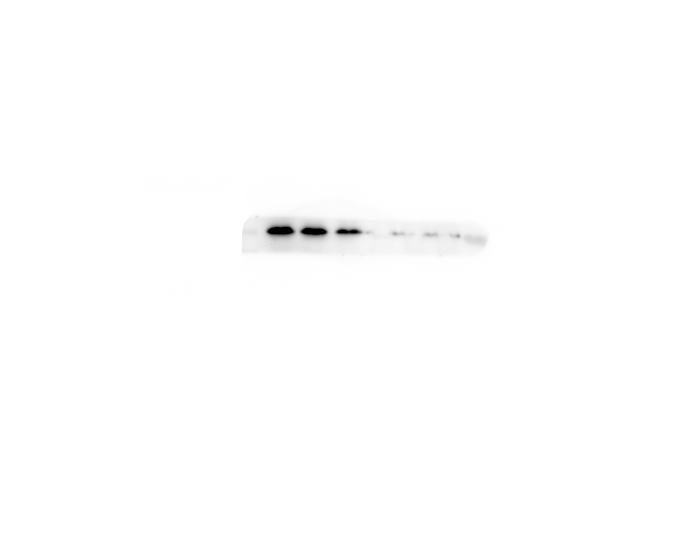

Supplement: Supplementary file 2 [file Data_Sheet_1.zip › supplementary material/Figure4A-Mesenteric-lymph-node-SERINC5.tif]

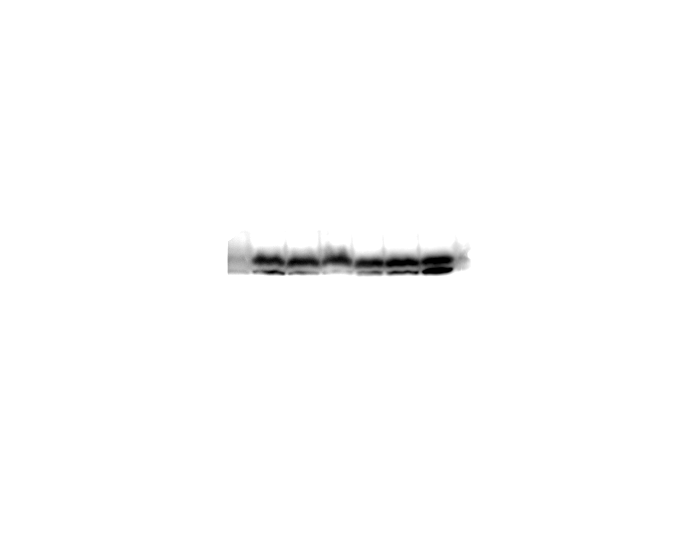

Supplement: Supplementary file 2 [file Data_Sheet_1.zip › supplementary material/Figure4A-Spleen-GAPDH.tif]

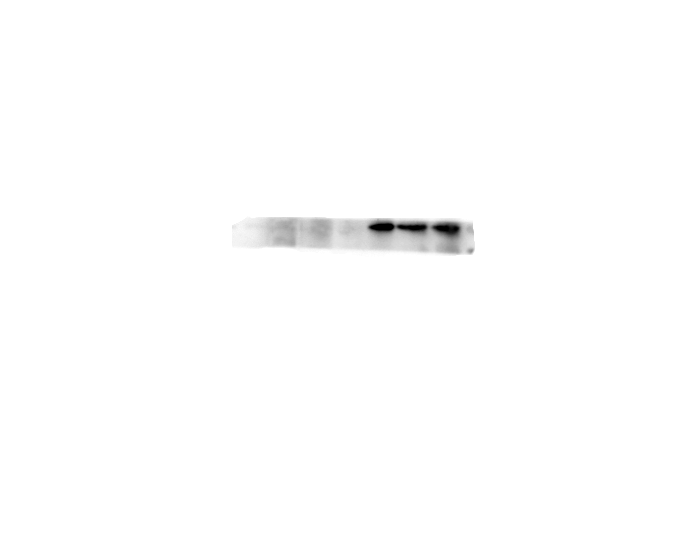

Supplement: Supplementary file 2 [file Data_Sheet_1.zip › supplementary material/Figure4A-Spleen-Npro.tif]

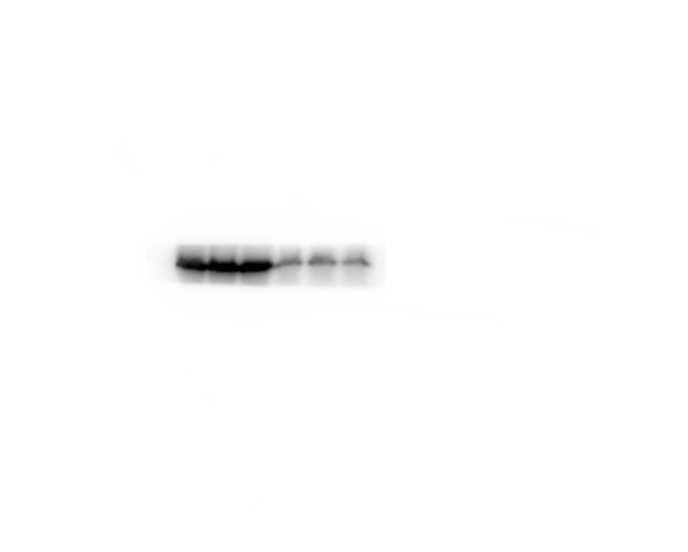

Supplement: Supplementary file 2 [file Data_Sheet_1.zip › supplementary material/Figure4A-Spleen-SERINC5.tif]

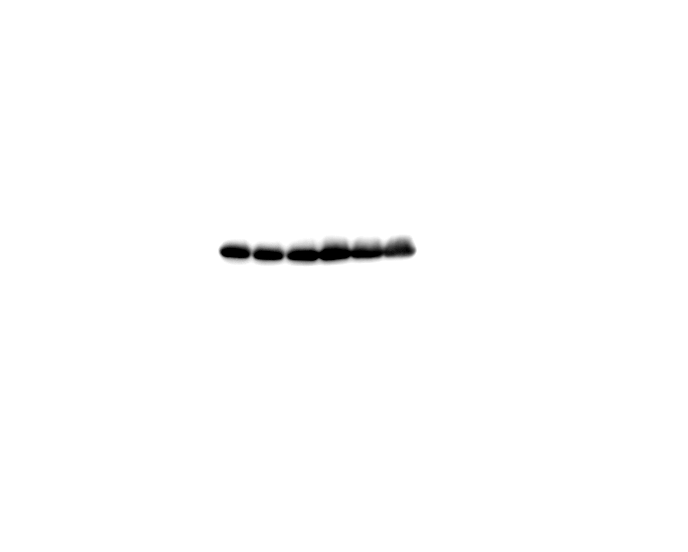

Supplement: Supplementary file 2 [file Data_Sheet_1.zip › supplementary material/Figure4A-Thymus-GAPDH.tif]

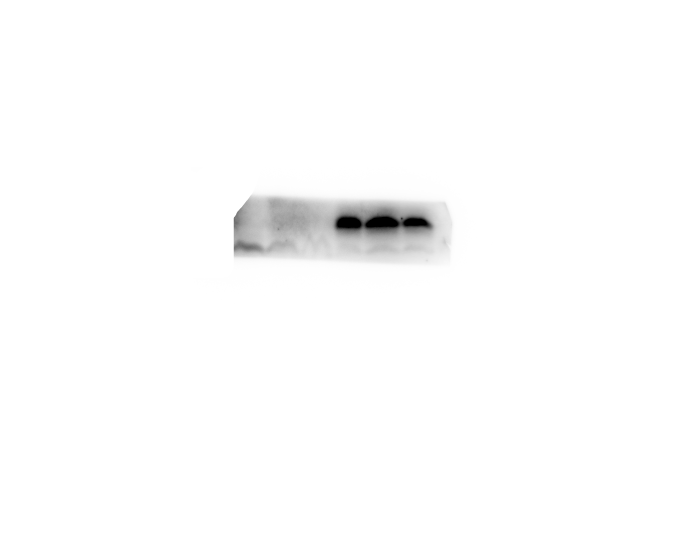

Supplement: Supplementary file 2 [file Data_Sheet_1.zip › supplementary material/Figure4A-Thymus-Npro.tif]

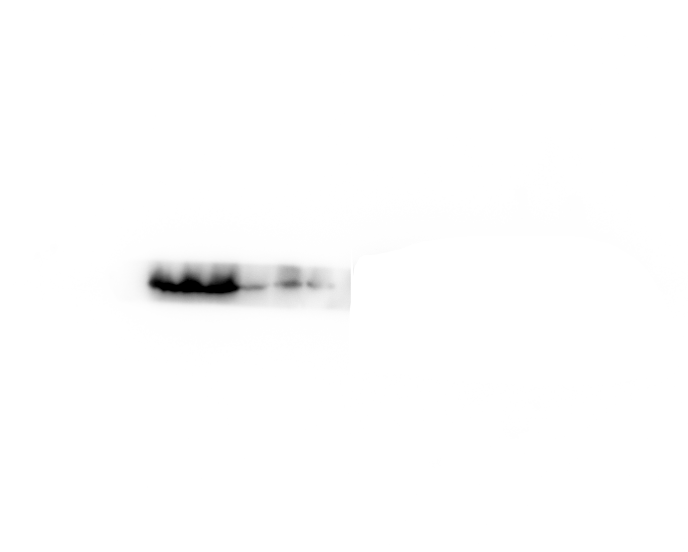

Supplement: Supplementary file 2 [file Data_Sheet_1.zip › supplementary material/Figure4A-Thymus-SERINC5.tif]

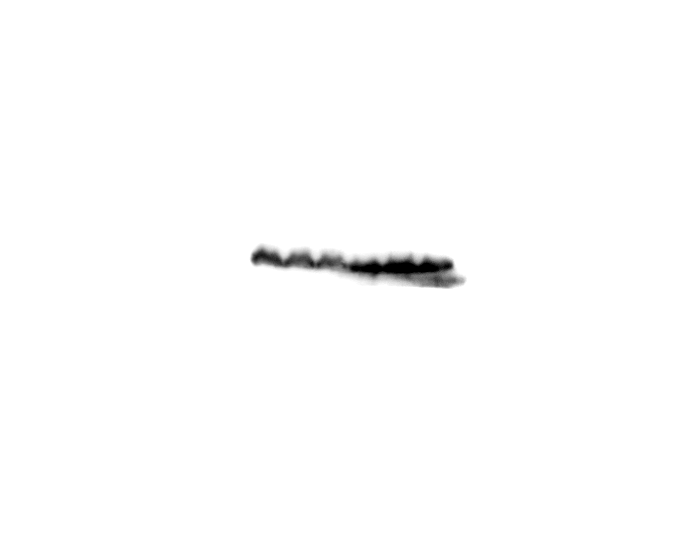

Supplement: Supplementary file 2 [file Data_Sheet_1.zip › supplementary material/Figure4A-Tonsil-GAPDH.tif]

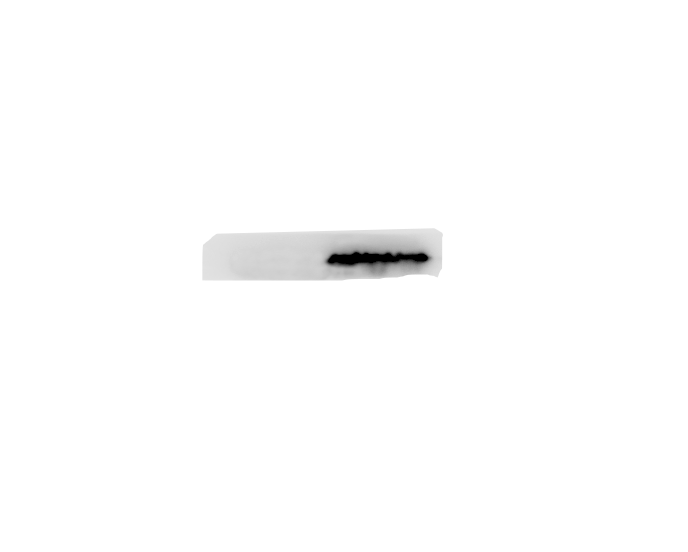

Supplement: Supplementary file 2 [file Data_Sheet_1.zip › supplementary material/Figure4A-Tonsil-Npro.tif]

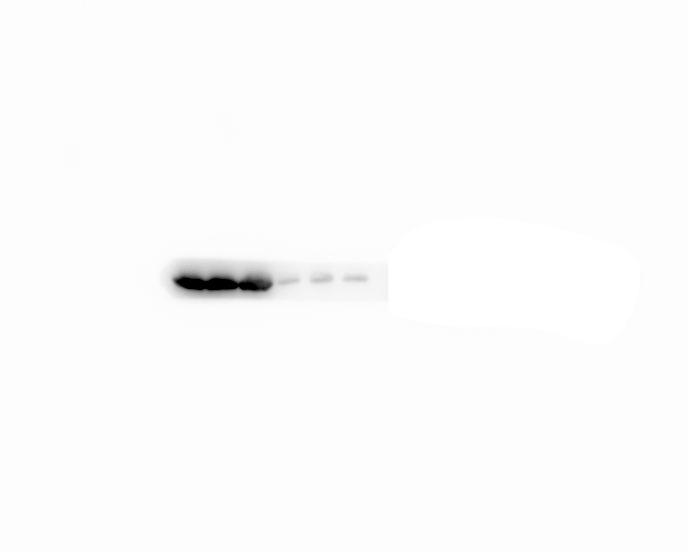

Supplement: Supplementary file 2 [file Data_Sheet_1.zip › supplementary material/Figure4A-Tonsil-SERINC5.tif]

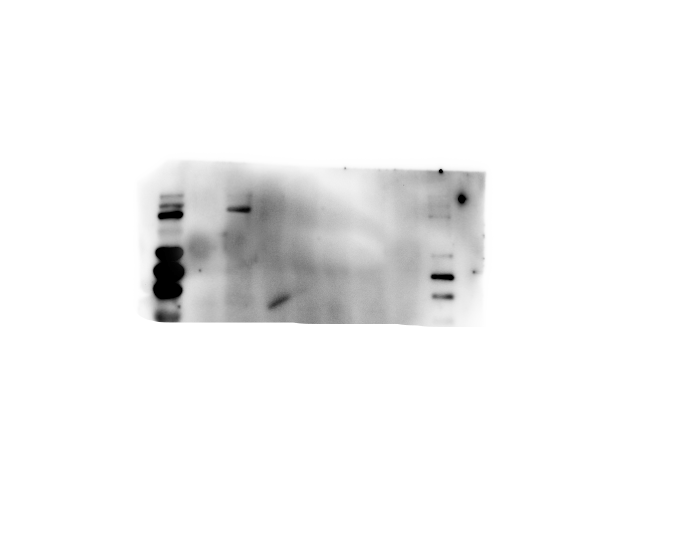

Supplement: Supplementary file 2 [file Data_Sheet_1.zip › supplementary material/Figure5A-IB-HA.tif]

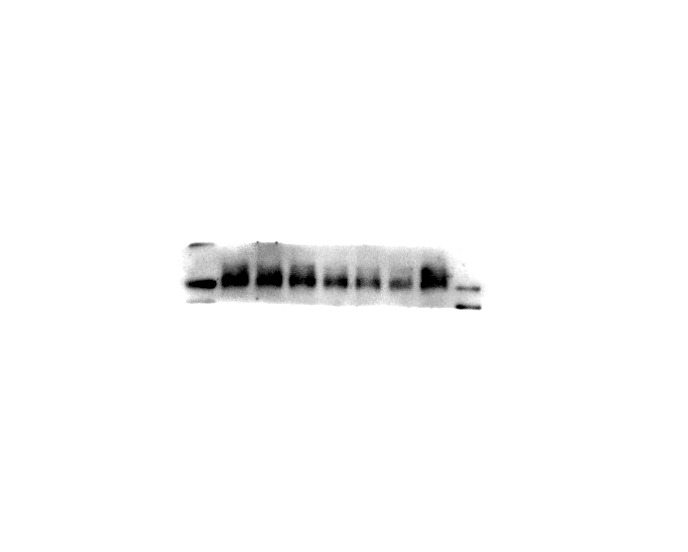

Supplement: Supplementary file 2 [file Data_Sheet_1.zip › supplementary material/Figure5A-IB-SERINC5.tif]

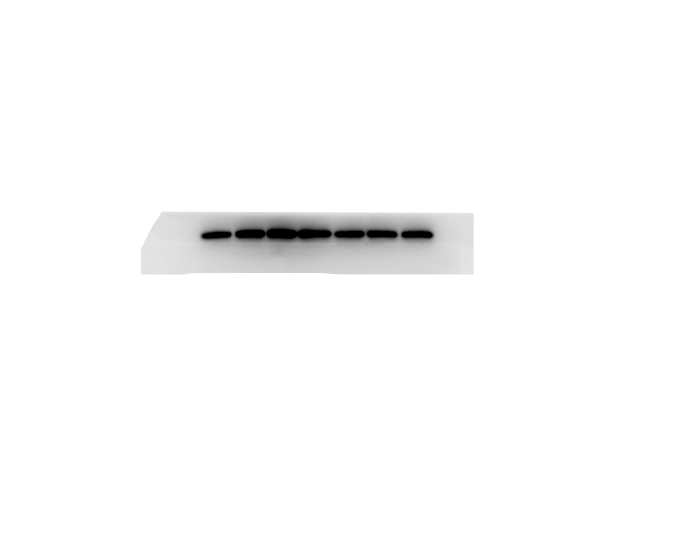

Supplement: Supplementary file 2 [file Data_Sheet_1.zip › supplementary material/Figure5A-WCL-GAPDH.tif]

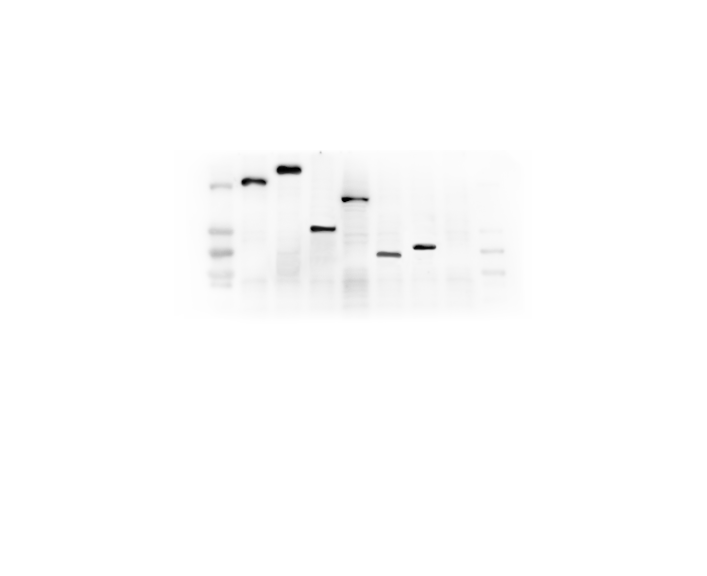

Supplement: Supplementary file 2 [file Data_Sheet_1.zip › supplementary material/Figure5A-WCL-HA.tif]

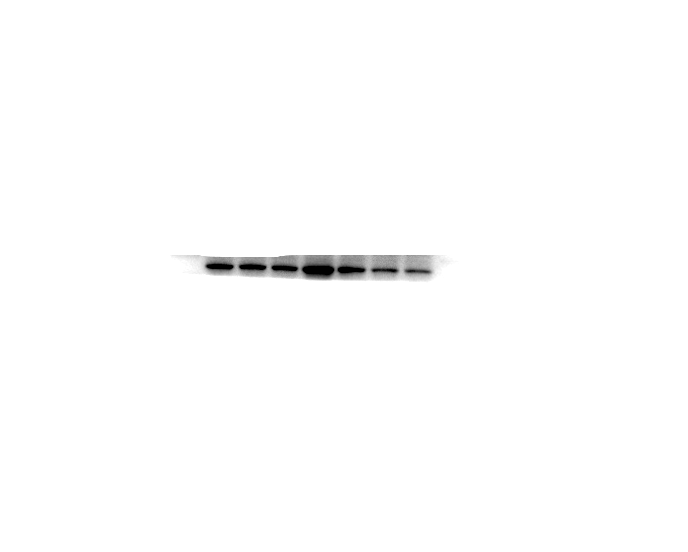

Supplement: Supplementary file 2 [file Data_Sheet_1.zip › supplementary material/Figure5A-WCL-SERINC5.tif]

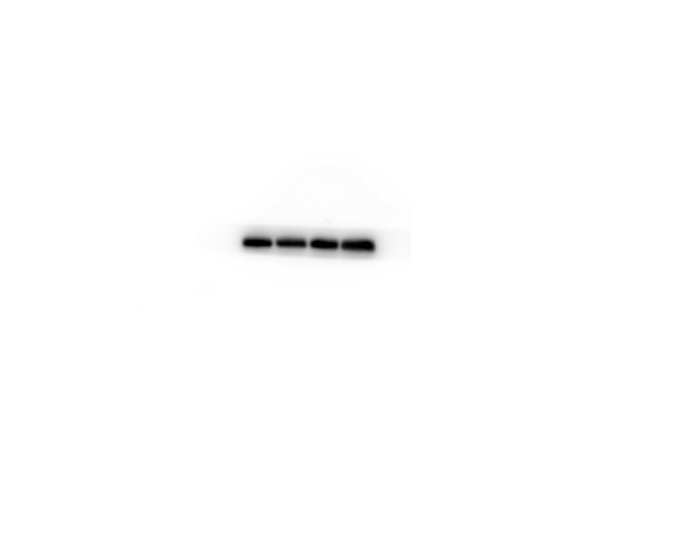

Supplement: Supplementary file 2 [file Data_Sheet_1.zip › supplementary material/Figure6B(1)-GAPDH.tif]

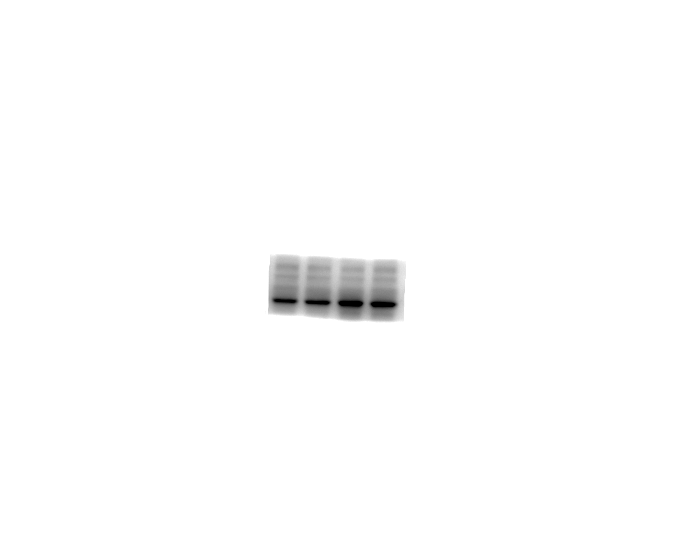

Supplement: Supplementary file 2 [file Data_Sheet_1.zip › supplementary material/Figure6B(1)-SERINC5.tif]

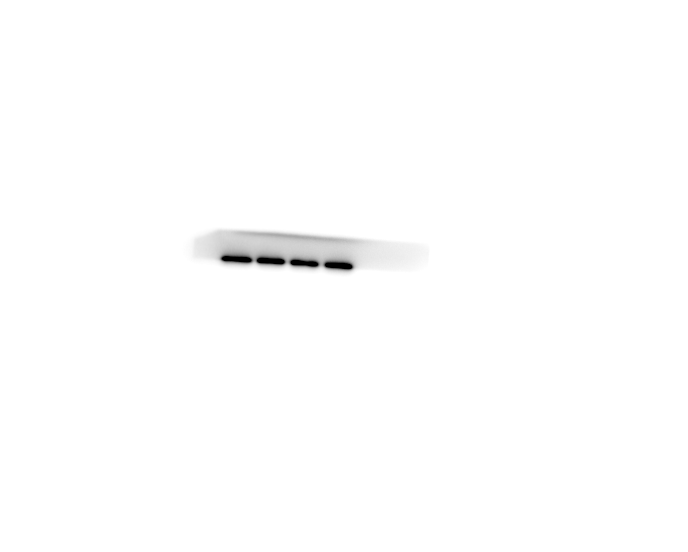

Supplement: Supplementary file 2 [file Data_Sheet_1.zip › supplementary material/Figure6B(2)-GAPDH.tif]

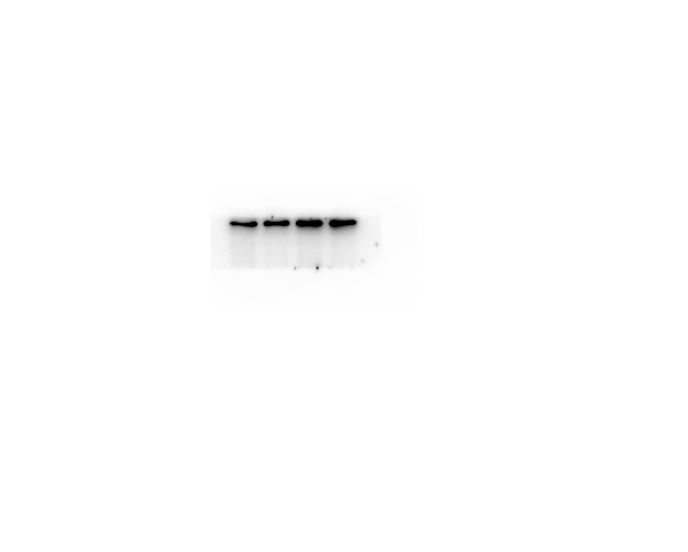

Supplement: Supplementary file 2 [file Data_Sheet_1.zip › supplementary material/Figure6B(2)-SERINC5.tif]

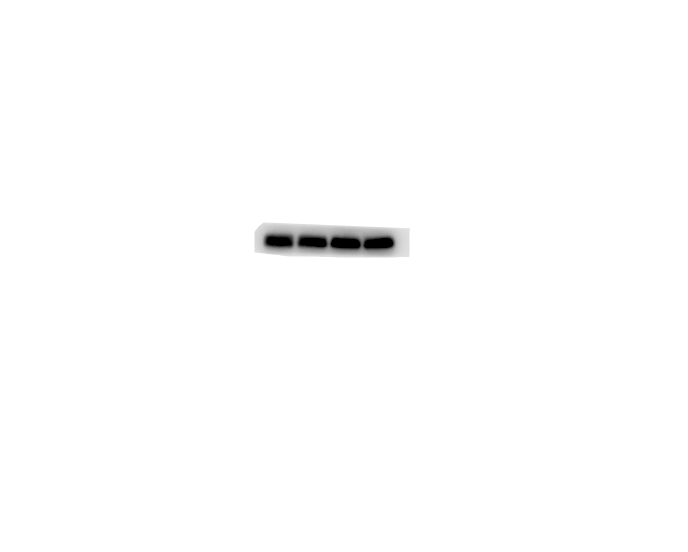

Supplement: Supplementary file 2 [file Data_Sheet_1.zip › supplementary material/Figure6B(3)-GAPDH.tif]

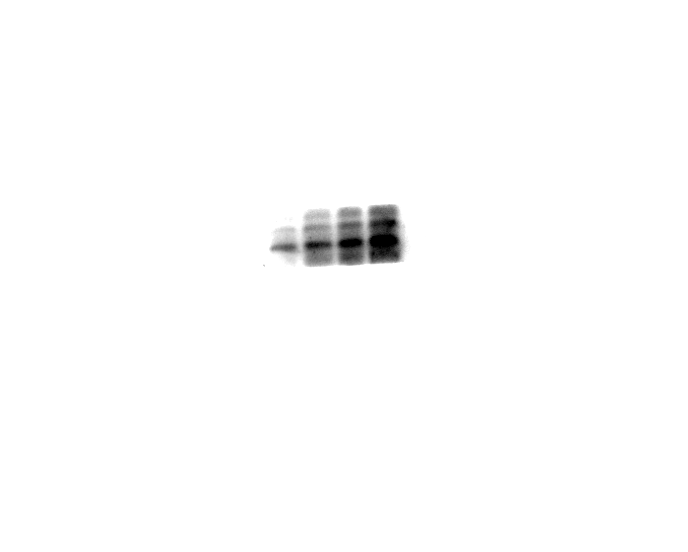

Supplement: Supplementary file 2 [file Data_Sheet_1.zip › supplementary material/Figure6B(3)-SERINC5.tif]

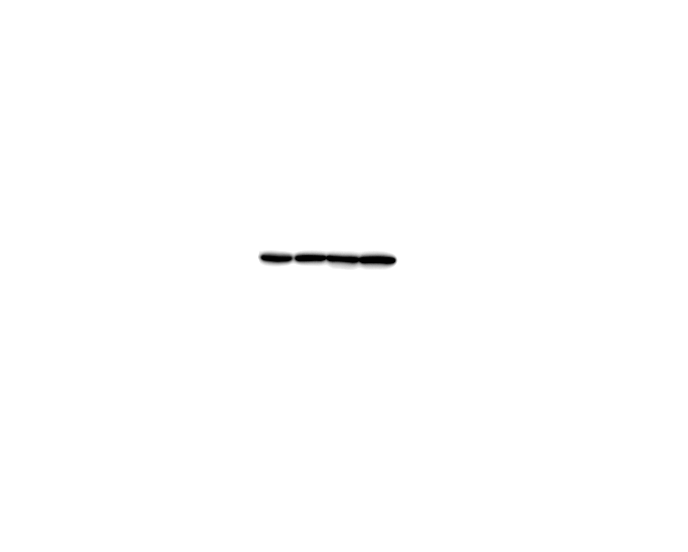

Supplement: Supplementary file 2 [file Data_Sheet_1.zip › supplementary material/Figure6C(1)-GAPDH.tif]

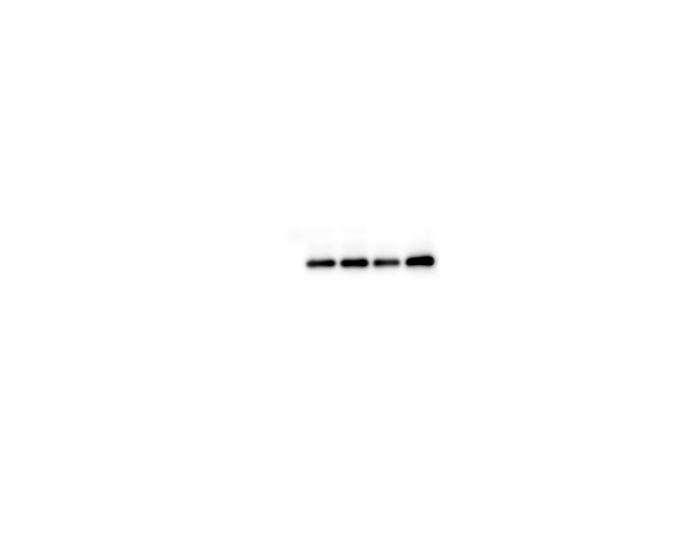

Supplement: Supplementary file 2 [file Data_Sheet_1.zip › supplementary material/Figure6C(1)-SERINC5.tif]

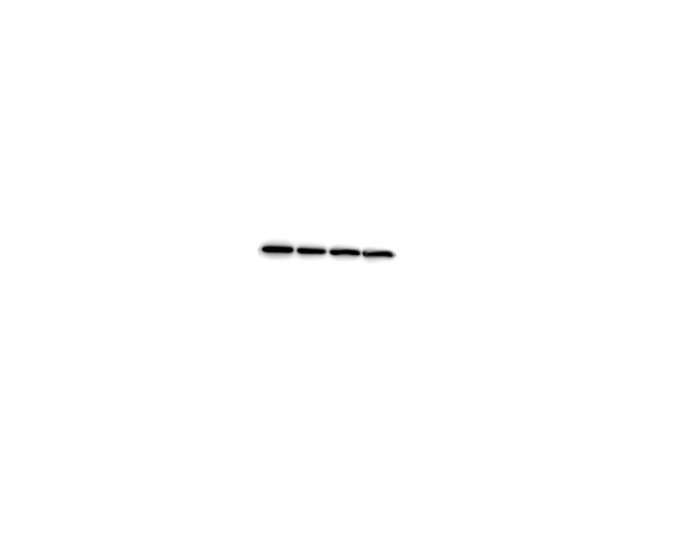

Supplement: Supplementary file 2 [file Data_Sheet_1.zip › supplementary material/Figure6C(2)-GAPDH.tif]

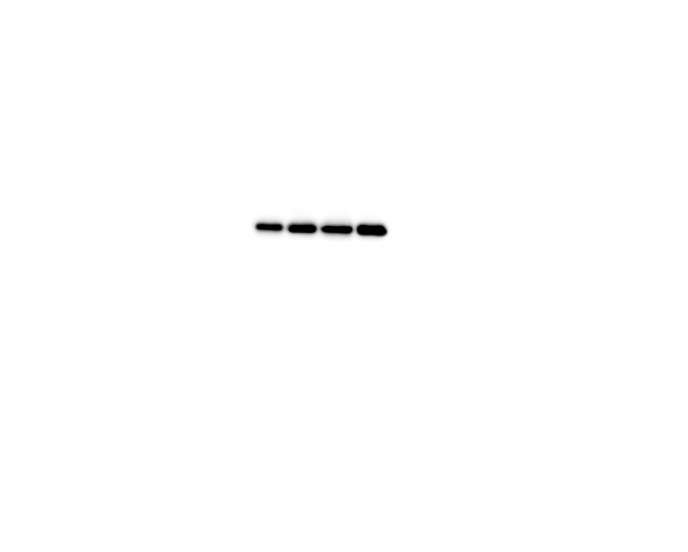

Supplement: Supplementary file 2 [file Data_Sheet_1.zip › supplementary material/Figure6C(2)-SERINC5.tif]

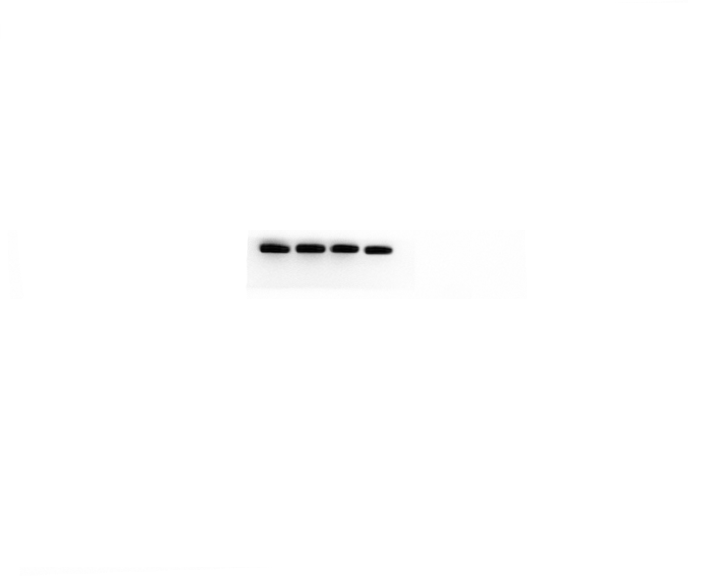

Supplement: Supplementary file 2 [file Data_Sheet_1.zip › supplementary material/Figure7A-GAPDH-IFR3.tif]

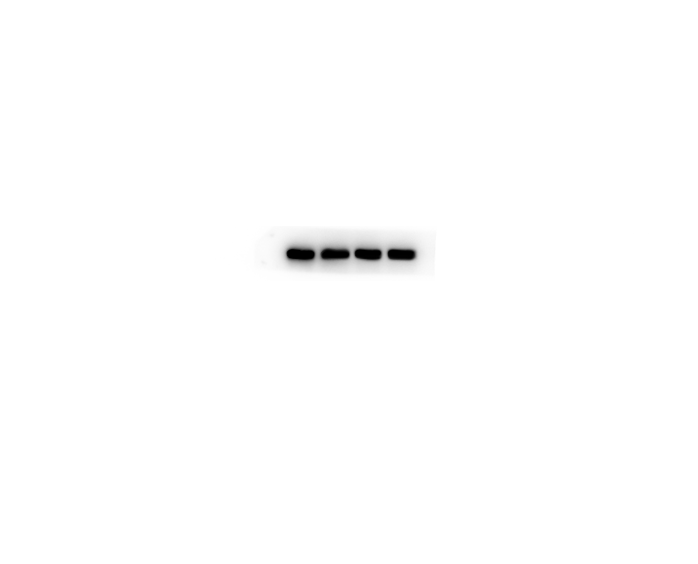

Supplement: Supplementary file 2 [file Data_Sheet_1.zip › supplementary material/Figure7A-GAPDH-IRF7.tif]

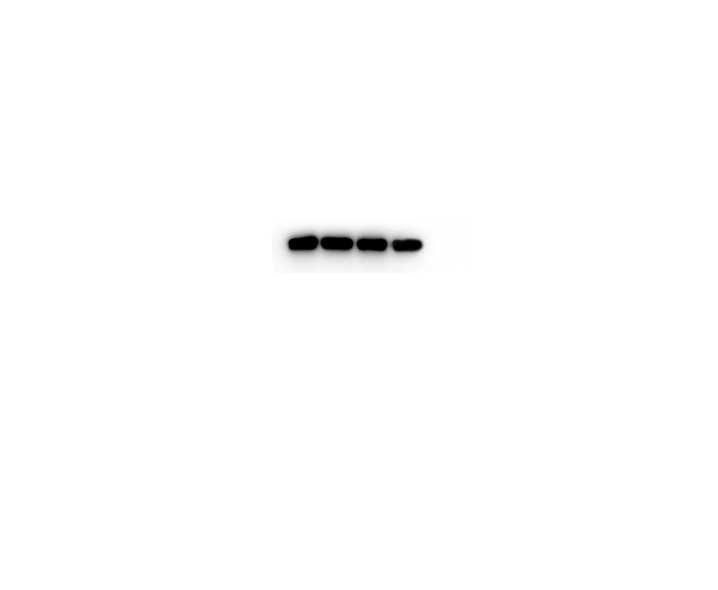

Supplement: Supplementary file 2 [file Data_Sheet_1.zip › supplementary material/Figure7A-GAPDH-MAVS.tif]

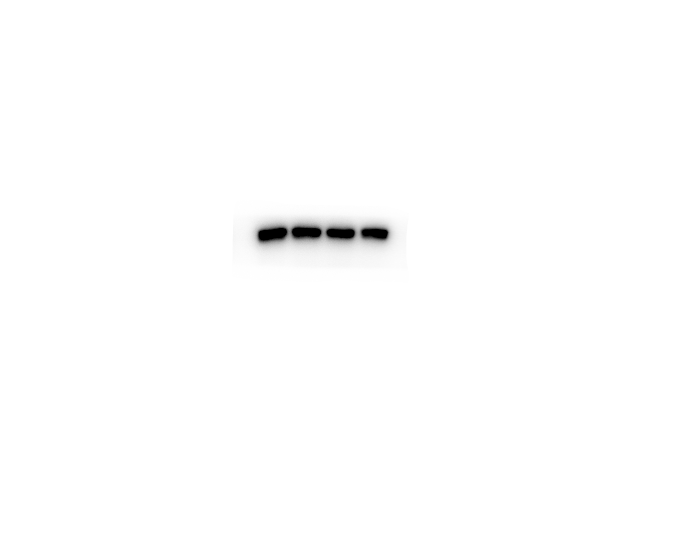

Supplement: Supplementary file 2 [file Data_Sheet_1.zip › supplementary material/Figure7A-GAPDH-MDA5.tif]

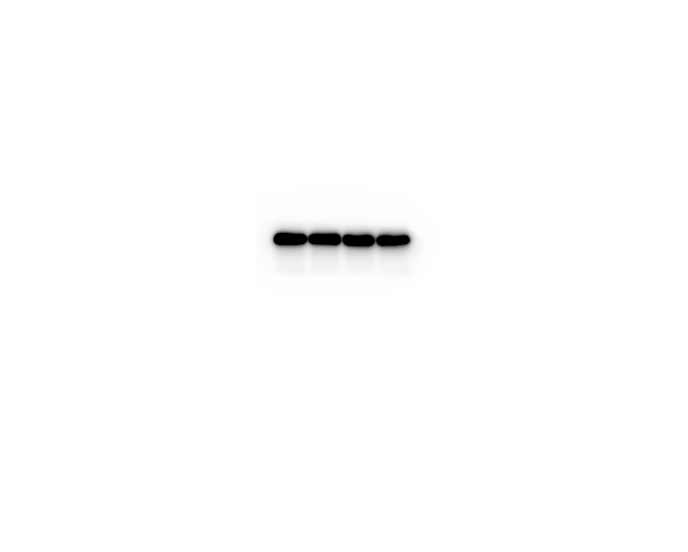

Supplement: Supplementary file 2 [file Data_Sheet_1.zip › supplementary material/Figure7A-GAPDH-RIG-I.tif]

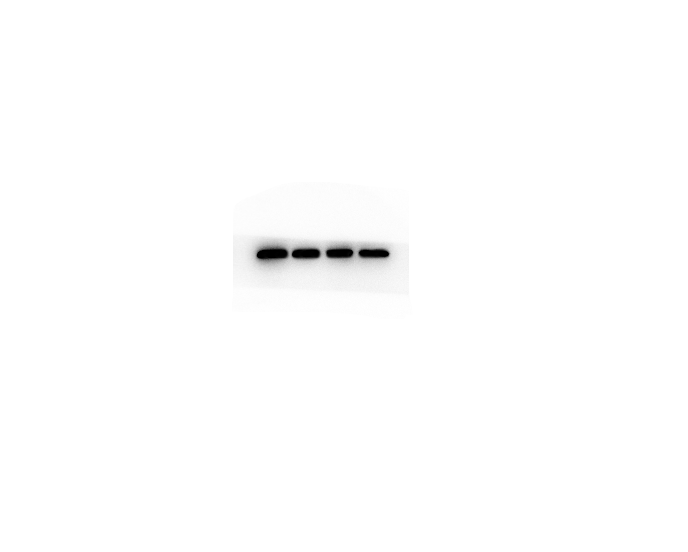

Supplement: Supplementary file 2 [file Data_Sheet_1.zip › supplementary material/Figure7A-GAPDH-TBK1.tif]

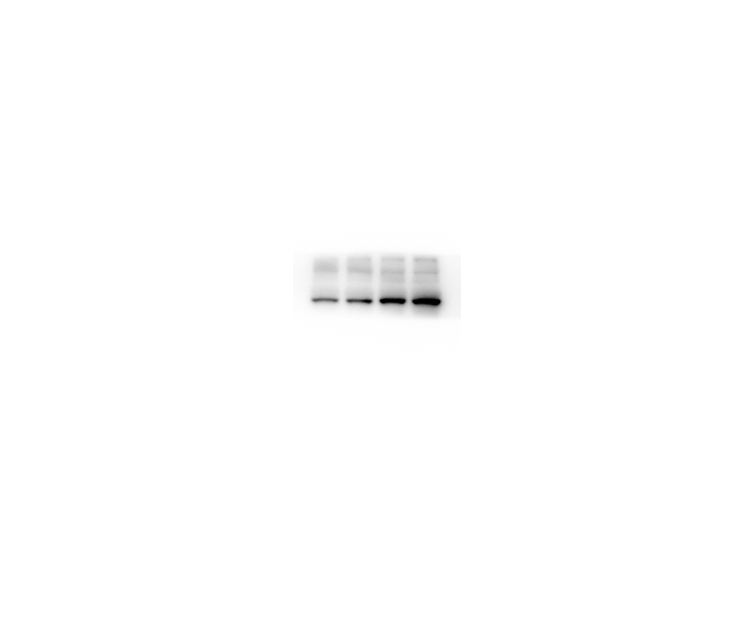

Supplement: Supplementary file 2 [file Data_Sheet_1.zip › supplementary material/Figure7A-SERINC5-IRF3.tif]

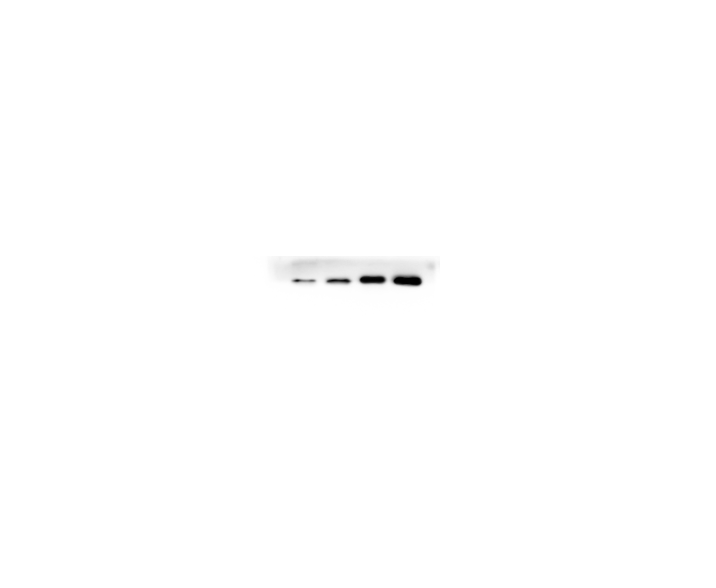

Supplement: Supplementary file 2 [file Data_Sheet_1.zip › supplementary material/Figure7A-SERINC5-IRF7.tif]

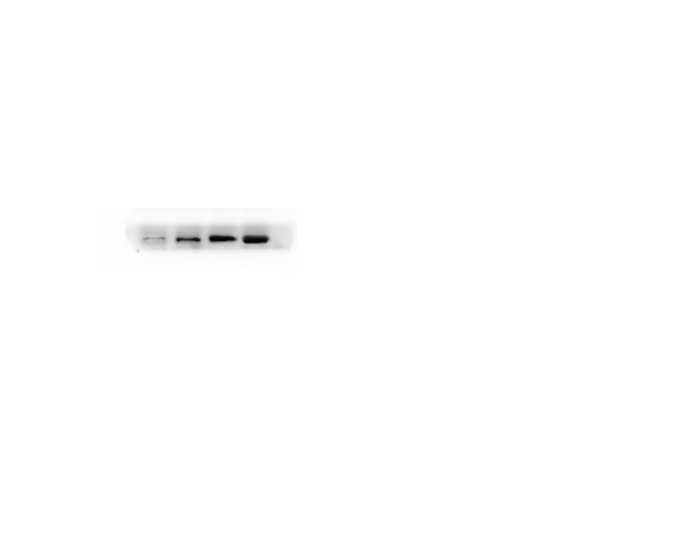

Supplement: Supplementary file 2 [file Data_Sheet_1.zip › supplementary material/Figure7A-SERINC5-MAVS.tif]

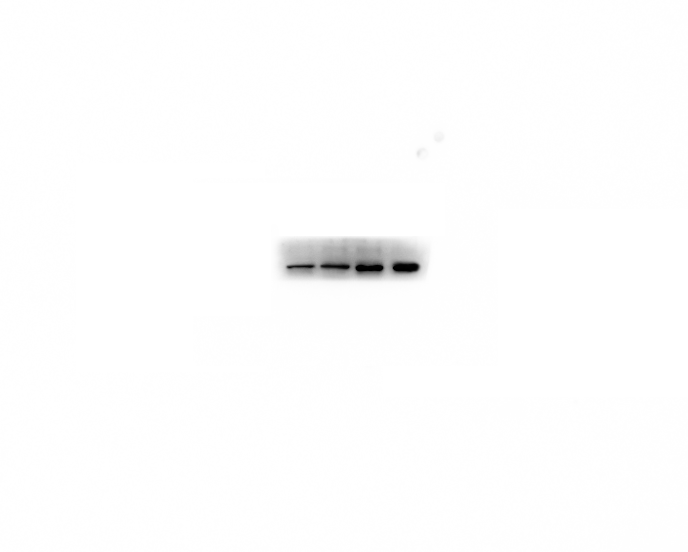

Supplement: Supplementary file 2 [file Data_Sheet_1.zip › supplementary material/Figure7A-SERINC5-MDA5.tif]

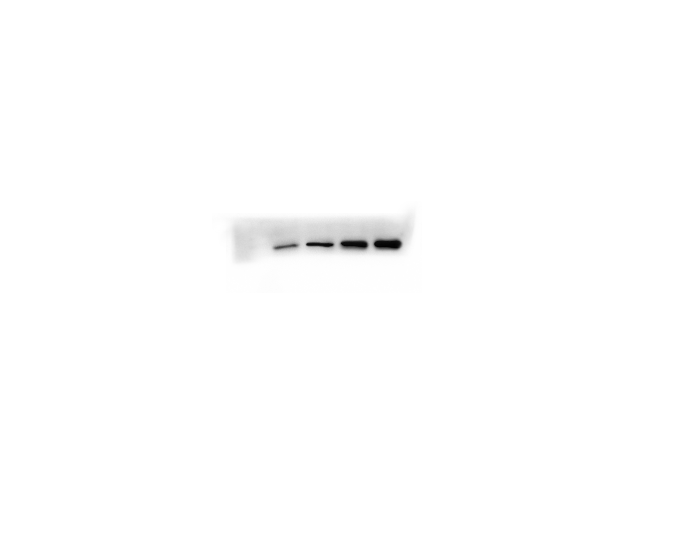

Supplement: Supplementary file 2 [file Data_Sheet_1.zip › supplementary material/Figure7A-SERINC5-RIG-I.tif]

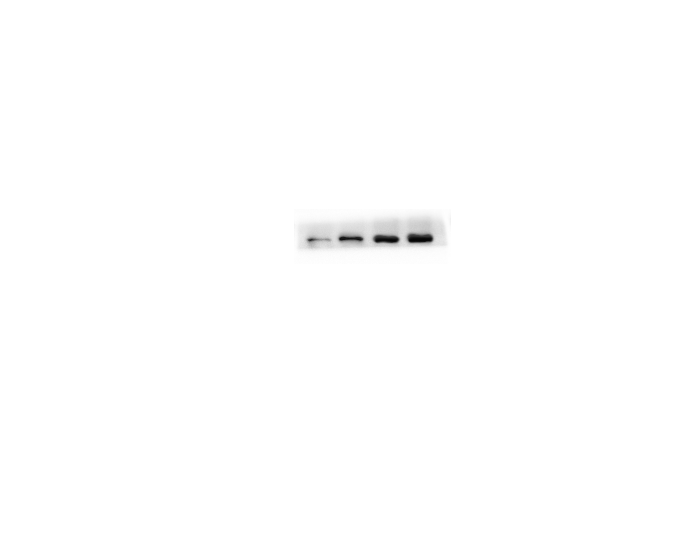

Supplement: Supplementary file 2 [file Data_Sheet_1.zip › supplementary material/Figure7A-SERINC5-TBK1.tif]

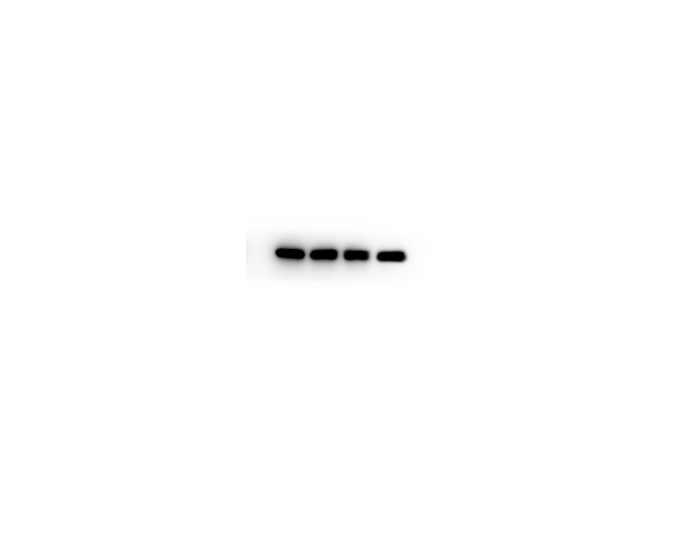

Supplement: Supplementary file 2 [file Data_Sheet_1.zip › supplementary material/Figure7B-GAPDH-IRF3.tif]

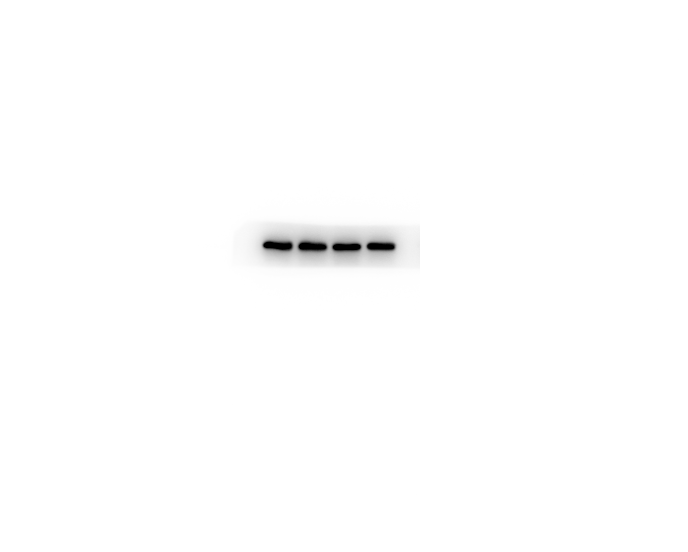

Supplement: Supplementary file 2 [file Data_Sheet_1.zip › supplementary material/Figure7B-GAPDH-IRF7.tif]

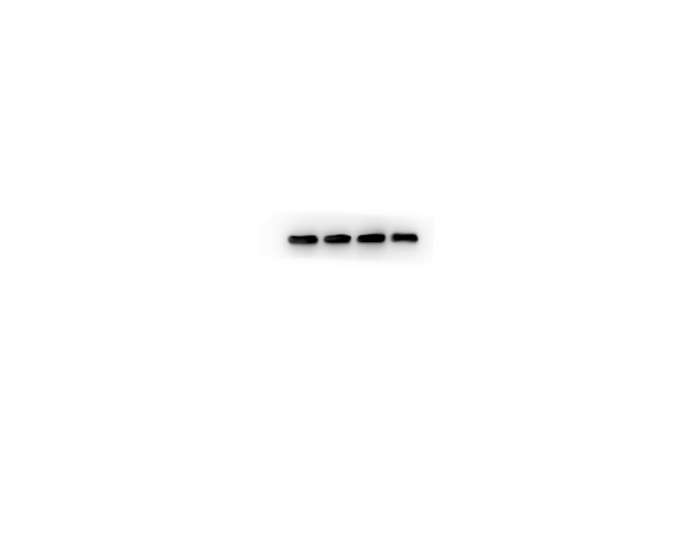

Supplement: Supplementary file 2 [file Data_Sheet_1.zip › supplementary material/Figure7B-GAPDH-MAVS.tif]

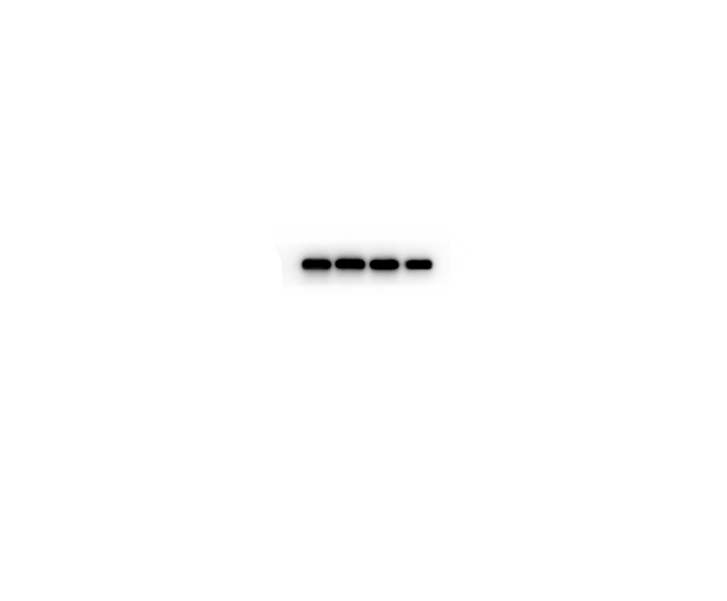

Supplement: Supplementary file 2 [file Data_Sheet_1.zip › supplementary material/Figure7B-GAPDH-MDA5.tif]

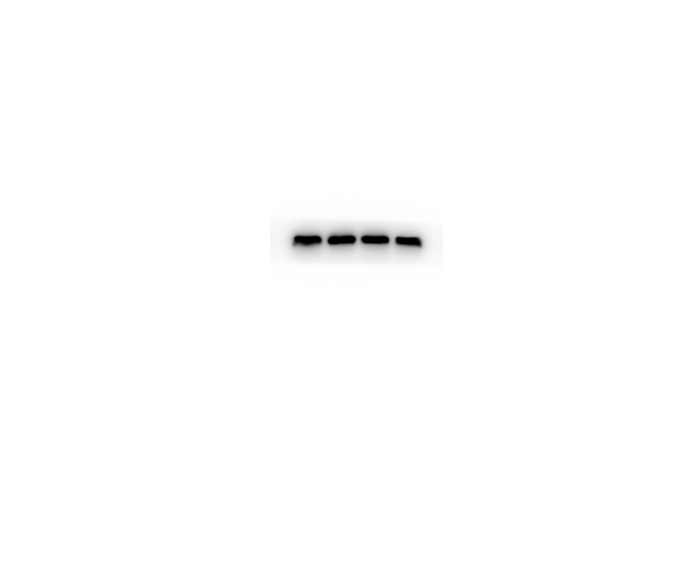

Supplement: Supplementary file 2 [file Data_Sheet_1.zip › supplementary material/Figure7B-GAPDH-RIG-I.tif]

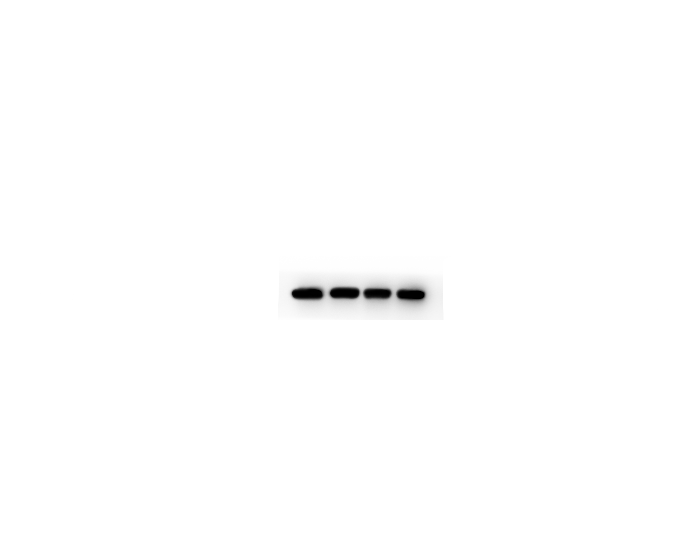

Supplement: Supplementary file 2 [file Data_Sheet_1.zip › supplementary material/Figure7B-GAPDH-TBK1.tif]

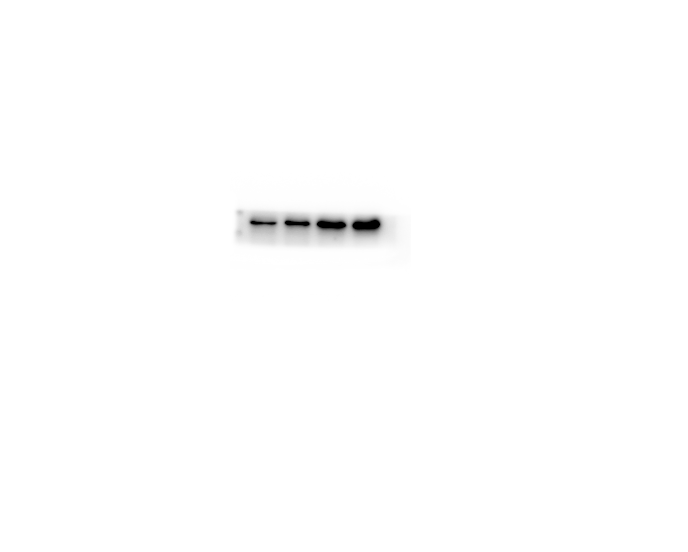

Supplement: Supplementary file 2 [file Data_Sheet_1.zip › supplementary material/Figure7B-SERINC5-IRF3.tif]

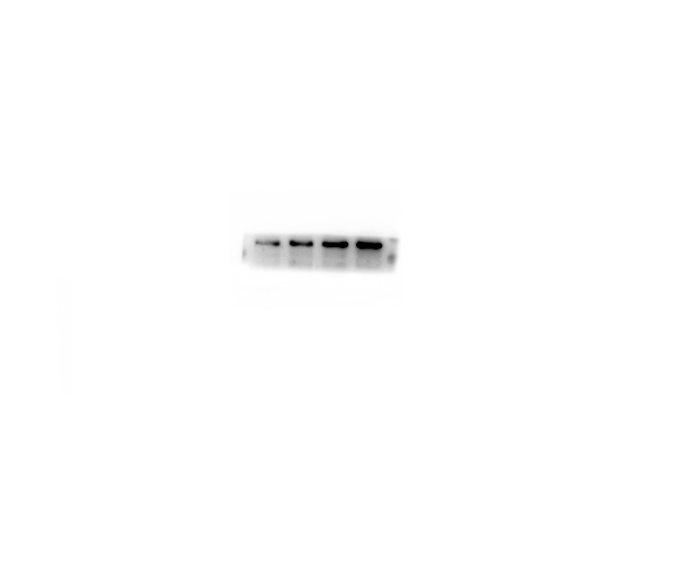

Supplement: Supplementary file 2 [file Data_Sheet_1.zip › supplementary material/Figure7B-SERINC5-IRF7.tif]

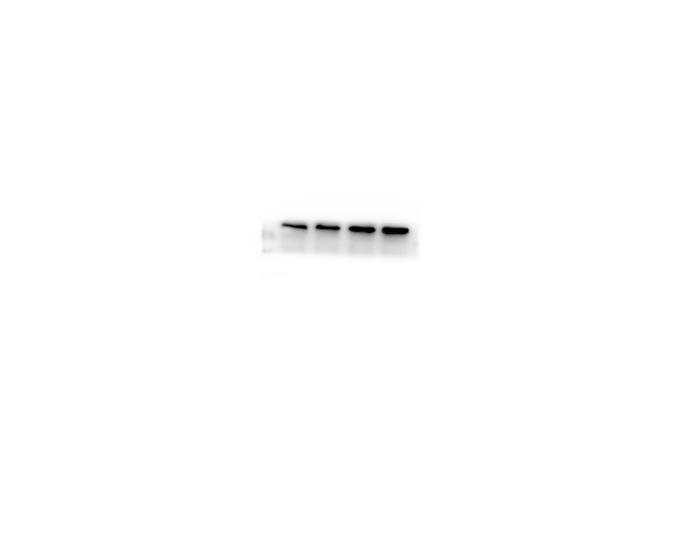

Supplement: Supplementary file 2 [file Data_Sheet_1.zip › supplementary material/Figure7B-SERINC5-MAVS.tif]

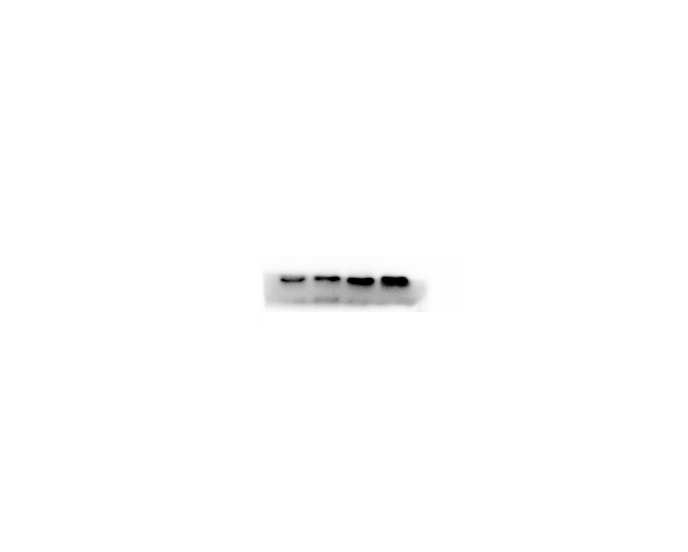

Supplement: Supplementary file 2 [file Data_Sheet_1.zip › supplementary material/Figure7B-SERINC5-MDA5.tif]

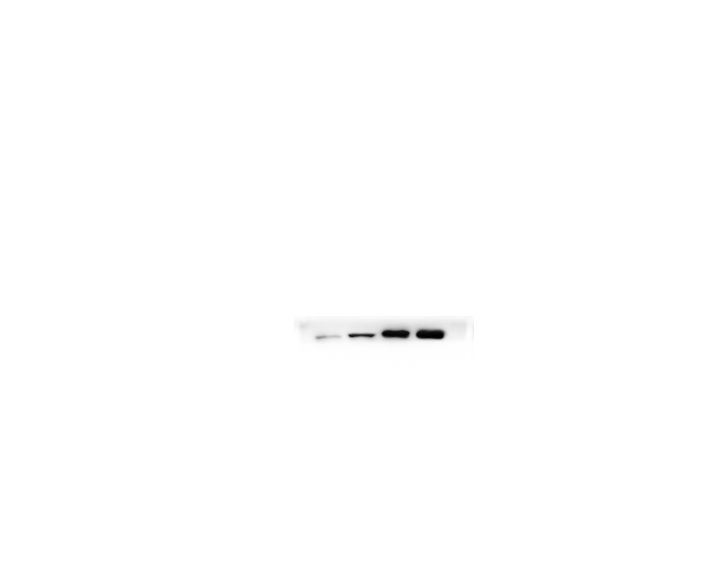

Supplement: Supplementary file 2 [file Data_Sheet_1.zip › supplementary material/Figure7B-SERINC5-RIG-I.tif]

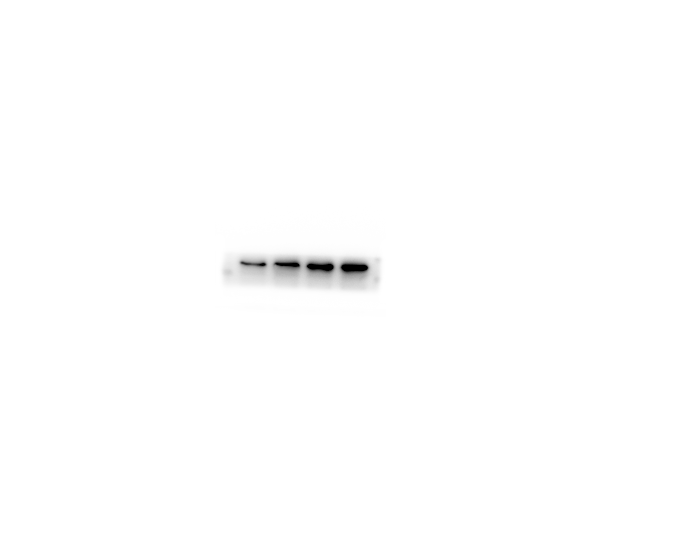

Supplement: Supplementary file 2 [file Data_Sheet_1.zip › supplementary material/Figure7B-SERINC5-TBK1.tif]

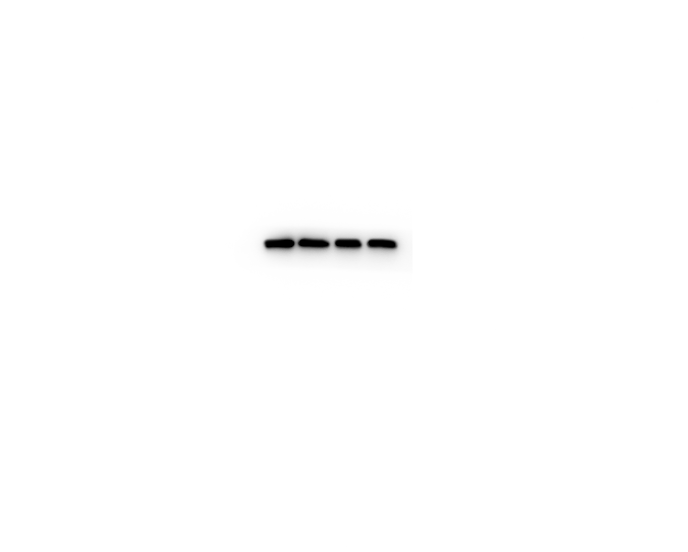

Supplement: Supplementary file 2 [file Data_Sheet_1.zip › supplementary material/Figure7C-GAPDH-CMV.tif]

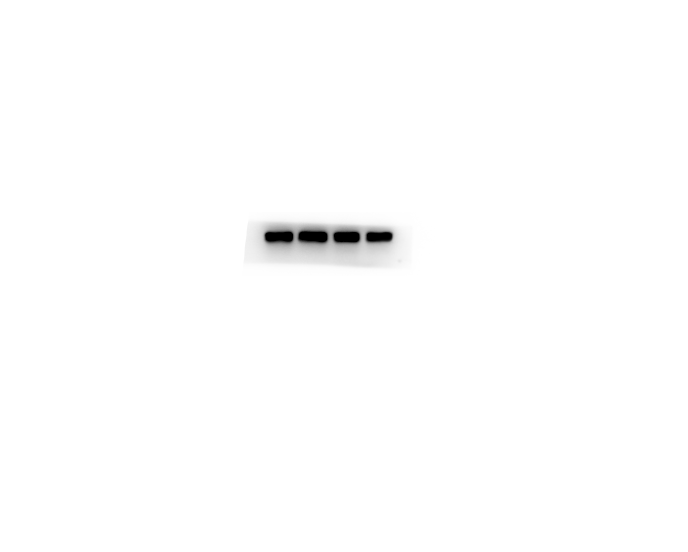

Supplement: Supplementary file 2 [file Data_Sheet_1.zip › supplementary material/Figure7C-GAPDH-MDA5.tif]

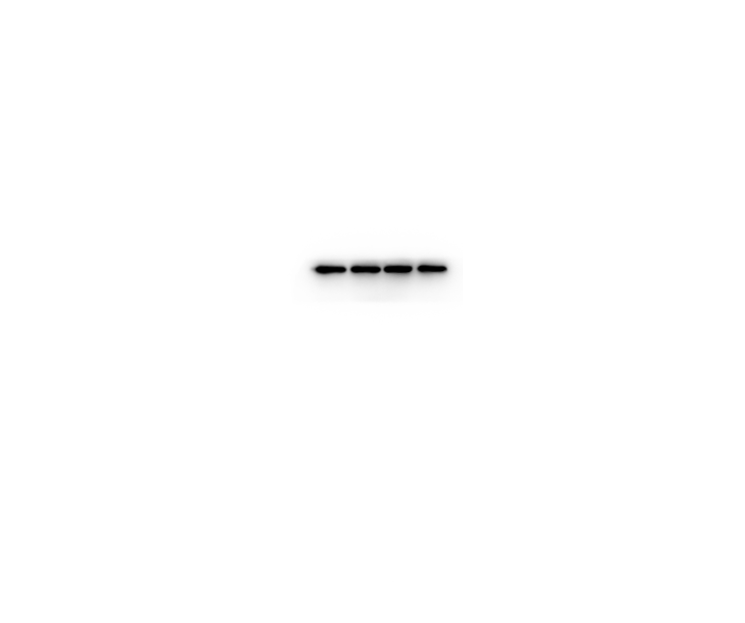

Supplement: Supplementary file 2 [file Data_Sheet_1.zip › supplementary material/Figure7C-GAPDH-RIG-I.tif]

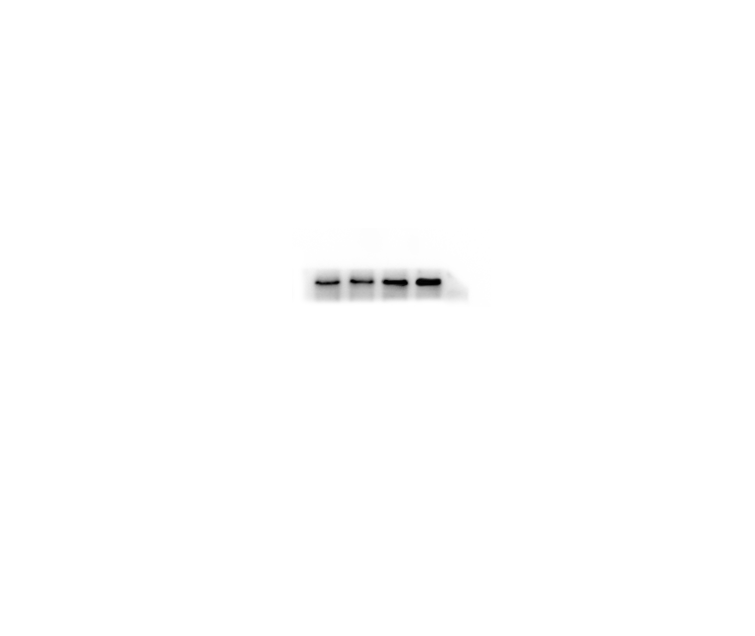

Supplement: Supplementary file 2 [file Data_Sheet_1.zip › supplementary material/Figure7C-SERINC5-CMV.tif]

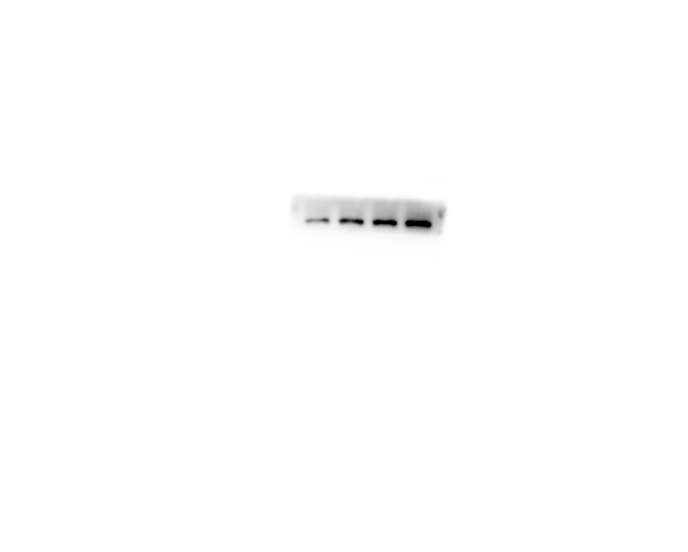

Supplement: Supplementary file 2 [file Data_Sheet_1.zip › supplementary material/Figure7C-SERINC5-MDA5.tif]

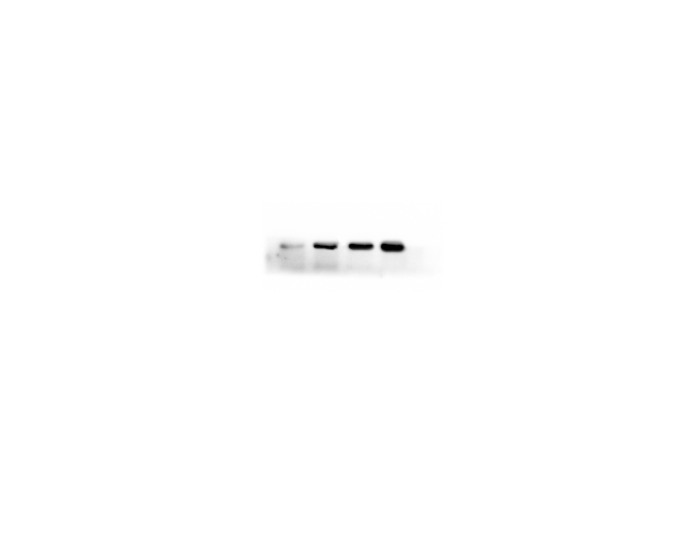

Supplement: Supplementary file 2 [file Data_Sheet_1.zip › supplementary material/Figure7C-SERINC5-RIG-I.tif]

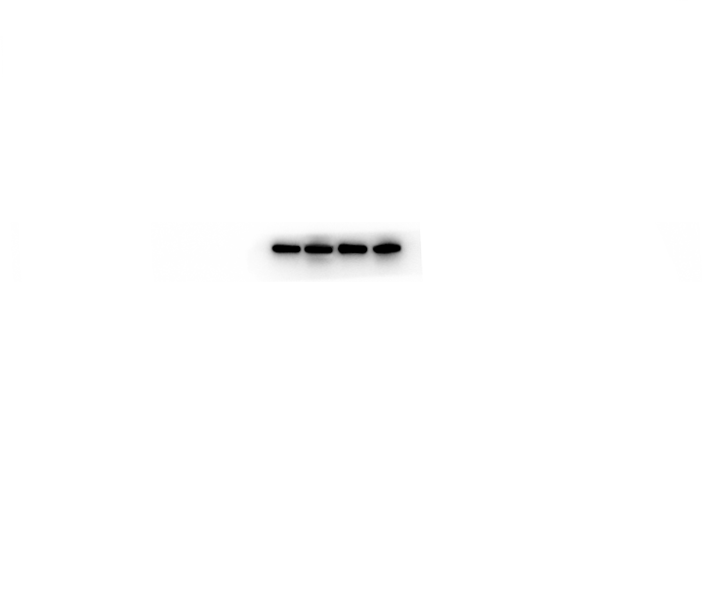

Supplement: Supplementary file 2 [file Data_Sheet_1.zip › supplementary material/Figure7D-GAPDH-CMV.tif]

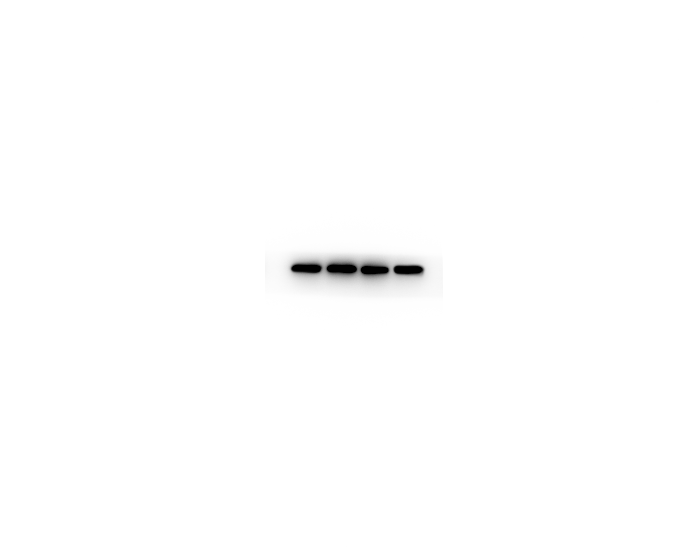

Supplement: Supplementary file 2 [file Data_Sheet_1.zip › supplementary material/Figure7D-GAPDH-MDA5.tif]

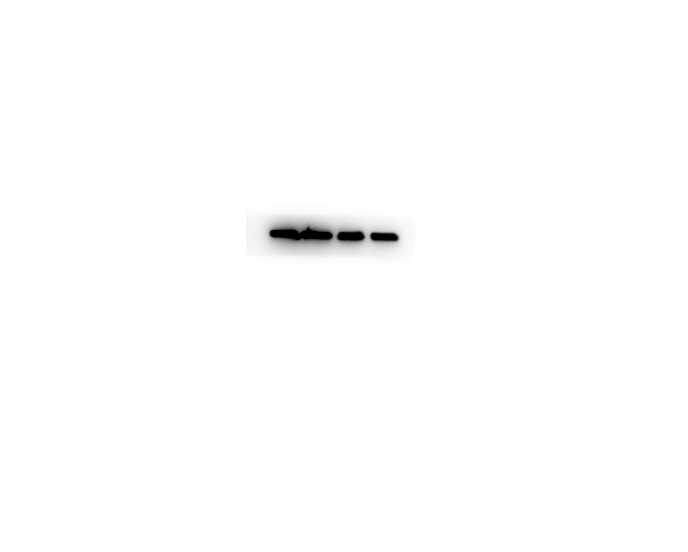

Supplement: Supplementary file 2 [file Data_Sheet_1.zip › supplementary material/Figure7D-GAPDH-RIG-I.tif]
